# Insulin-like growth factor 2 drives fibroblast-mediated tumor immunoevasion and confers resistance to immunotherapy

**DOI:** 10.1172/JCI183366

**Published:** 2024-11-15

**Authors:** Daqiang Song, Yushen Wu, Jie Li, Jiazhou Liu, Ziying Yi, Xiaoyu Wang, Jiazheng Sun, Liuying Li, Qianxue Wu, Yuru Chen, Huiying Fang, Tiankuo Luan, Huimin Du, Jing Huang, Weiyan Peng, Yuxian Wei, Fan Li, Qin Li, Li Zhang, Yong Zhu, Jingyuan Wan, Guosheng Ren, Hongzhong Li

**Affiliations:** 1 Chongqing Key Laboratory of Molecular Oncology and Epigenetics, The First Affiliated Hospital of Chongqing Medical University, Chongqing, China.; 2 Department of Pharmacology, Chongqing Medical University, Chongqing, China.; 3 Department of Oncology,; 4 Department of Breast and Thyroid Surgery, and; 5 Department of Respiratory, The First Affiliated Hospital of Chongqing Medical University, Chongqing, China.; 6 Department of Oncology, Beijing Friendship Hospital, Capital Medical University, Beijing, China.; 7 Department of Pathophysiology and; 8 Research Institute of Life Sciences, Chongqing Medical University, Chongqing, China.

**Keywords:** Oncology, Cancer immunotherapy

## Abstract

T cell exclusion is crucial in enabling tumor immune evasion and immunotherapy resistance. However, the key genes driving this process remain unclear. We uncovered a notable increase of insulin-like growth factor 2 (IGF2) in immune-excluded tumors, predominantly secreted by cancer-associated fibroblasts (CAFs). Using mice with systemic or fibroblast-specific deletion of IGF2, we demonstrated that IGF2 deficiency enhanced the infiltration and cytotoxic activity of CD8^+^ T cells, leading to a reduction in tumor burden. Integration of spatial and single-cell transcriptomics revealed that IGF2 promoted interaction between CAFs and T cells via CXCL12 and programmed death ligand 1 (PD-L1). Mechanistically, autocrine IGF2 activated PI3K/AKT signaling by binding to the IGF1 receptor (IGF1R) on CAFs, which was required for the immunosuppressive functions of CAFs. Furthermore, genetic ablation of IGF2 or targeted inhibition of the IGF2/IGF1R axis with the inhibitor linsitinib markedly boosted the response to immune checkpoint blockade. Clinically, elevated levels of IGF2 in tumors or plasma correlated with an adverse prognosis and reduced efficacy of anti–programmed death 1 treatment. Together, these results highlight the pivotal role of IGF2 in promoting CAF-mediated immunoevasion, indicating its potential as a biomarker and therapeutic target in immunotherapy.

## Abstract 2

IGF2 serves as a key upstream factor driving fibroblast-mediated immunoevasion, positioning itself as a promising biomarker and target for cancer immunotherapy.

## Graphical abstract

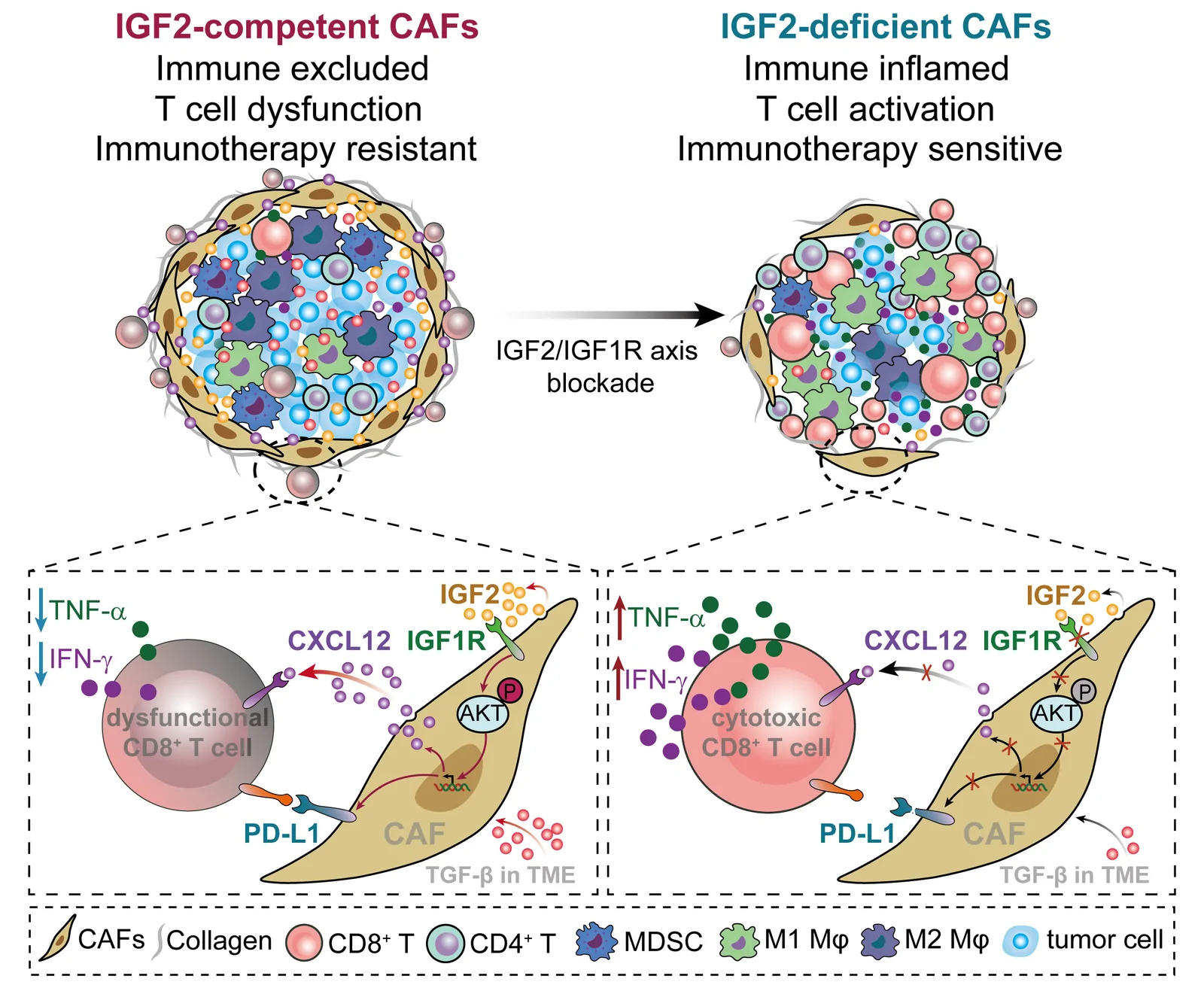

## Introduction

T cells play an essential role in identifying and eliminating cancer cells, making them a key component in cancer immunotherapy. Unfortunately, tumors frequently develop mechanisms to evade and suppress the immune response, leading to cancer immune evasion (1, 2). One potential strategy involves the use of immune checkpoint inhibitors to target essential immune-suppressive signals, notably programmed death 1 (PD-1) and cytotoxic T lymphocyte–associated protein 4 (CTLA-4). Despite this, only a fraction of patients tend to respond effectively to immune checkpoint blockade (ICB), especially in prevalent malignancies such as breast cancer and colorectal cancer (3–5). Hence, it is crucial to explore the potential mechanisms of tumor immune evasion for the development of innovative immunotherapy strategies and enhancement of clinical ICB responses. T cell exclusion and dysfunction are 2 primary mechanisms characterized by a lack of T cell infiltration and reduced T cell cytotoxicity, respectively (6). T cells are often restricted from entering the tumor microenvironment (TME), primarily because of the physical and biochemical barriers within the tumor. It was demonstrated that over 60% of triple-negative breast cancers (TNBCs) display this immune-excluded phenotype (7). Additionally, a substantial proportion of tumors in various other cancers, including colon cancer, also fall under the immune-excluded subtype, significantly correlating with unfavorable patient prognosis and ICB resistance when compared with the immune-inflamed type characterized by high T cell infiltrations (8, 9). Furthermore, T cells can become dysfunctional as a result of chronic antigen exposure or immunosuppressive factors in the TME (6). Several methods have been explored to target the crucial molecular pathways associated with these mechanisms to complement existing ICB therapies (10). It is crucial to emphasize that the presence of T cells in the TME is essential for achieving positive outcomes in ICB alone or in combination therapies. Therefore, delving deeper into the potential mechanisms underlying T cell exclusion can aid in understanding tumor immune evasion and addressing resistance to immunotherapy.

Cancer-associated fibroblasts (CAFs) are activated fibroblasts residing in the TME. Within this dynamic milieu, CAFs acquire distinct characteristics, undergo differentiation, and fulfill specialized functions that effectively support tumor growth and metastasis (11, 12). Emerging evidence demonstrates the close involvement of CAFs in T cell exclusion. It has been elucidated that CAFs influence T cell infiltration through the secretion of various chemokines and cytokines, such as CCL5 and CXCL12, orchestrating the dynamics of the tumor immune microenvironment (13, 14). Furthermore, CAFs can generate a dense extracellular matrix that physically hinders the movement of immune cells into the tumor. Additionally, the crosstalk between fibroblasts and T cells encompasses complex signaling pathways, including the PD-1/programmed death ligand 1 (PD-1/PD-L1) axis and the CD73/adenosine axis, which affects the functional characteristics and proliferation of T lymphocytes in the TME (15–17). CAFs additionally release immunosuppressive molecules like TGF-β and IL-10, which impede T cell activation, proliferation, and effector responses (18, 19). Despite the progressive elucidation of the mechanism by which CAFs mediate immune evasion in tumors, the fundamental pivotal genes or core regulatory mechanisms remain enigmatic.

Insulin-like growth factor 2 (IGF2), a member of the insulin-like growth factor family, is a secreted protein that regulates cell proliferation, survival, and differentiation (20). Although increasing studies suggest the significant role of elevated IGF2 in tumor malignancy, the focus has mainly been on its direct effects on tumor cells, with its role in TME and tumor immunity still unclear (2). Through the application of multiomics analyses, mouse models featuring whole-body or fibroblast-specific gene ablation, and a selective inhibitor, we discovered that IGF2 primarily originates from CAFs and functions as a critical upstream factor in facilitating CAF-mediated evasion of the immune response within tumors.

## Results

### Fibroblast-derived IGF2 shows a robust correlation with the pattern of T cell exclusion across various tumor types.

**Figure 1:**
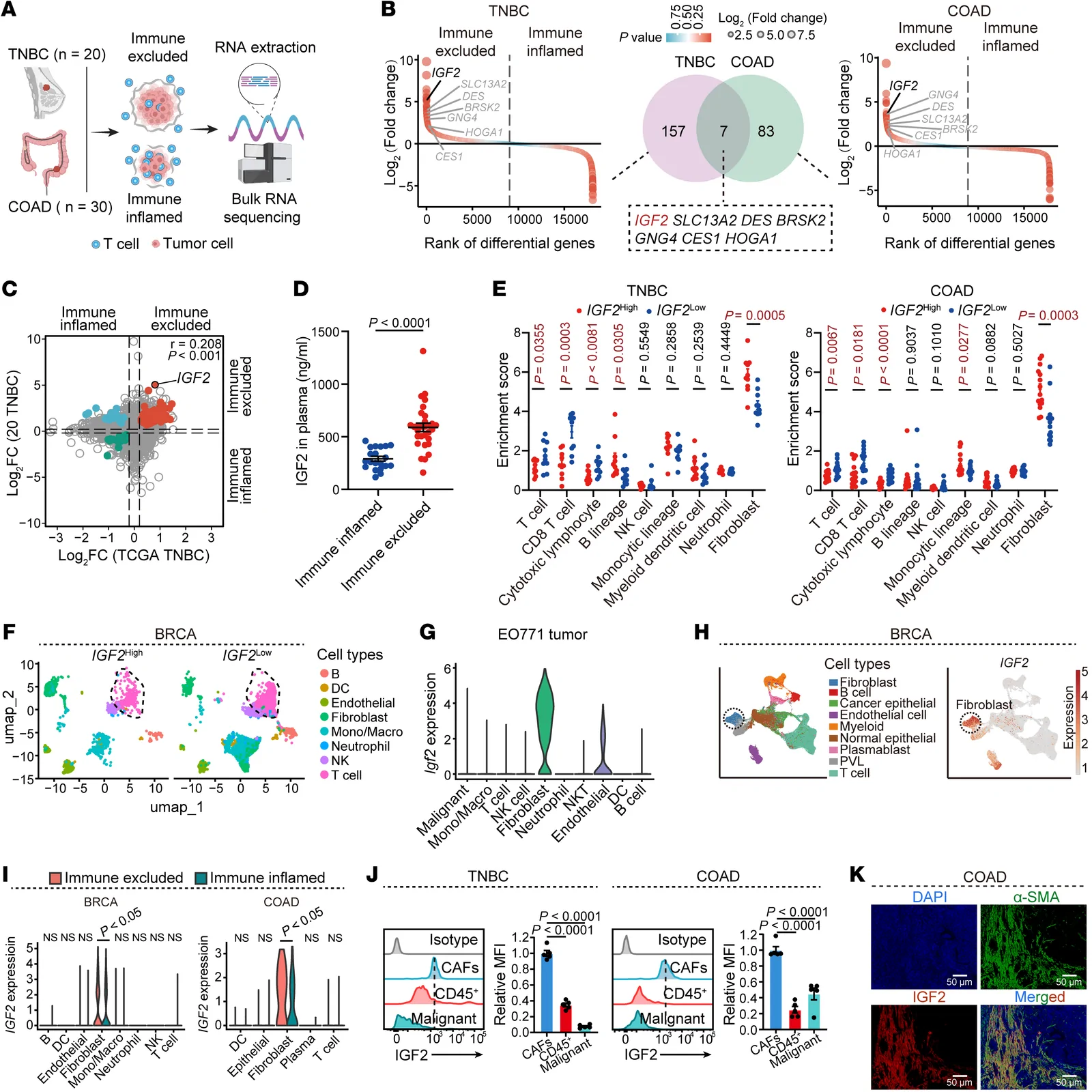
IGF2 correlates with T cell exclusion and is highly expressed in CAFs. (**A**) Experimental schematics of analysis of RNA-Seq. The schematic in **A** was created with BioRender.com (agreement no. WO27B3A3JY). (**B**) Rank of differential genes and shared genes from human TNBC and COAD. The tumor samples were categorized into immune-inflamed and immune-excluded groups according to CD3 staining. (**C**) Scatter plot of 9-quadrant association analyses of mRNA levels from log_2_ FC in both TCGA TNBC and our TNBC cohorts. (**D**) Plasma IGF2 levels from patients with TNBC with immune-inflamed (*n* = 20) or immune-excluded tumors (*n* = 30). (**E**) Enrichment score of stromal cells in the TME of human TNBC and COAD based on IGF2 expression. (**F**) The uniform manifold approximation and projection (UMAP) plot illustrating the distribution of cell clusters within the BRCA (GSE114727) TME based on high or low IGF2 expression. Different cell clusters are represented by distinct colors in the plot. (**G**) Expression levels of *Igf2* in the cell clusters in the TME of mammary EO771 tumors based on scRNA-Seq analysis. (**H**) scRNA-Seq analysis presenting the expression of *IGF2* in the cell clusters in the TME of BRCA (GEO GSE176078). PVL, perivascular-like cells. (**I**) Violin plot showing *IGF2* expression in the cell clusters within the immune-inflamed and immune-excluded BRCA (GSE114727) or COAD (GSE179784) tumors. (**J**) Flow cytometric analysis showing IGF2 expression in the CAFs, CD45^+^ immune cells, and malignant cells in the TME of TNBC or COAD tumors (*n* = 5). (**K)** Representative immunofluorescence microscopy images for α–smooth muscle actin (α-SMA) (green) and IGF2 (red) in human COAD tissues. Scale bars: 50 μm. Data indicate the mean ± SEM (**D**, **E**, and **J**). Significance was determined by 2-tailed, unpaired Student’s *t* test (**D** and **I**), 2-way ANOVA (**E**), and 1-way ANOVA (**J**). Pearson’s correlation coefficient was calculated for **C**. Mono, monocytes; Macro, macrophages.

To uncover the underlying mechanisms of T cell exclusion, we conducted transcriptome profile analysis on the tumors from patients with TNBC or colon adenocarcinoma (COAD) (Figure 1A and Supplemental Figure 1A; supplemental material available online with this article; https://doi.org/10.1172/JCI183366DS1). In TNBC, 164 genes showed significant upregulation in the immune-excluded tumors as compared with the immune-inflamed tumors, whereas in COAD, 90 genes displayed notable upregulation in the immune-excluded tumors in contrast to the immune-inflamed tumors. By intersecting these differential genes, 7 immune exclusion–related genes were identified: *IGF2*, *SLC13A2*, *DES*, *BRSK2*, *GNG4*, *CES1*, and *HOGA1*. Notably, in both TNBC and COAD, *IGF2* emerged as the top gene, showing a significant fold change (FC) between the immune-excluded and immune-inflamed tumors (Figure 1B). Furthermore, an integrated analysis revealed a substantial positive correlation between the FC of *IGF2* in The Cancer Genome Atlas (TCGA) cohorts and that in clinical in-house cohorts (Figure 1C and Supplemental Figure 1B). Additionally, a comprehensive analysis using the tumor immune dysfunction and exclusion (TIDE) system was conducted on 4,028 tumor samples from 9 cancer types of TCGA database, indicating significantly higher *IGF2* expression in the immune-excluded tumors (Supplemental Figure 1C). Moreover, a substantial increase in plasma IGF2 levels was observed in patients with TNBC who had immune-excluded tumors (Figure 1D). Subsequently, a comparative analysis of the TME was performed to investigate the distinctions in the immune milieu between tumors characterized by high IGF2 expression and those with low levels of IGF2. In our TNBC and COAD cohorts, we observed that IGF2 expression was negatively correlated with the abundance of total T cells, CD8^+^ T cells, and cytotoxic lymphocytes, but positively correlated with fibroblast infiltration (Figure 1E). Similar results were also demonstrated in TNBC, COAD, and 5 other cancer types from TCGA database, showing a significantly increased enrichment in fibroblasts and M0 macrophages in IGF2^hi^ samples, along with a reduction in CD8^+^ T cells and M1 macrophages in the TME (Supplemental Figure 1D). Furthermore, the single-cell RNA-Seq (scRNA-Seq) analysis of breast cancer (BRCA) revealed increased T cell infiltration in tumors with low IGF2 expression compared with those with high IGF2 levels (Figure 1F). Together, these clinical data suggest the potential involvement of IGF2 in T cell exclusion.

To comprehensively investigate the function of IGF2, we then identified the specific cell populations that express IGF2 within the TME. We observed heightened IGF2 expression in the stroma of immune-excluded TNBC tumors (Supplemental Figure 2A). Based on the Tumor Immune Estimation Resource (TIMER) database, we identified a positive correlation between IGF2 expression and the infiltration level of CAFs in both BRCA and COAD (Supplemental Figure 2B). Furthermore, scRNA-Seq analysis revealed that IGF2 was mainly expressed in CAFs but not in other cell populations within mammary EO771 tumors and various human cancers, including BRCA, COAD, and lung adenocarcinoma (LUAD) (Figure 1, G–I, and Supplemental Figure 2, C and D). Notably, IGF2 expression was markedly elevated in fibroblasts from LUAD compared with expression in fibroblasts from adjacent lung tissue. In line with the aforementioned results (Figure 1, B–E, and Supplemental Figure 1, B and C), the expression of IGF2 in the CAFs was higher in immune-excluded tumors than in immune-inflamed tumors (Figure 1I). Flow cytometric analysis showed elevated IGF2 expression in the CAFs compared with expression levels in immune cells (CD45^+^ subsets) or malignant cells in both human and murine tumor tissues (Figure 1J and Supplemental Figure 2E). Consistently, immunofluorescence (IF) staining confirmed the predominant expression of IGF2 in CAFs (Figure 1K), whereas Western blot analysis unveiled higher levels of IGF2 protein in CAFs compared with levels in human or murine tumor cell lines (Supplemental Figure 2F). Together, these findings suggest that IGF2 predominantly originated from CAFs in the TME.

Next, we interrogated the factors in the TME that could upregulate IGF2 expression in CAFs. In our TNBC cohort, we identified multiple signaling pathways significantly enriched in immune-excluded tumors compared with immune-inflamed tumors, particularly involving the response to TGF-β (Supplemental Figure 2G). Elevated expression of *TGFB1* was evident in immune-excluded TNBC tumors compared with immune-inflamed TNBC tumors (Supplemental Figure 2H). Upon analysis of TCGA database, we noticed a positive correlation between the expression of *IGF2* and *TGFB1* (Supplemental Figure 2I). Moreover, through analysis of both scRNA-Seq data and bulk RNA-Seq data for fibroblasts, we observed significantly increased TGF-β signaling in WT CAFs compared with IGF2-KO (*Igf2^–/–^*) CAFs (Supplemental Figure 2J). These data suggest that TGF-β1 in the TME might serve as a potential upstream regulator promoting IGF2 expression in CAFs. Indeed, our analysis using Western blotting and flow cytometry demonstrated that recombinant TGF-β1 protein notably increased IGF2 expression in both human and murine CAFs in a time- and dose-dependent manner (Supplemental Figure 2, K–M).

### Inhibition of IGF2 in fibroblasts promotes T cell infiltration and enhances antitumor function.

To further investigate the effect of CAF-derived IGF2 on T cell exclusion, we constructed IGF2-KO (*Igf*2*^–/–^*) C57BL/6 mice and confirmed the depletion efficacy of IGF2 in CAFs (Supplemental Figure 3, A–C). Under steady-state conditions, *Igf2^–/–^* mice displayed normal T cell homeostasis in the spleen and lymph nodes (Supplemental Figure 3D). Next, we initially isolated CAFs from tumors of WT and *Igf2^–/–^* C57BL/6 mice. We then cocultured them with splenic T cells in a Transwell system to evaluate T cell migration efficiency. KO of IGF2 significantly negated the CAF-mediated inhibition of T cell migration (Supplemental Figure 3E). Similar results were obtained following knockdown of IGF2 expression in human CAFs using shRNA transfection (Supplemental Figure 3, F and G). Additionally, pretreatment of CAFs with the IGF2 pathway inhibitor linsitinib markedly reduced the CAF-mediated inhibition of T cell migration. In contrast, pretreatment of *Igf2^–/–^* or sh*IGF2* CAFs with recombinant IGF2 protein (rIGF2) restored this effect (Supplemental Figure 3, E and G).

**Figure 2:**
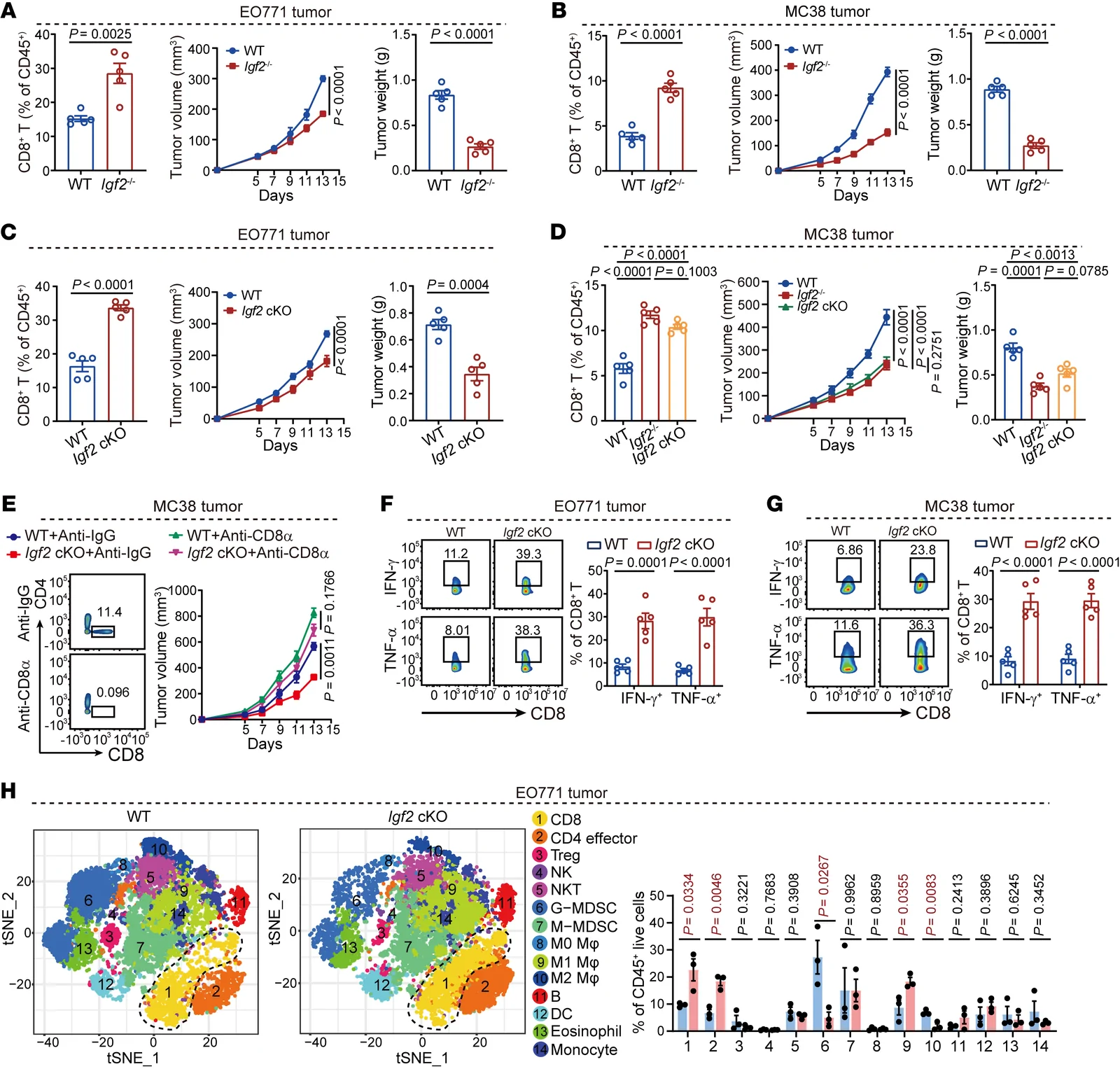
IGF2 deficiency significantly enhances T cell antitumor immunity. (**A**–**D**) Number of infiltrating CD8^+^ T cells, tumor growth, and tumor weight in EO771 tumors (**A** and **C**) or MC38 tumors (**B** and **D**) from WT, *Igf2^–/–^*, or *Igf2*-cKO mice (*n* = 5 mice per group). (**E**) Growth of MC38 tumors with the indicated treatment. CD8^+^ T cells were depleted by an anti-CD8α antibody (10 mg/kg) (*n* = 6–7 mice per group). (**F** and **G)** Percentage of IFN-γ^+^ or TNF-α^+^ CD8^+^ T cells in EO771 (**F**) or MC38 (**G**) tumors from WT or *Igf2*-cKO mice (*n* = 5 mice per group). (**H**) Plot of t-distributed stochastic neighbor embedding (tSNE) of tumor-infiltrating leukocytes overlaid with color-coded clusters (left) and the percentage of cell clusters (right) in EO771 tumors from WT or *Igf2*-cKO mice. NKT, NK T cell; M-MDSC, monocytic myeloid-derived suppressor cell; M0 Mφ, M0-like macrophage; M1 Mφ, M1-like macrophage; M2 Mφ, M2-like macrophage; B, B cell. Data are presented as the mean ± SEM (**A**–**H**). *P* values were determined by 2-way ANOVA (**A**–**H**), 2-tailed, unpaired Student’s *t* test (**A**–**C**), and 1-way ANOVA (**D**).

Subsequently, we explored the effects of IGF2 inhibition on both T cell infiltration and tumor progression in syngeneic mouse tumor models. EO771 mammary tumor cells or MC38 colon adenocarcinoma cells were implanted into *Igf2^–/–^* mice and WT mice. Remarkably, *Igf2^–/–^* mice exhibited heightened infiltration of CD8^+^ T cells and reduced tumor burden in comparison with WT mice (Figure 2, A and B, and Supplemental Figure 3H). To determine the role of CAF-derived IGF2 in the induction of T cell exclusion in vivo, we crossed *Igf2^fl/fl^* mice with the *S100a4*/fibroblast-specific protein 1 (FSP1)^CreERT^ strain to selectively deplete IGF2 in fibroblasts (*Igf2^fl/fl^ S100a4*^CreERT^ is referred to hereafter as *Igf2*-cKO) (Supplemental Figure 4A). The depletion efficacy of IGF2 in CAFs was validated through IF and Western blot analyses (Supplemental Figure 3, B and C). Additionally, under steady-state conditions, *Igf2*-cKO mice exhibited unaltered T cell homeostasis in the spleen and lymph nodes (Supplemental Figure 3D). Inoculation of EO771 tumor cells, MC38 tumor cells, and B16-F10 melanoma cells into *Igf2*-cKO mice revealed increased T cell infiltration and retarded tumor burden compared with that seen in WT mice, similar to *Igf2^–/–^* mice (Figure 2, C and D, and Supplemental Figure 4B). Remarkably, the suppression of tumor growth in *Igf2*-cKO or *Igf2^–/–^* mice was opposed by the removal of CD8^+^ T cells using anti-CD8 antibodies (Figure 2E andSupplemental Figure 4C), indicating that the enhanced antitumor activity by IGF2 blockade in CAFs depended on CD8^+^ T cells. To assess the role of IGF2 expressed in CD8^+^ T cells in these biological processes, we conducted a comparison between CD8^+^ T cells derived from *Igf2^–/–^* mice and those from WT mice. Our findings revealed comparable rates of migration, proliferation, and antitumor functionality between the 2 cell types (Supplemental Figure 4D), indicating that the augmented antitumor immune response following IGF2 blockade in mice was unlikely to stem from direct suppression of IGF2 within CD8^+^ T cells. To further elucidate the pivotal effect of IGF2 within CAFs, we cotransplanted CAFs with either control or sh*Igf2* constructs alongside various cancer cell lines into recipient mice. Cotransplantation of sh*Igf2* CAFs with mammary tumor cell 4T1 or colorectal cancer CT26 cells into BALB/c mice led to a marked increase in CD8^+^ T cell infiltration and a reduction in tumor growth when compared with the cotransplantation of control CAFs with cancer cells (Supplemental Figure 4, E and F). The administration of anti-CD8 antibody alleviated the inhibitory effect of the coimplantation of sh*Igf2* CAFs with 4T1, signifying that this effect depended on CD8^+^ T cells (Supplemental Figure 4G). Moreover, we found increased levels of IFN-γ and TNF-α generated by cytotoxic T cells in tumors from *Igf2*-cKO mice compared with tumors from WT mice, suggesting that IGF2 blockade also enhanced the antitumor activities of CD8^+^ T cells (Figure 2, F and G, and Supplemental Figure 5, A and B). Examination of bulk RNA-Seq data from the TNBC cohort in TCGA database revealed a negative correlation between the expression of IGF2 and the levels of T cell cytotoxic/cytolytic molecules (Supplemental Figure 5C).

To evaluate the effect of CAF-derived IGF2 on the TME, we analyzed CD45^+^ immune cells isolated from EO771 tumors in both WT and *Igf2*-cKO mice using mass cytometry (cytometry by time of flight [CyTOF]), leading to the identification of 14 distinct cell clusters (Supplemental Figure 5D). In line with the flow cytometric data (Figure 2C), EO771 tumors from *Igf2*-cKO mice demonstrated a notably increased number of CD8^+^ T cells (Figure 2H). Furthermore, fewer immunosuppressive cells, including granulocytic myeloid–derived suppressor cells (G-MDSCs) and M2 macrophages, were observed in the tumors from *Igf2*-cKO mice, as confirmed by flow cytometry (Figure 2H and Supplemental Figure 5E). Taken together, these findings suggest that IGF2 derived from CAFs contributed to the development of an immunosuppressive TME.

### scRNA-Seq and spatial transcriptomics analysis reveal the interaction between IGF2-educated CAFs and T cells.

**Figure 3:**
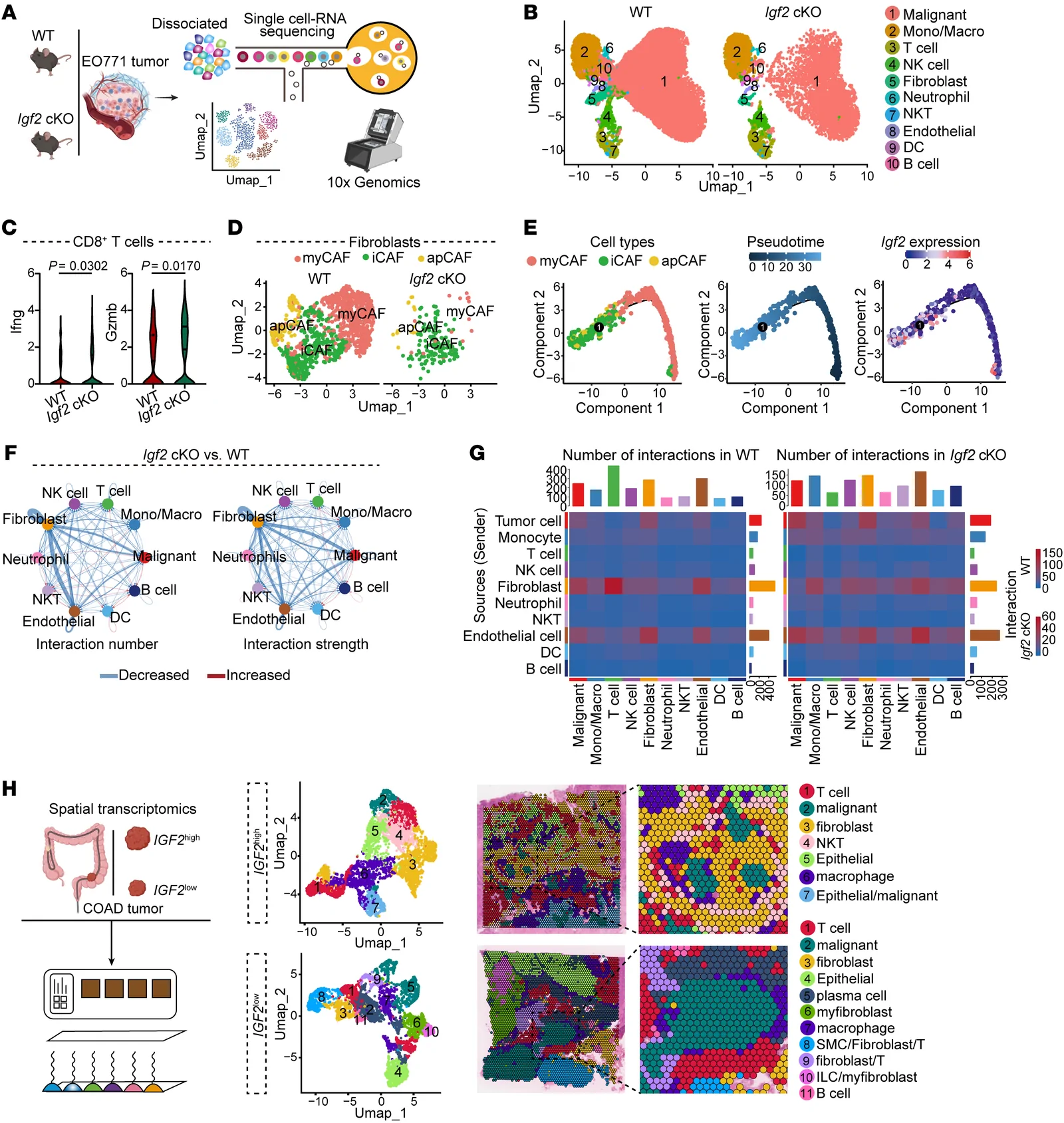
scRNA-Seq and stRNA-Seq analyses reveal the interaction between IGF2-educated CAFs and T cells. (**A**) Experimental schematics of scRNA-Seq of EO771 tumors from WT or *Igf2*-cKO mice (*n* = 3 mice per group). (**B**) Cell clusters identified and visualized with distinct color schemes in EO771 tumors based on scRNA-Seq analysis. (**C**) Expression levels of *Ifng* and *Gzmb* on the CD8^+^ T cell cluster from EO771 tumors based on scRNA-Seq analysis. (**D**) Fibroblast clusters identified and visualized with distinct color schemes in EO771 tumors based on scRNA-Seq analysis. (**E**) Trajectory analysis of 3 fibroblast types. Cell types were assigned different colors and arranged by pseudotime (left). Blue colors were based on pseudotime (middle). The change in *Igf2* expression in the cell types was based on pseudotime (right). (**F** and **G**) scRNA-Seq analysis showing the interaction among cell clusters in the TME of EO771 tumors from WT or *Igf2*-cKO mice (*n* = 3 mice per group). (**H**) Experimental schematics of stRNA-Seq analysis and unbiased clustering of spatial spots and definition of cell types in the COAD tissues. Some image parts in **A** and the process diagram in **H** were created with BioRender.com (agreement no. WO27B3A3JY). (**A** and **H**). Data are presented as the mean ± SEM. *P* values were determined by 2-tailed, unpaired Student’s *t* test (**C**).

To further elucidate the effect of CAF-derived IGF2 on the TME, we conducted scRNA-Seq analysis on mammary EO771 tumors from WT or *Igf2*-cKO mice (Figure 3A). In the EO771 tumors, a total of 10 distinct cell subsets were identified (Figure 3B and Supplemental Figure 6, A and B). We observed a significant reduction in the abundance of immunosuppressive cellular constituents, notably fibroblasts, monocytes/macrophages, and neutrophils, within the neoplastic milieu of *Igf2*-cKO mice compared with their WT counterparts. In contrast, an increase was observed in the antitumor cell populations, such as T cells and NK cells (Figure 3B and Supplemental Figure 6C). Additionally, the loss of IGF2 significantly enhanced the cytotoxic functions of CD8^+^ T cells, as evidenced by an increase in the expression of IFN-γ (*Ifng*) and granzyme B (*Gzmb*) (Figure 3C). These observations indicate that the loss of IGF2 in CAFs significantly shifted the cellular composition of the TME from a protumor to an antitumor state.

Furthermore, the effect of IGF2 on the CAFs was investigated by analyzing the fibroblast population. Within the fibroblast population, 3 distinct clusters were characterized, namely inflammatory fibroblasts (iCAFs), myofibroblasts (myCAFs), and antigen-presenting fibroblasts (apCAFs) (Figure 3D and Supplemental Figure 6, D and E). Remarkably, the deficiency of IGF2 markedly decreased the cell numbers of each CAF cluster (Figure 3D). To uncover the cell-state developmental trajectories of CAFs during cancer progression, we applied the Monocle algorithm to construct a pseudotime trajectory for individual cells (Supplemental Figure 6F). In the early and medium stages of pseudotime, the myCAFs clusters were found to be the predominant component of CAFs, whereas the iCAF and apCAF clusters emerged as the primary components in the late stage of pseudotime (Supplemental Figure 6G). Next, we performed a comprehensive analysis of *Igf2* gene expression patterns throughout the fibroblast trajectory. The results revealed higher expression levels of *Igf2* in iCAFs compared with expression levels in myCAFs and apCAFs (Figure 3E and Supplemental Figure 6H). As iCAFs are generally recognized to be tumor promoting via the secretion of inflammatory cytokines (21), this suggested that IGF2 could have a crucial role in controlling the secretion of cytokines from CAFs.

To further analyze the effect of IGF2 on cell-cell communication in the TME, we performed CellChat analysis based on scRNA-Seq data. IGF2 loss in CAFs markedly decreased the interaction number and strength among the cell clusters in the TME indicated in Figure 3F. Remarkably, we observed a diminished interaction between CAFs and T cells in the *Igf2*-cKO tumors in comparison with WT tumors (Figure 3G). To further validate the spatial interaction between CAFs and T cells, we conducted spatially resolved transcriptomics (stRNA-Seq) analysis on the IGF2^hi^ and IGF2^lo^ tumor tissues from patients with COAD. In the IGF2^hi^ tumor, fibroblasts were situated at the tumor nest periphery, in close proximity to T cells, thereby spatially segregating tumor cells from T cells. In contrast, the IGF2^lo^ tumor showed heightened instances of direct interaction between tumor cells and T cells (Figure 3H). These findings suggest that IGF2 might potentially enhance the signal flow from CAFs to T cells within the TME.

### IGF2 facilitates the interaction between CAFs and T cells via CXCL12 and PD-L1 signaling.

Emerging studies have revealed that CAFs and their remodeled extracellular matrix protein network can create a physical barrier that hinders T cell infiltration. To further clarify the mechanism by which CAF-derived IGF2 triggers T cell exclusion, we initially assessed the effect of IGF2 on CAF proliferation. In TCGA TNBC cohort, we observed greater enrichment of fibroblasts in the tumor tissues with high levels of IGF2 compared with those with low levels of IGF2 (Supplemental Figure 7A). *Igf2^–/–^* CAFs and sh*IGF2* CAFs exhibited a diminished proliferative capacity in comparison with their corresponding controls. In addition, linsitinib evidently inhibited fibroblast proliferation, whereas the introduction of rIGF2 significantly reversed the inhibitory effects resulting from IGF2 deficiency (Supplemental Figure 7, B and C). Furthermore, we conducted gene set enrichment analysis (GSEA) using RNA-Seq data from WT and *Igf2^–/–^* CAFs. Notably, collagen formation was significantly enriched in the WT CAFs (Supplemental Figure 7D). Through Picrosirius red staining, we observed a substantial reduction in collagen deposition in the tumors of *Igf2^–/–^* and *Igf2*-cKO mice (Supplemental Figure 7E). These findings indicate that the inhibition of IGF2 may disrupt the physical barrier formed by CAFs.

**Figure 4:**
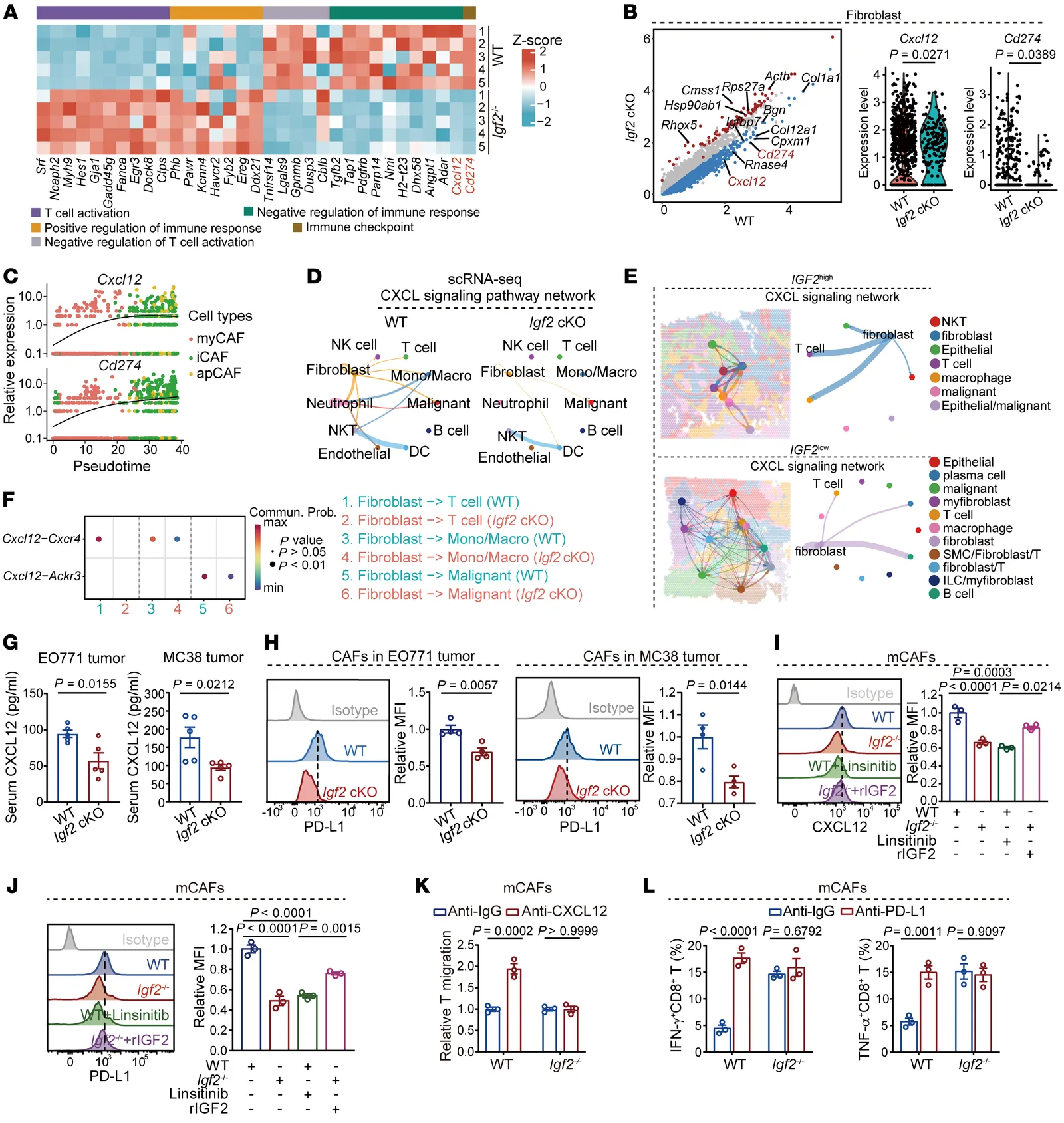
IGF2 facilitates the interaction between CAFs and T cells through CXCL12 and PD-L1 signaling. (**A**) Heatmap showing the transcripts of differentially expressed genes in WT and *Igf2^–/–^* CAFs (*n* = 5). (**B**) Volcano plot showing the expression of differentially expressed genes and violin plot representing the expression of *Cxcl12* and *Cd274* in a fibroblast cluster from EO771 tumors from WT or *Igf2*-cKO mice (*n* = 3 mice per group). (**C**) Expression changes of *Cxcl12* and *CD274* in the fibroblast subpopulations from EO771 tumors based on pseudotime analysis. (**D**) Network presenting CXCL signaling among cell clusters in EO771 tumors from WT or *Igf2*-cKO mice (*n* = 3 mice per group). The line thickness denotes the strength of the interactions. (**E**) stRNA-Seq analysis showing the CXCL signaling among cell clusters in the IGF2^hi^ or IGF2^lo^ COAD tissues. The line thickness denotes the strength of the interactions. SMC, smooth muscle cell. (**F**) Ligand-receptor interaction of CXCL12 with its receptors CXCR4 and ACKR3 in the indicated cell clusters in EO771 tumors from WT or *Igf2*-cKO mice (*n* = 3 mice per group). Commun., communication; Prob., probability; max, maximum; min, minimum. (**G**) Levels of serum CXCL12 in EO771 or MC38 tumor–bearing WT or *Igf2*-cKO mice were determined by ELISA (*n* = 5 mice per group). (**H**) PD-L1 expression on CAFs from EO771 or MC38 tumors was detected by flow cytometry (*n* = 5 mice per group). (**I** and **J**) Expression of CXCL12 (**I**) and PD-L1 (**J**) in the WT or *Igf2^–/–^* CAFs with or without linsitinib treatment (5 μM) or murine rIGF2 protein (10 μM) was detected by flow cytometry (*n* = 3). (**K**) Migration ratio of T cells cocultured with WT or *Igf2^–/–^* CAFs treated with anti-IgG or anti-CXCL12 neutralizing antibody (2 ng/mL). (**L**) Percentage of IFN-γ^+^ or TNF-α^+^ CD8^+^ T cells cocultured with WT or *Igf2^–/–^* CAFs treated with anti-IgG or anti–PD-L1 neutralizing antibody (0.2 μg/mL). Data are presented as the mean ± SEM (**B** and **G**–**L**). *P* values were determined by 2-tailed, unpaired Student’s *t* test (**B**, **G** and **H**), 1-way ANOVA (**I** and **J**), or 2-way ANOVA (**K** and **L**).

To gain deep insight into the IGF2 signaling responsible for the immunosuppressive phenotype of CAFs, we performed a comparative analysis of the global transcriptomic variances between *Igf2^–/–^* CAFs, sh*Igf2* CAFs, and their respective controls using RNA-Seq. We found that genes that positively regulate the immune response and T cell activation were upregulated in *Igf2^–/–^* and sh*Igf2* CAFs, while those related to immune suppression were downregulated (Figure 4A and Supplemental Figure 7F). It is noteworthy that the chemokine CXCL12, known to impede T cell infiltration (22), and the key immune checkpoint molecule PD-L1 (also known as CD274/PD-L1), was markedly reduced in *Igf2^–/–^* CAFs and sh*Igf2* CAFs (Figure 4A and Supplemental Figure 7F). Additionally, scRNA-Seq analysis demonstrated increased expression of CXCL12 and PD-L1 in fibroblasts from EO771 tumors from WT mice compared with those from *Igf2*-cKO mice (Figure 4B). The changes in expression of CXCL12 and PD-L1 along the pseudotime increased in the late pseudotime, with predominant expression in the iCAF cluster (Figure 4C), similar to IGF2 expression (Figure 3E). Moreover, scRNA-Seq data indicated that the loss of IGF2 in CAFs markedly reduced CXCL signaling between fibroblasts and T cells in the TME (Figure 4D). Consistently, the stRNA-Seq analysis revealed enhanced CXCL and PD-L1 signaling between fibroblasts and T cells in the IGF2^hi^ tumor (Figure 4E and Supplemental Figure 8A). Furthermore, we investigated the ligand-receptor interaction to analyze how CXCL signaling from CAFs acts on T cells. Analysis of stRNA-Seq data revealed a notable enhancement in the interaction intensity between CXCL12 and its predominant receptor CXCR4 on T cells in the IGF2^hi^ tumor, unlike in the IGF2^lo^ tumor (Supplemental Figure 8B). The scRNA-Seq analysis confirmed that the loss of IGF2 in CAFs evidently decreased the interaction strength of CXCL12-CXCR4 (Figure 4F). On the basis of these findings, we hypothesized that IGF2 might elevate the expression of CXCL12 and PD-L1 on CAFs, consequently hindering the infiltration and activity of T cells. Indeed, the absence of IGF2 resulted in a notable reduction in serum CXCL12 levels and membrane PD-L1 expression on CAFs in both EO771 and MC38 models (Figure 4, G and H). In vitro experiments also demonstrated that the depletion of IGF2 in CAFs or the use of linsitinib significantly suppressed the expression of CXCL12 and PD-L1, whereas the administration of rIGF2 significantly restored their expression (Figure 4, I and J). Similarly, the levels of CXCL12 and PD-L1 expression were markedly decreased in sh*IGF2* CAFs, which were effectively restored upon treatment with rIGF2 (Supplemental Figure 7G). Following pretreatment of WT CAFs with neutralizing antibodies targeting CXCL12 or PD-L1, followed by coculturing with T cells, we observed a restored migration capability and enhanced antitumor function, reaching levels comparable to those seen in cocultures with IGF2-deficient CAFs (Figure 4, K and L). Overall, these findings suggest that IGF2 may play a key role in shaping the immunosuppressive function of CAFs.

### IGF2 maintains the immunosuppressive function of CAFs by activating the PI3K/Akt pathway.

**Figure 5:**
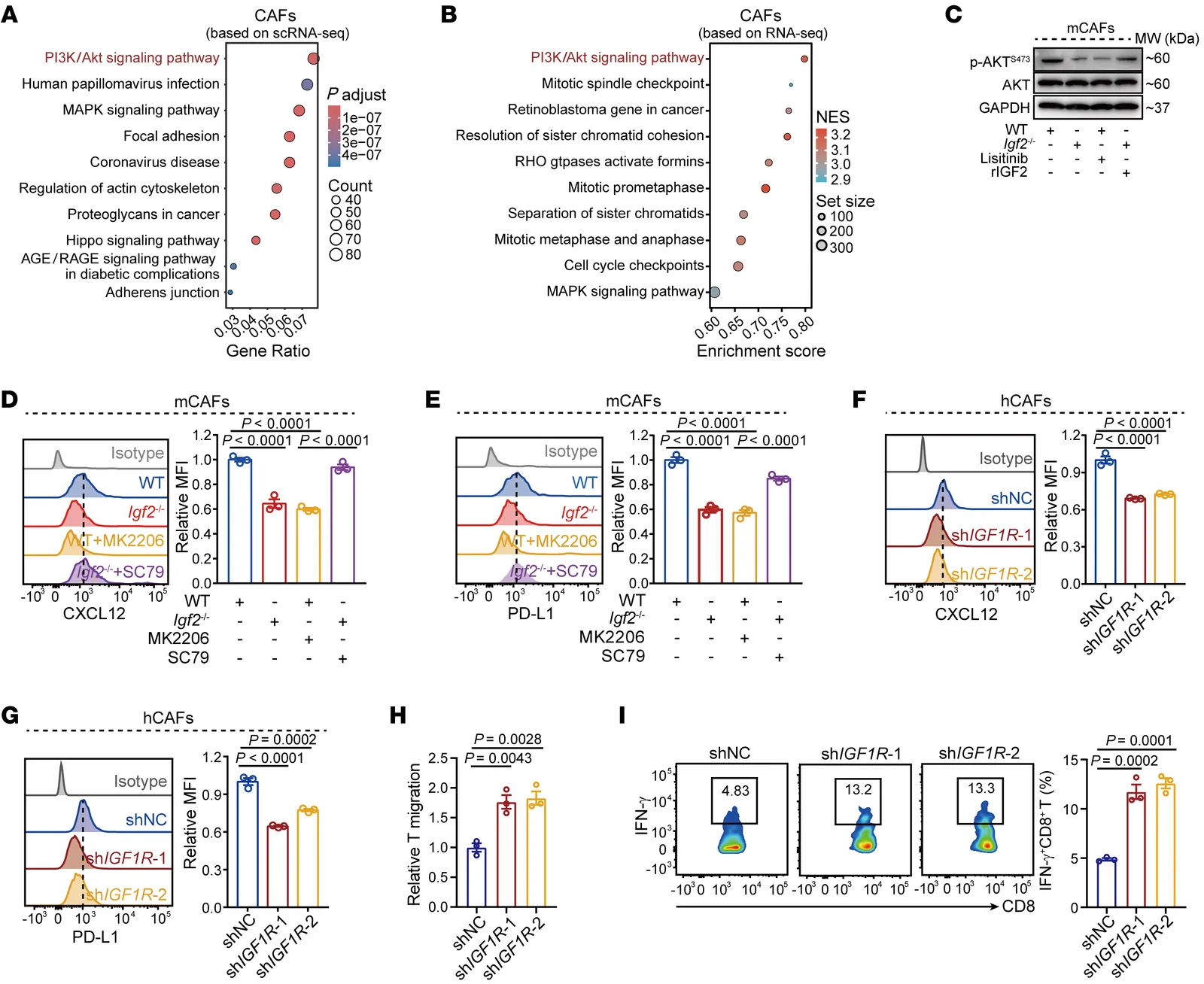
Deficiency of IGF2 significantly reduces the expression levels of CXCL12 and PD-L1 through the inactivation of Akt signaling in CAFs. (**A**) KEGG analysis of scRNA-Seq data showing the enriched signaling pathways in the fibroblasts from EO771 tumors of WT mice in comparison with those of *Igf2*-cKO mice (*n* = 3 mice per group). *P* adjust, adjusted *P* value. (**B**) KEGG analysis of RNA-Seq showing the enriched signaling pathways in WT CAFs compared with *Igf2^–/–^* CAFs. NES, normalized enrichment score. (**C)** Activation of the Akt pathway in WT or *Igf2^–/–^* CAFs treated with linsitinib (5 μM) or mouse rIGF2 protein (10 μM) was detected by Western blotting. (**D** and **E**) Expression levels of CXCL12 (**D**) and PD-L1 (**E**) on WT or *Igf2^–/–^* CAFs treated with MK2206 (10 μM) or SC79 (10 μM) were determined by flow cytometry (*n* = 3). (**F** and **G**) Expression levels of CXCL12 (**F**) and PD-L1 (**G**) on the negative control (shNC) or sh*IGF1R* human CAFs were determined by flow cytometry (*n* = 3). (**H)** Migration changes of CD8^+^ T cells cocultured with shNC or sh*IGF1R* human CAFs (*n* = 3). (**I**) The percentage of IFN-γ^+^ CD8^+^ T cells cocultured with shNC or sh*IGF1R* human CAFs was determined by flow cytometry (*n* = 3). Data are presented as the mean ± SEM (**D**–**I**). *P* values were determined by hypergeometric test (**A**) and 1-way ANOVA (**D**–**I**).

To investigate the modulation of immunosuppressive activities of CAFs by IGF2, we conducted Kyoto Encyclopedia of Genes and Genomes (KEGG) signaling pathway enrichment analysis of the stRNA-Seq data. Fibroblasts derived from tumor tissues with elevated levels of IGF2 displayed significant enrichment in specific signaling pathways, such as the PI3K/Akt and chemokine signaling pathways, as well as cytokine-cytokine receptor interaction (Supplemental Figure 9A). Concurrently, scRNA-Seq analysis revealed substantial PI3K/Akt signaling enrichment in the fibroblast cluster (Figure 5A). Further validation through RNA-Seq data analysis from both WT and *Igf2^–/–^* CAFs confirmed a substantial PI3K/Akt signaling enrichment in WT CAFs (Figure 5B). Additionally, analysis of The Cancer Proteome Atlas (TCPA) protein arrays demonstrated higher levels of phosphorylated AKT (p-AKT) at Ser 473 in the IGF2^hi^ group, indicating a positive association between IGF2 and the PI3K/Akt activation (Supplemental Figure 9B). Indeed, we observed a substantial suppression of the PI3K/Akt pathway in *Igf2^–/–^*, sh*IGF2*, or linsitinib-treated CAFs. In contrast, administration of rIGF2 restored the activation of this pathway (Figure 5C and Supplemental Figure 9, C and D). Following blockade of the PI3K/Akt pathway using the Akt inhibitor MK2206, we observed a notable inhibition of CAF proliferation and downregulation of CXCL12 and PD-L1 expression. Conversely, treatment with the Akt activator SC79 effectively reinstated the immunosuppressive effects that were impeded by IGF2 blockade (Supplemental Figure 9E and Figure 5, D and E). It is well documented that IGF2 binds to its primary receptor, IGF1R, and stimulates its intrinsic tyrosine kinase activity (23). Our findings also demonstrated that the introduction of rIGF2 or the IGF1R inhibitor linsitinib, respectively, enhanced or reduced the immunosuppressive activities of CAFs (Supplemental Figure 3E), implicating the potential involvement of IGF2 autocrine-mediated IGF1R activation in CAF function. In addition, GSEA unveiled a positive correlation between IGF2, IGF1R, CXCL12, and the PI3K/Akt signaling pathway (Supplemental Figure 9F). Strikingly, shRNA-mediated knockdown of the IGF1R resulted in the downregulation of this pathway (Supplemental Figure 9G). Knockdown of the IGF1R suppressed the proliferation of CAFs and reduced the expression of CXCL12 and PD-L1 on CAFs, ultimately diminishing the CAF-mediated restraint on T cell migration and function (Figure 5, F–I, and Supplemental Figure 9, H and I). Inoculation of 4T1 tumor cells with IGF1R-knockdown CAFs into BALB/c mice resulted in increased infiltration and antitumor activities of CD8^+^ T cells, as well as reduced tumor growth compared with the control group (Supplemental Figure 9, J–L). In summary, these results underscore the role of the IGF2/IGF1R axis in enhancing the immunosuppressive activities of CAFs through the promotion of PI3K/Akt signaling.

### Blocking the IGF2 pathway synergizes with ICB.

**Figure 6:**
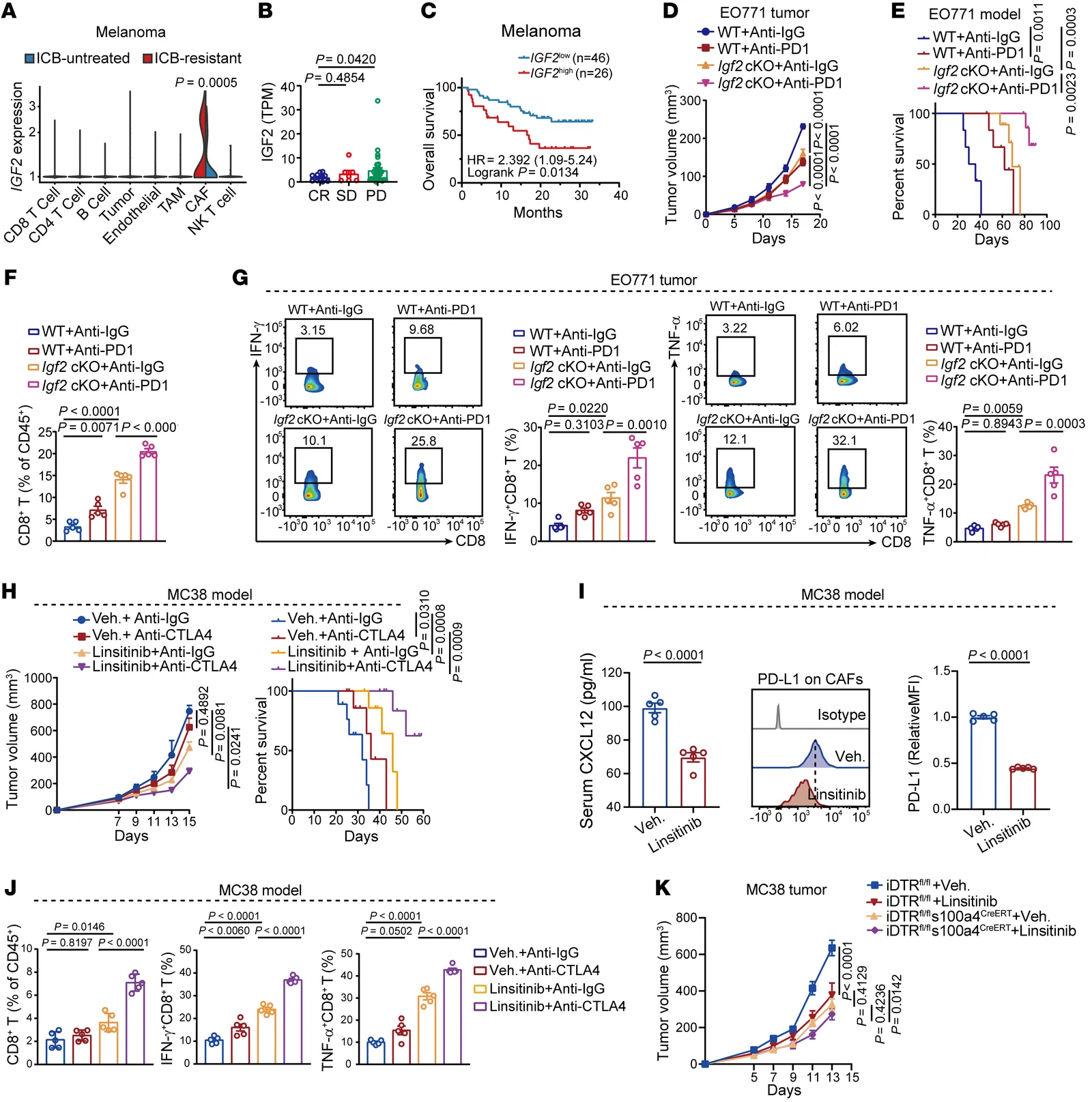
IGF2 blockade synergistically enhances the therapeutic efficacy of ICB. (**A**) IGF2 expression in the indicated cell clusters based on scRNA-Seq analysis of melanoma data (GSE115978) from the GEO database. (**B**) IGF2 expression in pretreatment tumors from patients with melanoma who had different treatment responses to anti–PD-1 (GSE115978). TPM, transcripts per million. (**C**) OS of patients with melanoma who received anti–PD-1 treatment (GSE115978) based on IGF2 expression in pretreatment tumors. (**D**–**G**) Tumor growth (**D**), mouse survival (**E**), percentage of CD8^+^ T cells (**F**), and percentage of IFN-γ^+^ or TNF-α^+^ CD8^+^ T cells (**G**) in EO771 tumors from WT or *Igf2*-cKO mice treated with anti-IgG or anti–PD-1 (10 mg/kg) (*n* = 5 mice per group). (**H**–**J**) Tumor growth and mouse survival (**H**), serum CXCL12 levels and expression of PD-L1 on CAFs (**I**), and percentage of CD8^+^ T cell abundance and IFN-γ^+^ and TNF-α^+^ CD8^+^ T cells (**J**) in MC38 tumor–bearing WT mice after treated with linsitinib (10 mg/kg), anti–CTLA-4 (5 mg/kg), or their combination in WT mice (*n* = 5 mice per group). (**K**) Growth of MC38 tumors in WT or iDTR^fl/fl^ *S100a4*^CreERT^ mice treated with vehicle or linsitinib (10 mg/kg) (*n* = 5 mice per group). Data are presented as the mean ± SEM (**B**, **F**, **G**, and **H**–**J**). *P* values were determined by 2-tailed, unpaired Student’s *t* test (**I**), 1-way ANOVA (**B**, **F**, **G**, and **J**), 2-way ANOVA (**A**, **D**, **H**, and **K**), or log-rank test (**C**, **E**, and **H**). Veh., vehicle.

The discovery that IGF2 blockade hinders immune evasion and tumor growth prompted an investigation into whether IGF2 deficiency could potentiate the antitumor response provoked by ICB. Initially, we analyzed ICB-related datasets using the Tumor Immune Syngeneic Mouse (TISMO) database. Tissue samples obtained from responders undergoing anti–CTLA-4 and anti–PD-1 therapy exhibited decreased levels of IGF2 expression within the mammary T11 tumor in comparison with the control group (Supplemental Figure 10A). Additionally, analysis of another scRNA-Seq dataset obtained from the Gene Expression Omnibus (GEO) database revealed heightened levels of IGF2 in CAFs, with this trend further exacerbated in ICB-resistant melanoma compared with ICB-untreated melanoma (Figure 6A). In patients with melanoma undergoing anti–PD-1 therapy, elevated levels of *IGF2* mRNA were observed in pretreatment tumors from nonresponders relative to levels in responders (Figure 6B). Among patients treated with anti–PD-1, those with tumors that had high IGF2 expression levels had notably reduced overall survival (OS) rates compared with those with tumors expressing low levels of IGF2 (Figure 6C). Subsequently, to assess whether IGF2 depletion in CAFs could enhance the antitumor activity of anti–PD-1 blockade, EO771 tumors were treated with either anti-IgG or anti–PD-1 antibodies in WT or *Igf2*-cKO mice. *Igf2*-cKO mice treated with anti–PD-1 demonstrated the most effective tumor retardation, significantly prolonged survival, and exhibited increased infiltration of T cells, along with enhanced antitumor activity of CD8^+^ T cells compared with WT mice treated with anti–PD-1 (Figure 6, D–G). Similarly, in the MC38 model, anti–CTLA-4 treatment in *Igf2*-cKO mice resulted in the most substantial therapeutic response compared with the other groups, accompanied by significantly increased infiltration and functional enhancement of CD8^+^ T cells (Supplemental Figure 10, B–D). Overall, the combined approach of IGF2 depletion and ICB significantly reshaped the antitumor immune microenvironment.

We next examined the combined efficacy of linsitinib and ICB in vivo. Linsitinib was administered either alone or in combination with anti–CTLA-4 to C57BL/6 mice bearing MC38 tumors. Linsitinib alone had a modest inhibitory effect on tumor growth, and the combined treatment of linsitinib with anti–CTLA-4 more effectively impeded tumor growth and extended survival. Notably, the combined therapy resulted in the complete eradication of tumors and achieved a state of tumor-free survival in 30% of the mice, an effect that was not seen in the other experimental groups (Figure 6H). Additionally, in line with our findings in *Igf2^–/–^* and *Igf2*-cKO mice, linsitinib treatment markedly reduced the levels of serum CXCL12 and PD-L1 on CAFs (Figure 6I). Furthermore, the quantities of total CD8^+^ T cells, along with IFN-γ^+^ or TNF-α^+^ CD8^+^ T cells, were substantially increased in tumors from the linsitinib-treated cohort compared with those from the control group. Crucially, the combination of linsitinib and anti–CTLA-4 further enhanced these patterns in the immune microenvironment (Figure 6J). The suppression of tumor growth by linsitinib (10 mg/kg) could not be attributed to the inhibition of the IGF1R on tumor cells, as linsitinib did not significantly add to the effect on tumor growth in *Igf2*-cKO mice (Supplemental Figure 10E). To confirm that fibroblast IGF1R is the primary target of linsitinib, we generated transgenic iDTR*^fl/fl^* *S100a4*^CreERT^ mice to specifically deplete fibroblasts. The results demonstrated a marked inhibitory effect on MC38 tumors treated with linsitinib in WT mice, similar to the effect seen with vehicle-treated MC38 tumors in iDTR*^fl/fl^* *S100a4*^CreERT^ mice. Nevertheless, linsitinib did not significantly contribute to the inhibition of MC38 tumor growth in iDTR*^fl/fl^ S100a4*^CreERT^ mice (Figure 6K). These findings suggest that the IGF1R on CAFs was the primary target of linsitinib. Additionally, in the 4T1 model that was resistant to ICB, treatment with linsitinib led to a substantial reduction in tumor size compared with the control group. Encouragingly, a combination of linsitinib and anti–PD-1 markedly improved the therapeutic response and reshaped the immune landscape, indicating that linsitinib might reverse ICB resistance (Supplemental Figure 10F). Overall, the loss of IGF2 or linsitinib synergistically improved the therapeutic efficacy of ICB.

### High IGF2 expression is correlated with a poor prognosis and a lower immunotherapy response in patients with cancer.

**Figure 7:**
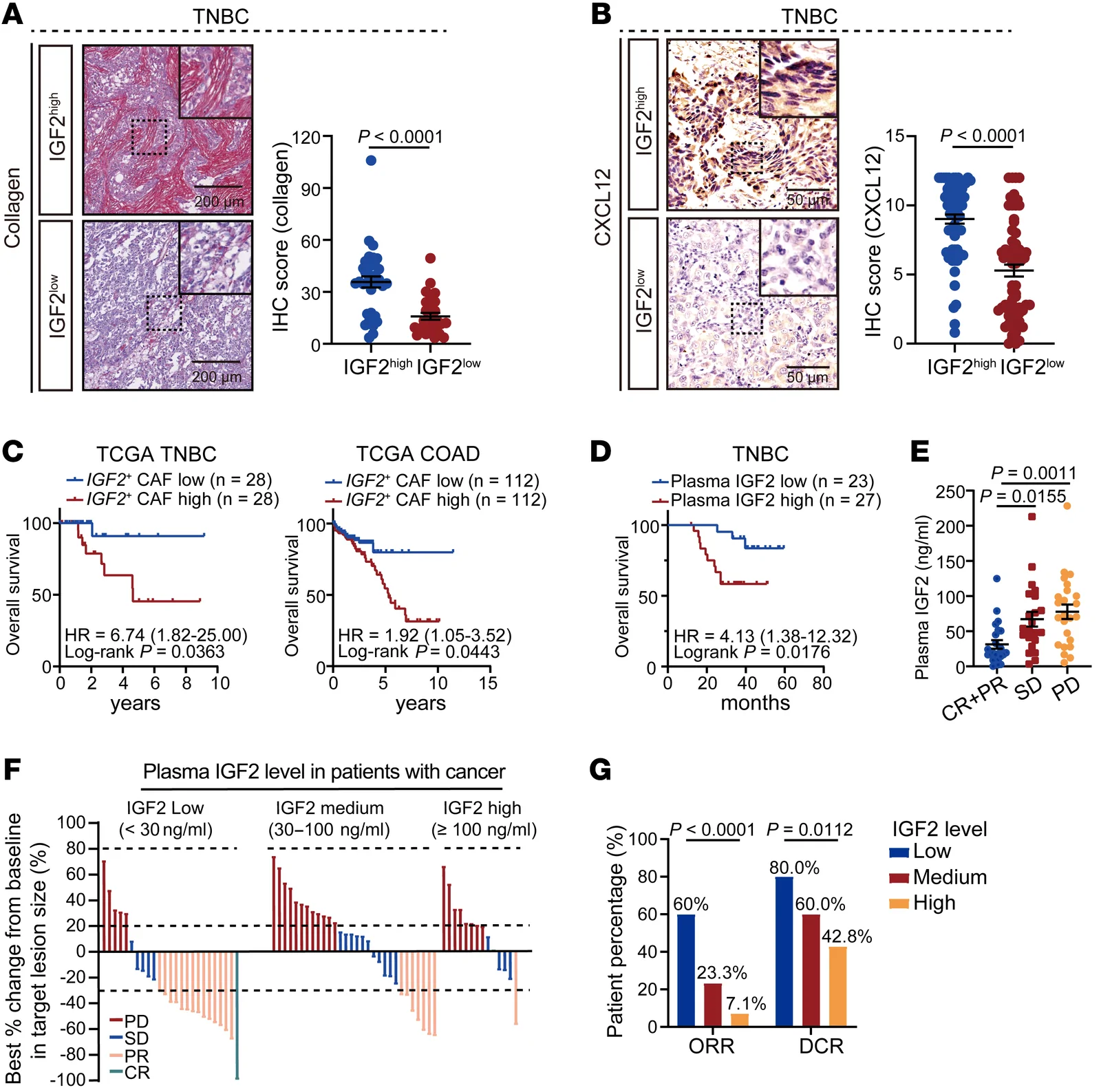
High levels of IGF2 are positively correlated with an unfavorable prognosis and resistance to immunotherapy in patients with cancer. (**A**) Analysis of collagen deposition by Picrosirius red staining in IGF2^hi^ (*n* = 35) and IGF2^lo^ (*n* = 30) human TNBC tissues. Scale bars: 200 μm. (**B**) IHC staining of CXCL12 in IGF2^hi^ (*n* = 70) and IGF2^lo^ (*n* = 67) human TNBC tumor tissues. Scale bars: 50 μm. Original magnification (insets), ×2 (**A**) and ×2.5 (**B**). (**C**) OS of patients with cancer with distinct infiltration levels of IGF2^+^ CAFs in TCGA cohort. (**D**) OS of patients with TNBC based on plasma IGF2 levels. (**E**) Plasma IGF2 levels in pretreatment blood collected from cancer patient groups with different responses to anti–PD-1 treatment. CR, 100% remission; PR, ≥30% remission; SD, <30% remission to <20% increase of tumor size; PD, ≥20% increase. (**F**) Waterfall plot depicting the responses to anti–PD-1 treatment in cancer patients with low levels (<30 ng/mL), medium levels (30–100 ng/mL), and high levels (>100 ng/mL) of plasma IGF2. (**G**) Assessment of the ORR and DCR among cancer patients with different plasma IGF2 levels (Fisher’s exact test). Data are presented as the mean ± SEM (**A**, **B**, and **E**). *P* values were determined by 2-tailed, unpaired Student’s *t* test (**A** and **B**) and 1-way ANOVA (**E**) and log-rank test (**C** and **D**).

Finally, we investigated a potential relationship between fibroblast IGF2 expression and clinical outcomes in patients with cancer. Consistent with our experimental findings (Supplemental Figure 7E), collagen-associated genes were expressed at higher levels in immune-excluded tumors than in immune-inflamed tumors, similar to IGF2 expression (Supplemental Figure 11, A and B). We observed increased collagen deposition in the IGF2^hi^ tissues from our TNBC cohort (Figure 7A). Furthermore, TCGA TNBC cohort also indicated that IGF2^hi^ tumors had elevated expression of collagen-associated genes (Supplemental Figure 11C). Additionally, we identified a positive correlation between the expression of IGF2/IGF1R and CXCL12 in the TNBC cohort from TCGA database (Supplemental Figure 11, D–G). We also observed higher CXCL12 expression in the IGF2^hi^ tissues compared with the IGF2^lo^ tissues within our TNBC cohort (Figure 7B). Together, these results provide additional evidences for the involvement of IGF2 in stimulating collagen deposition and CXCL12 expression within the TME. Moreover, cancer patients with a high infiltration level of IGF2^+^ fibroblasts had a poor prognosis (Figure 7C). In addition, patients with TNBC with high plasma IGF2 levels also had worse OS (Figure 7D).

We conducted an extensive investigation to elucidate the relationship between plasma IGF2 levels and patient responses to immunotherapies. Specifically, we analyzed pretreatment IGF2 levels in the plasma of a cohort consisting of 68 patients with cancer enrolled in a basket trial for anti–PD-1 treatment, encompassing individuals diagnosed with BRCA, COAD, and LUAD. Our findings unveiled a notable decrease in IGF2 levels in the plasma of patients who had a complete response (CR) or a partial response (PR) in contrast to those who had progressive disease (PD) (Figure 7E). Subsequently, given their plasma IGF2 levels, the patients with cancer were categorized into 3 groups. Notably, the group with lower plasma IGF2 levels demonstrated a notably higher overall response rate (ORR) and disease control rate (DCR) (ORR: 60% vs. 7.1%; DCR: 80.0% vs. 42.8%) when compared with patients with higher plasma IGF2 levels (Figure 7, F and G). Consistent with our findings from multiple mouse models, these clinical data further support the notion that IGF2 contributes to immune evasion and may serve as a prognostic indicator and therapeutic target for various cancers.

## Discussion

In this study, we uncovered the mechanism by which IGF2 reshapes the tumor immune microenvironment via modulation of CAFs. Our combined analysis of our own cohorts and public databases suggests that this phenomenon may be prevalent in numerous solid tumors, emphasizing the widespread nature and importance of this mechanism in tumor malignancy progression. CAFs comprise a diverse stromal cell population within the TME, differentiating into distinct myCAFs, iCAFs, and apCAFs, each with unique functions. While myCAFs are involved in tumor fibrosis and extracellular matrix remodeling, iCAFs foster an immune-suppressive microenvironment through the production of inflammatory mediators, and apCAFs are engaged in antigen presentation and immune modulation (11, 24). Furthermore, our findings revealed that IGF2 deficiency reduced the number of cells in each CAF cluster and inhibited CAF proliferation and collagen formation in the microenvironment, indicating a significant influence on the entire CAF population. Additionally, we observed that IGF2 induced the release of the key chemokine CXCL12, consistent with its high expression in iCAFs. However, the specific effect of IGF2 on CAF differentiation is still unclear, highlighting the need for further in-depth research in the future.

IGF2, a member of the insulin-like growth factor family, is a potent mitogen that exerts its protumor effects by binding to the IGF1R and activating downstream signaling cascades (2, 23). IGF2 regulates cell proliferation, differentiation, migration, and survival, thereby playing a crucial role in embryonic development and acting as a major growth hormone during pregnancy (25). Aberrant IGF2 expression is associated with diseases like breast, colon, and lung cancers (2). Although the role ofIGF2 in tumor cells is well studied, its effect within the TME, particularly in CAFs, remains underexplored (26). Our study reveals that CAFs serve as the primary source of IGF2 in the TME, consistent with findings from the previous study (27). Moreover, our study reveals a significant increase in TGF-β signaling in immune-excluded tumors, demonstrating its pivotal role in enhancing IGF2 expression in CAFs. TGF-β, a versatile cytokine, governs cell processes and immune responses. Notably, TGF-β plays a crucial role in promoting an immunosuppressive microenvironment by regulating immune cells and fostering Treg differentiation (28, 29). Additionally, TGF-β influences CAFs by stimulating their activation and fibrotic properties, prompting the release of bioactive molecules that contribute to tumor progression, metastasis, and immune evasion (30). Our findings highlight the role of TGF-β in upregulating IGF2 in CAFs, underscoring the cytokine’s vital involvement in CAF regulation and its diverse effect on shaping an immunosuppressive tumor microenvironment.

Of significant note, our discovery pinpoints IGF2 as a crucial upstream factor governing the immunosuppressive functions of CAFs, revealing it as a promising biomarker for T cell exclusion and responsiveness to immunotherapy interventions. Furthermore, our study demonstrates that the IGF1R inhibitor linsitinib, known for its reported inhibition of tumor growth in various cancers, did not augment the inhibitory effect on tumor growth in *Igf2*-cKO mice and mice with specific fibroblast depletion (iDTR^fl/fl^ *S100a4*^CreERT^ mice). This suggests that the primary target of linsitinib may be the IGF1R pathway in fibroblasts rather than in tumor cells. Clinical trials have indicated that it is challenging to achieve the anticipated therapeutic effects with IGF1R inhibitors (31–33). Given the regulatory role of IGF2 in fibroblast-mediated immunosuppression and our animal experiments showing the ability of linsitinib to enhance the efficacy of ICB, it is proposed that the combination of IGF1R inhibitors and ICB in clinical settings may yield more favorable therapeutic benefits for patients with cancer, especially those with high levels of IGF2 in tumor tissues or blood.

In tumor tissues, the generation of collagen primarily involves the activation of fibroblasts into CAFs by tumor cells, leading to increased collagen production. Additionally, tumor cells themselves can also contribute to collagen synthesis. The resulting excessive accumulation of collagen fibers in the TME has profound effects. Collagen deposition creates a stiffer, dense extracellular matrix, which can promote tumor progression, invasion, and metastasis. Moreover, the altered collagen composition can modulate the signaling cues and physical barriers, affecting immune cell infiltration and antitumor immune responses (12, 34–36). In our study, we found that IGF2 deficiency substantially inhibited collagen deposition in tumor tissues, indicating a critical role for IGF2 in modulating the formation of a CAF-mediated physical barrier. Nonetheless, the regulatory mechanisms underlying collagen production are intricate, warranting further investigation to elucidate the specific role of IGF2 in its generation.

CXCL12 is a chemokine with diverse effects in tumor biology. Within the TME, CXCL12 is expressed by various stromal cells, including CAFs and endothelial cells (19, 22, 37). CXCL12 influences tumor progression through interactions with its 2 receptors, CXCR4 and ACKR3, especially CXCR4. CXCR4 is expressed across various cell types, including cancer cells and immune cells (38). CXCL12-rich CAFs can impede CXCR4-expressing T cells from accessing tumor cells, hindering T cell infiltration. Our stRNA-Seq and scRNA-Seq analyses revealed that IGF2 deficiency in CAFs markedly obstructed the interaction between fibroblast-derived CXCL12 and CXCR4 on T cells. Furthermore, the interaction between fibroblast-derived CXCL12 and CXCR4 on tumor cells and monocytes also decreased notably following IGF2 loss. The tumor-specific interaction may enhance tumorigenesis and metastasis, possibly contributing to the protumor effect of IGF2 in CAFs in vivo. Moreover, stRNA-Seq analysis revealed a substantial increase in the expression of other chemokines and their receptors, specifically the antitumor chemokines CXCL9, CXCL10, and CXCL11, along with their receptors in IGF2^lo^ tumors. This observation suggests a transition of the TME toward an antitumor phenotype.

PD-L1, as a crucial immune checkpoint molecule, primarily interacts with its receptor PD-1, leading to T cell exhaustion, dysfunction, and anergy within the TME. The expression of PD-L1 is not confined to tumor cells but is also evident in a variety of immune cells, including macrophages, DCs, and MDSCs within the TME (4, 39, 40). Growing evidence has shown that CAFs also express PD-L1, contributing to tumor immunosuppression and unfavorable clinical outcomes (12, 41). Our study demonstrated that IGF2 notably enhanced PD-L1 expression CAFs, leading to the inhibition of T cell–mediated cytotoxicity. This underscored the considerable immunosuppressive effect of the PD-1/PD-L1 signaling. Additionally, we observed predominant PD-L1 expression on iCAFs, similar to IGF2 and CXCL12, suggesting the pivotal role of iCAFs in tumor immunosuppression. Furthermore, our clinical analysis uncovered a notable positive correlation between IGF2 and PD-L1 in tumor tissues. Considering the widespread expression of PD-L1 in the TME, this indicates that IGF2 released by CAFs may also upregulate the expression of PD-L1 on other cells, such as tumor cells, in addition to CAFs. Collectively, these results underscore the crucial role of CAF-derived IGF2 in controlling the immunosuppressive microenvironment and promoting tumor advancement.

## Methods

### Sex as a biological variable.

Our study examined male and female animals, and similar findings are reported for both sexes.

### Cell culture.

The murine mammary carcinoma cell lines EO771, EMT6, and 4T1, the colorectal carcinoma cell lines MC38 and CT26, the melanoma cell line B16-F10, the human mammary cell lines MDA-MB-231, MB468, and MCF-7, the human colorectal adenocarcinoma cell lines HCT116 and SW468, and the human melanoma cell line A375 were obtained from American Type Culture Collection (ATCC) and cultured according to ATCC guidelines. Mouse CAFs (mCAFs) and human CAFs (hCAFs) were isolated from the EO771 tumor and human breast cancer tissues, respectively. The cells were cultured at 37°C with 5% CO_2_, and the culture medium was refreshed daily.

### Animals.

Six- to 8-week-old mice on a C57BL/6 background with ablation of *Igf2* (*Igf2^–/–^*) were sourced from Cyagen Biosciences. The C57BL/6N-background *Igf2^fl/fl^* mice and C57BL/6-background iDTR^fl/fl^ mice were obtained from The Jackson Laboratory. *S100a4*^CreERT^ mice were procured from Shanghai Model Organisms Center, and WT C57BL/6 and BALB/c mice were acquired from Ensiweier. *Igf2^fl/fl^* and iDTR*^fl/fl^* mice were respectively crossed with *S100a4*^CreERT^ mice to generate *Igf2^fl/fl^* *S100a4*^CreERT^ and iDTR^fl/fl^ *S100a4*^CreERT^ mice. Before tumor cell inoculation, these mice received intragastric administration of tamoxifen (60 mg/kg) for 5 consecutive days. Following tamoxifen treatment, iDTR^fl/fl^ and iDTR^fl/fl^ *S100a4*^CreERT^ mice were intraperitoneally injected with diphtheria toxin (0.015 mg/kg) for 5 consecutive days. The mice were housed under controlled environmental conditions, with temperatures maintained at approximately 21°C–23°C, humidity levels between 40% and 60%, and a 12-hour light/12-hour dark cycle. All chemical reagents used are listed in Supplemental Table 1.

### Statistics.

Statistical analyses were conducted using GraphPad Prism (version 8.4.0) and R (version 4.3.2). Significance was calculated using an unpaired, 2-sided Student’s *t* test, unless otherwise specified. For comparisons involving more than 2 groups, data were analyzed using 1-way or 2-way ANOVA. Survival comparisons were assessed using the log-rank test. A *P* value of less than 0.05 was considered statistically significant, and exact *P* values are displayed in all graphs. The specific statistical methods applied are detailed in the figure legends.

### Study approval.

All experimental procedures and animal handling protocols were conducted in strict compliance with the *Guide for the Care and Use of Laboratory Animals* (National Academies Press, 2011) guidelines and were approved by the Research Ethics Committee of the First Affiliated Hospital of Chongqing Medical University (Chongqing, China). Ethics clearance for the use of pathological specimens and access to patient records was obtained from the Research Ethics Committees of the First Affiliated Hospital of Chongqing Medical University (2017-P2-141-01 and 2022-K121). All participants and/or their legal representatives provided written informed consent as stipulated.

### Data availability.

The data produced in this study are accessible in the article and its supplemental materials. Values for all data points in graphs are reported in the Supporting Data Values file. The raw sequencing data reported in this work have been deposited in the Genome Sequence Archive (GSA) of the National Genomics Data Center, China National Center for Bioinformation/Beijing Institute of Genomics, Chinese Academy of Sciences (GSA: CRA018666; GSA human: HRA008475) and are publicly accessible at https://ngdc.cncb.ac.cn/gsa/browse/CRA018666 and https://ngdc.cncb.ac.cn/gsa

## Author contributions

DS, YW, J Li, J Liu, JW, GR, and HL designed the study. DS, YW, J Li, ZY, XW, JS, LL, QW, YC, HF, and TL conducted experiments and/or analyzed data. HD, JH, WP, YW, FL, QL, LZ, and YZ provided critical reagents and/or samples. DS and YW analyzed the clinical data. DS, YW, JW, GR, and HL wrote and edited the manuscript. HL supervised the study.

## Supplementary Material

Supplemental data

Unedited blot and gel images

Supporting data values

## Version 1 11/15/2024

Electronic publication

## Version 2 09/29/2026

Corrected figure 2D and associated supporting data values due to error in the source data.
