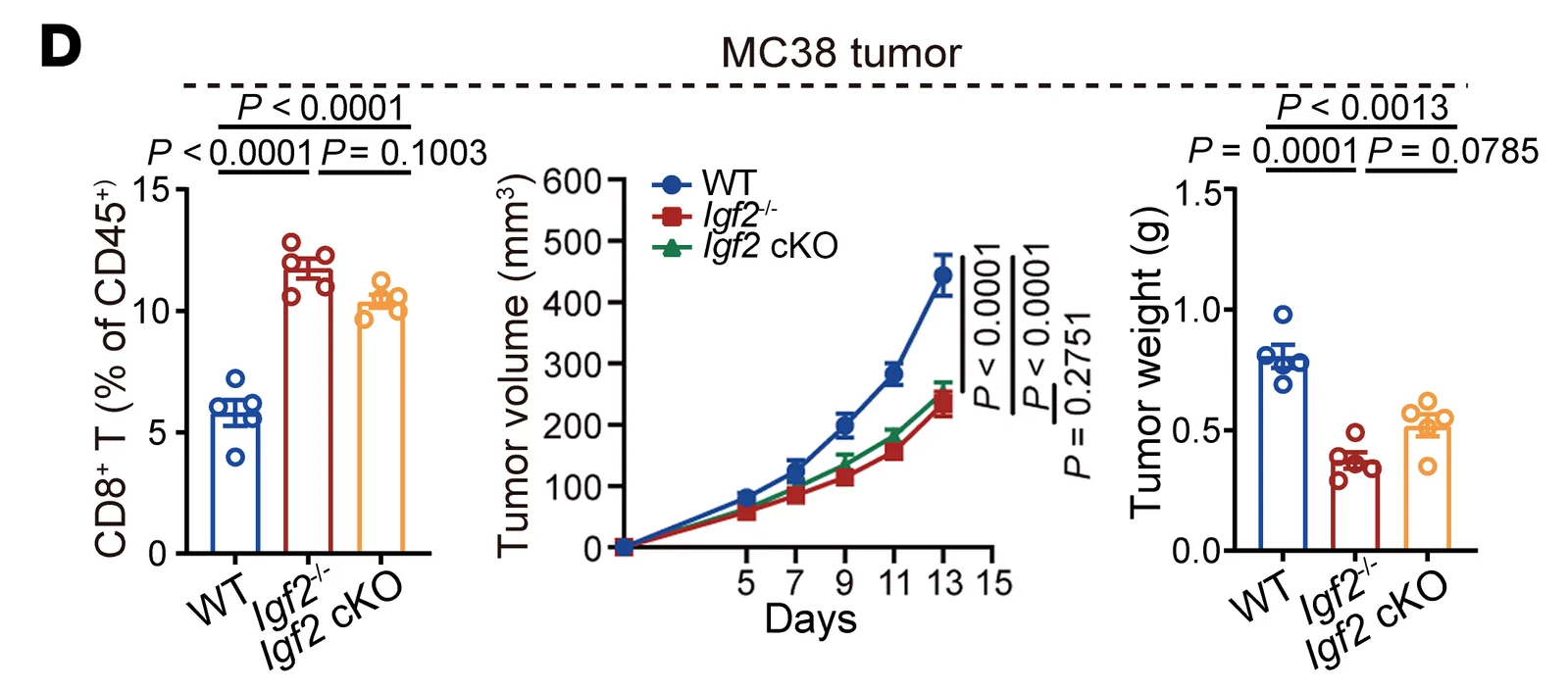# Corrigendum to Insulin-like growth factor 2 drives fibroblast-mediated tumor immunoevasion and confers resistance to immunotherapy

**DOI:** 10.1172/JCI213105

**Published:** 2026-10-01

**Authors:** Daqiang Song, Yushen Wu, Jie Li, Jiazhou Liu, Ziying Yi, Xiaoyu Wang, Jiazheng Sun, Liuying Li, Qianxue Wu, Yuru Chen, Huiying Fang, Tiankuo Luan, Huimin Du, Jing Huang, Weiyan Peng, Yuxian Wei, Fan Li, Qin Li, Li Zhang, Yong Zhu, Jingyuan Wan, Guosheng Ren, Hongzhong Li

Original citation: *J Clin Invest*. 2024;134(22):e183366. https://doi.org/10.1172/JCI183366

Citation for this corrigendum: *J Clin Invest*. 2026;136(19):e213105. https://doi.org/10.1172/JCI213105

The authors recently alerted the Editors to errors in the source data underlying the tumor growth panel in Figure 2D and the associated supporting data values file that were identified in response to an institutional mandate for a post-publication audit of the data. The correct Figure 2D is shown below. The HTML and PDF versions of the article as well as the accompanying Supporting Data Values file have been updated.

The authors regret the errors.

## Figures and Tables

**Figure 2:**