# Supplementary material for: Insulin-like growth factor 2 drives fibroblast-mediated tumor immunoevasion and confers resistance to immunotherapy
Source: J Clin Invest. 2024 Nov 15;134(22):e183366. doi: 10.1172/JCI183366 (PMC11563680; doi:10.1172/JCI183366)
Supplement: Supplemental data [file jci-134-183366-s270.pdf]

Insulin-like growth factor 2 drives fibroblast-mediated tumor immunoevasion and confers resistance to immunotherapy

Daqiang Song[1,2#], Yushen Wu[1,3#], Jie Li[1#], Jiazhou Liu[1,5#], Ziying Yi[1], Xiaoyu Wang[1], Jiazheng Sun[1], Liuying Li[1], Qianxue Wu[1], Yuru Chen[1], Huiying Fang[1], Tiankuo Luan[1], Huimin Du[3], Jing Huang[4], Weiyan Peng[1], Yuxian Wei[5], Fan Li[5], Qin Li[6], Li Zhang[7], Yong Zhu[8], Jingyuan Wan[2*], Guosheng Ren[1,5*], Hongzhong Li[1*]

Supplemental Figures 1-11

Supplemental Methods

Supplemental Table 1

Figure S1. IGF2 is highly expressed in immune-excluded tumors, related to Figure 1.

(A) Representative IHC images showing expression of CD3 in the immune-inflamed or immune-excluded human tumors. (**B)** Scatter plot of 9-quadrant association analyses of transcriptomic levels from log2-fold change (Log2 FC) in both TCGA COAD cohort and our COAD cohort. (**C)** Expression of differentially expressed genes in the tumors across 9 human cancers based on immune exclusion score of TIDE system. (**D)** The dot plot illustrating the differential enrichment of stromal cells in IGF2-high tumor tissues compared to those in IGF2-low tissues. The red plot representing up-regulated enrichment, the blue plot representing down-regulated enrichment, and the orange plot indicating no significant enrichment. The node size represents the *P* value by the Mann-Whitney U-test (D). *R* value is from Pearson's correlation coefficient (B).

Figure S2. IGF2 is primarily expressed in CAFs, related to Figure 1.

(A) Representative IHC images illustrating the expression of IGF2 and CD3 in the immune-inflamed or immune-excluded human TNBC tissues. (**B)** Correlation between IGF2 and CAFs infiltration in human BRCA and COAD based on TIMER system. (**C)** UMAP showing IGF2 expression on the cell clusters in the TME of human COAD (GSE179784). (**D)** IGF2 expression on the cell clusters from human LUAD tumor and adjacent tissues (GSE123904). (**E**) Flow cytometry analysis showing the IGF2 expression in the CAFs, $CD45^+$ immune cells, and malignant cells in the TME of EO771 or MC38 tumors (n = 5 mice per group). (**F**) Expression of IGF2 in human or mouse CAFs and tumor cell lines was detected by western blot. (**G**) GO analysis showing the enriched biological processes in the immune excluded tumor compared to immune inflamed tumors. (**H**) The expression of TGFB1 in the immune-inflamed or immune-excluded TNBC and COAD tumors. (**I**) The correlation between the expression of IGF2 and TGFB1 in the TCGA BRCA and COAD cohorts. (**J**) GSEA analysis from scRNA-seq or bulk RNA-seq data showing the enriched TGF-β signaling in the WT CAFs compared to the $Igf2^{-/-}$ CAFs. (**K-M**) Western blot (K) or flow cytometry analysis (L and M) showing the expression of IGF2 in the hCAFs or mCAFs treated with rTGF-β1 (10 ng/ml) for different time points or exposed with rTGF-β1 for 24 h in the indicated concentrations. *P* values are from an unpaired Student's t-test (D and H) and one-way ANOVA (E, L, and M). *R* value is from Pearson's correlation coefficient (B and I).

Figure S3. IGF2 deficiency does not impact mouse T cell homeostasis, related to Figure 2.

(A) Genotyping to verify knockout of IGF2 in the mice. (**B**) IF analysis showing the expression of IGF2 in the CAFs within tumors from *Igf2*$^{-/-}$ and *Igf2* cKO mice. Scale bar, 100 μm. (**C**) Western blot analysis showing the expression of IGF2 in the CAFs from EO771 tumors in WT, *Igf2* cKO, and *Igf2*$^{-/-}$ mice. (**D**) Percentage of total or CD44^{high} CD62L^{low} CD4^{+} and CD8^{+} T cells from WT, *Igf2*$^{-/-}$, and *Igf2* cKO mice (n = 5 mice per group). PLNs, peripheral lymph nodes; MLNs, mesenteric lymph nodes. (**E**) Migration changes of T cells cocultured with WT or *Igf2*$^{-/-}$ CAFs treated with linsitinib (5 μM) or recombinant mouse IGF2 protein (10 μM). **(F)** Expression level of IGF2 in shNC or sh*IGF2* human CAFs. **(G)** Migration changes of T cells cocultured with shNC or sh*IGF2* human CAFs treated with linsitinib (5 μM) or recombinant human IGF2 protein (10 μM). (**H**) The gating strategy of flow cytometry for TME analysis in this study. Data are presented as mean ± s.e.m (D, E, and G). *P* values are from a one-way ANOVA (E and G) and two-way ANOVA (D).

Figure S4. Inhibition of IGF2 in CAFs promotes T cell infiltration, related to Figure 2.

(A) Genotyping to verify conditional knockout of IGF2 in the mice. (**B)** Percentage of infiltrating $CD8^+$ T cells and tumor growth in the B16-F10 tumor-bearing WT or *Igf2* cKO mice (n = 5 mice per group). (**C)** Tumor growth of MC38 tumors with indicated treatment. $CD8^+$ T cells were depleted by anti-CD8α antibody (10 mg/kg) in WT or $Igf2^{-/-}$ mice (n = 10 mice per group). (**D)** Comparison of $CD8^+$ T cell migration, proliferation (CFSE dilution), and cytotoxic/cytolytic activities (IFN-γ^+ and TNF-α^+) between WT and $Igf2^{-/-}$ $CD8^+$ T cells was analyzed by flow cytometry (n = 3). (**E** and **F)** Percentage of infiltrating $CD8^+$ T cells and tumor growth in 4T1 model (E) and CT26 model (F) (n = 5 mice per group). (**G)** Tumor growth of 4T1 tumors with indicated treatment. $CD8^+$ T cells were depleted by anti-CD8 antibody (10 mg/kg) in BALB/c mice (n = 6-7 mice per group). Data are presented as mean ± s.e.m (B, C, D, E, and G). *P* values are from the two-way ANOVA (B, C, E-G) and two-tailed unpaired Student's t-test (B, D-F).

Figure S5. IGF2 loss significantly enhances T cell function and remodels the TME, related to Figure 2.

(A) The gating strategy of flow cytometry for T cell function analysis. (**B**) Percentage of IFN-γ^+ and TNF-α^+ $CD8^+$ T cells in the TME of B16-F10 tumor-bearing WT or *Igf2* cKO mice (n = 5 mice per group). (**C)** Chord diagram showing the correlation between IGF2 and markers of $CD8^+$ cytotoxic T lymphocyte (CTL) in the TNBC cohort from TCGA database. (**D)** t-Distributed Stochastic Neighbor Embedding (tSNE) showing a plot of tumor-infiltrating lymphocytes (TILs) within EO771 tumors, with color-coded clusters overlaid for visual differentiation. (**E)** The ratio of immune cells in the TME of EO771, MC38, and B16-F10 tumors in the WT or *Igf2* cKO mice was analyzed by flow cytometry (n = 5 mice per group). Data are presented as mean ± s.e.m. (B). *P* values are two-tailed unpaired Student's t-test (B and E). Pearson's correlation coefficient is calculated (C). $^{*}P < 0.05$, $^{**}P < 0.01$.

Figure S6. Marker expressions of cell clusters in the EO771 tumors based on scRNA-seq, related to Figure 3.

(A) Lineage-defining genes in cell clusters based on scRNA-seq analysis of EO771 tumors. (**B)** Volcano plots showing the differentially expressed genes in the cell clusters in EO771 tumors. (**C)** Percentage of cell clusters in the EO771 tumors from WT or *Igf2* cKO mice. (**D**) The expression of fibroblast cluster markers. (**E**) Heatmap showing the expression of indicated genes in the fibroblast cluster from EO771 tumors. (**F)** Trajectory analysis of fibroblast cell types. **(G)** The density of cell types of fibroblast subsets at different times. (**H**) The expression of IGF2 in the fibroblast subsets in EO771 tumors. *P* values are one-way ANOVA (B and H).

Figure S7. IGF2 enhances CAFs proliferation and stimulates CAFs to release CXCL12 and express PD-L1, related to Figure 4.

(A) Enrichment of fibroblasts in the TME of IGF2-high or IGF2-low TNBC and COAD tissues from the TCGA database. (**B** and **C)** Proliferation of WT or *Igf2*$^{-/-}$ CAFs (B) and shNC or sh*IGF2* CAFs (C) treated with linsitinib (5 μM) or recombinant IGF2 protein (10 μM). (**D)** Enrichment plot for collagen formation signaling by GSEA in RNA-seq data from WT CAFs versus *Igf2*$^{-/-}$ CAFs. (**E)** Analysis of collagen deposition by picrosirius red staining in the MC38 tumors from WT, *Igf2*$^{-/-}$, *Igf2* cKO mice. Scale bar, 500 μm. (**F**) Heatmap showing the differentially expressed genes in the shNC and sh*Igf2* CAFs (n = 3). (**G)** The expressions of CXCL12 and PD-L1 on the shNC or sh*IGF2* CAFs treated with linsitinib (5 μM) or recombinant IGF2 protein (10 μM) were analyzed by flow cytometry (n = 3). Data are presented as mean ± s.e.m (A, B, and G). *P* values are from a two-tailed unpaired Student's t-test (A) and one-way ANOVA (B, C, and G).

Figure S8. Spatial transcriptomic analysis reveals that the interaction between CAFs and T cells is mediated by CXCL12 and PD-L1 signaling, related to Figure 4.

(A) PD-L1 signaling among cell clusters in spatial locations in IGF2-high or IGF2-low COAD tumors. The line thickness denotes the strength of the interactions. (**B)** Interaction between ligands and their receptors in the CXCL signaling in IGF2-high or IGF2-low COAD tumors.

Figure S9. Inhibiting the IGF2 pathway in CAFs markedly suppresses CAFs proliferation and enhances the infiltration and function of T cells, related to Figure 5.

(A) Enrichment of signaling pathways in CAFs based on the stRNA-seq analysis. (**B)** Expression level of phosphorylation of Akt protein in the BRCA and COAD from TCPA database. (**C** and **D)** Activation of Akt signaling pathway in the shNC or shIGF2 human (C) or mouse (D) CAFs was assessed by western blot. (**E**) Proliferation of WT or *Igf2*$^{-/-}$ CAFs treated with MK2206 (10 μM) or SC79 (10 μM) (n = 3). **(F)** GSEA analysis showing the relationship between IGF2, IGF1R, CXCL12, and the PI3K/Akt signaling pathway in the BRCA cohort from the TCGA database. (**G)** Expression of key proteins in the Akt signaling pathway in the shNC or sh*IGF1R* human CAFs was assessed by western blot. (**H)** Proliferation of shNC or sh*IGF1R* human CAFs. (**I)** Percentage of TNF-α^+ CD8$^+$ T cells cocultured with shNC or sh*IGF1R* CAFs (n = 3). (**J** and **K)** Percentage of infiltrating CD8$^+$ T cells (J), percentage of IFN-γ^+ and TNF-α^+ CD8$^+$ T cells (K) in the indicated groups (n = 5). (**L)** Tumor growth of 4T1 tumors in the indicated groups (n = 5 mice per group). Data are presented as mean ± s.e.m. (B, E, H-L). *P* values are from a two-tailed unpaired Student's t-test (B, J and K) and one-way ANOVA (E, H, and I), and two-way ANOVA (L).

Figure S10. Blockade of IGF2/IGF1R axis significantly enhances the therapeutic efficacy of ICB, related to Figure 6.

(A) IGF2 expression in the mammary T11 tumors treated with anti-PD1 and anti-CTLA4. **(B-D)** Tumor growth and mice survival (B), percentage of infiltrating $CD8^+$ T cells (C), percentage of IFN-γ^+ and TNF-α^+ $CD8^+$ T cells (D) in the MC38 tumor-bearing WT or *Igf2* cKO mice treated with anti-IgG or anti-CTLA4 antibodies (5 mg/kg) (n = 5 mice per group). **(E)** Tumor growth of MC38 tumors in the WT and *Igf2* cKO mice treated with vehicle or linsitinib (10 mg/kg) (n = 5 mice per group). **(F)** Tumor growth and percentage of immune cells in TME of 4T1 tumors in the indicated groups (n = 5 mice per group). Data are presented as mean ± s.e.m. (A-F). *P* values are from a two-tailed unpaired Student's t-test (A) and one-way ANOVA (C and D) and two-way ANOVA (B, E, and F) and log-rank test (B).

Figure S11. IGF2/IGF1R axis positively correlates with CXCL12, CD274, and collagen-associated genes, related to Figure 7.

(A and **B)** Heatmap showing the expressions of indicated genes in both human TNBC (A) and COAD (B). (**C)** Co-expression analysis of collagen-associated genes in the IGF2-high or IGF2-low TNBC from the TCGA database. (**D-G)** Correlation between CXCL12 and IGF2 (D and E) or IGF1R (F and G) in BRCA or COAD from TCGA database. R value is from Pearson's correlation coefficient (D-G).

SUPPLEMENTAL METHODS

RNA sequencing and data analysis

Total RNA was extracted from WT and *Igf2*$^{-/-}$ CAFs, shNC and sh*IGF*2 CAFs, TNBC and COAD tumor tissues using Trizol reagent (15596026, Invitrogen). After dispatching RNA samples to ANNOROAD for library preparation and sequencing, the raw fastq files from RNA sequencing on NovaSeq (Illumina) were employed to generate gene read counts via htseq, enabling the subsequent computation of transcripts per million (TPM). Differentially expressed genes were identified using the R package DESeq2, applying filtering parameters including fold change above 2, adjusted $P < 0.05$, and an average log2 (TPM) in the high expression group exceeding 0.

Human samples

Tumor tissues and blood plasma samples were procured from patients diagnosed with TNBC, with additional tumor specimens collected from individuals with COAD. Blood plasma samples were obtained from patients diagnosed with advanced lung (47 samples), colon (12 samples), and breast (10 samples) cancers prior to receiving anti-PD-1 treatment with Camrelizumab from December 24, 2019 to February 27, 2021. Evaluation of treatment response was conducted in adherence to the Response Evaluation Criteria in Solid Tumors (RECIST version 1.1), focusing on the percentage change in target lesion diameter utilizing pre-treatment and treatment imaging scans.

Cell isolation

Tumor specimens were mechanically dissociated using scissors and subsequently underwent enzymatic digestion with Liberase (590706, 2 mg/mL, Roche). The resulting

mixture was filtered through 70-μm filters (bs-70-xbs, Biosharp) to generate single-cell suspensions. After lysis of red blood cells, the cells were washed and then resuspended in a flow cytometry buffer for subsequent analyses. For CAFs isolation, the methodology for this study was adapted from a previous publication (1). Tumor samples were mechanically dissociated and enzymatically digested with Liberase (590706, 2 mg/mL, Roche) in DMEM for 45 minutes at 37°C. Single-cell suspensions were obtained by passing the digested tissues through 70-μm filters. The cells were cultured in DMEM supplemented with 10% FBS (04-001-1Acs, BI) and 1% penicillin-streptomycin (15140122, Gibco). CAFs were identified as cells that adhered to the culture dish within 15 minutes, while non-adherent cells were discarded.

Single-cell RNA sequencing

Single-cell suspensions were obtained from EO771 tumors in WT or *Igf2* cKO mice. A composite sample was generated by randomly pooling three tumor specimens from the identical cohort, which was subsequently dispatched to ANNOROAD (Beijing, China) for sequencing analysis. The cells were counted and loaded onto the 10X device from 10X Genomics. As per the manufacturer's guidelines, the samples were processed and sequenced on an Illumina NovaSeq 6000 platform by Illumina, Inc. located in San Diego, CA. The Cell Ranger analysis pipeline (v1.2) was employed to perform sample demultiplexing, barcode processing, alignment, filtering, unique molecular identifier counting, and aggregation of sequencing runs. Subsequent analyses were performed in R using the Seurat package (version 5.0.1). Cells exhibiting high expression of multiple cell population-specific marker genes, indicating potential doublets or multiplets, were

removed based on gene expression profiles. Additionally, cells with less than 200 genes or over 7,000 genes detected, and those with mitochondrial transcripts comprising over 10% of the total library, were excluded from further analysis. The function "Find Variable Features" was utilized to identify genes with high variability. Principal Component Analysis was conducted using the top 2,000 variable genes. Uniform Manifold Approximation and Projection (UMAP) was employed for dimensionality reduction to visualize inferred cell clusters, leveraging the top 15 principal components. To categorize immune cell populations, a Wilcoxon rank-sum test was performed for a differential expression analysis between each cluster and all other cells. Following this, the top differential expression outcomes for each cluster were compared against canonical markers representing a diverse set of immune cell populations. This led to the identification of a consensus panel of transcriptional markers for each of the 10 clusters.

Spatial transcriptomics data analysis

The stRNA-seq slides contained two identical capture areas from two human COAD samples, one with high IGF2 expression and the other with low IGF2 expression. Gene expression data from the spatial transcriptomics (ST) slides was captured employing the Visium Spatial platform by 10x Genomics, utilizing spatially barcoded mRNA-binding oligonucleotides as per the default protocol. Raw sequencing reads from the stRNA-seq were subjected to quality checks and mapped utilizing Space Ranger (version 1.1). The resulting gene-spot matrices obtained post-processing of ST data from both ST and Visium samples were analyzed using the Seurat package (version

5.0.1) in R. Spots were filtered for a minimum detected gene count of 200 genes, and genes with fewer than 10 read counts or expressed in fewer than 3 spots were excluded. Normalization across spots was carried out with the LogVMR function. Dimensionality reduction and clustering were conducted using independent component analysis (PCA) with a resolution of 0.8 and the first 30 principal components. Spatial feature expression plots were created using the SpatialFeaturePlot function in Seurat. The library was prepared, and subsequent sequencing was executed on a NovaSeq 6000 platform.

Bioinformatics

The tumor immune microenvironment in human TNBC and COAD was classified according to previously established methods (2). In brief, the immune-inflamed type was classified as brisk diffuse, and the immune-excluded type was classified as brisk band-like. In our RNA-sequencing study of TNBC and COAD samples, we identified two subtypes: immune inflamed and immune excluded. We used TPM data and the limma R package to analyze differential gene expression, resulting in the identification of upregulated genes in immune excluded TNBC (TNBC DEGs) and COAD (COAD DEGs) samples. A Venn diagram was then used to compare and identify overlapping DEGs between the two cancer subtypes. Moreover, we employed the "clusterProfiler" package in R to perform Kyoto Encyclopedia of Genes and Genomes (KEGG) enrichment analysis, aiming to identify relevant signaling pathways associated with IGF2. The assessment of stromal cell enrichment in the TME was conducted using the CIBERSORT and MCPcounter algorithms. For GSEA, version 4.0.3 of the GSEA software was utilized, while analysis of scRNA-seq data was carried out using the

Seurat R package. Terms with statistical significance ($P < 0.05$) and at least 3 enriched genes were considered in the analysis.

Tumor models

Following the administration of tamoxifen, MC38 and CT26 cells were subcutaneously injected at a concentration of 1.0×10^5 cells, while B16-F10 cells were injected at a concentration of 1.5×10^5 cells into the right hind leg region of the mice. In the mammary tumor model, 4T1 and EO771 cells were transplanted into the mammary fat pad of various mouse strains, including wild-type Balb/c, $iDTR^{flox/flox}$, $iDTR^{flox/flox}$*S100a4*^{CreERT}, *Igf2*$^{-/-}$, *Igf2*$^{flox/flox}$, or *Igf2*$^{flox/flox}$*S100a4*^{CreERT} C57BL/6 mice. Tumor size was calculated using the formula: volume (mm^3) = (long axis) × (short axis)^2/2. Tumor volume was monitored starting from day 5 post-tumor inoculation and then every two days. In certain experiments, tumor-bearing mice were euthanized on day 20, and the tumors were harvested for flow cytometry analysis. In combinatory experiments, tumor monitoring commenced on day 5 post-tumor inoculation and continued every two days. Survival time of mice was recorded by monitoring tumor volumes, and mice were euthanized if the tumor volume exceeded 2000 mm^3 in accordance with ethical guidelines for experimental animal use. Linsitinib (S1091, 10 mg/kg/day, Selleck) was administered orally for five consecutive days, beginning on day 5 post-tumor inoculation. The anti-PD-1 antibody (10 mg/kg) and anti-CTLA-4 antibody (5 mg/kg) were intraperitoneally injected on days 5, 7, 9, 11, and 13 following tumor inoculation. In the T cell depletion experiments, mice received anti-CD8

antibody (10 mg/kg) every 3 days, starting 3 days prior to MC38 or 4T1 tumor inoculation. The depletion of $CD8^+$ T cells was confirmed through flow cytometry.

Flow cytometry

Live cells were distinguished using Fixable Viability Dye eFluor 450. In order to block Fc receptors, cells were pre-incubated with purified anti-CD16/32 antibody. After washing, cells were incubated with a cocktail of primary antibodies against cell surface markers. Intracellular staining was carried out by fixing and permeabilizing cells using the Foxp3/Transcription Factor Staining Buffer Set, followed by staining with fluorochrome-conjugated antibodies targeting FOXP3. For cytokine staining, cells underwent stimulation with Cell Stimulation Cocktail, followed by staining with anti-IFN-γ and anti-TNF-α. Stained cells were analyzed using a BD FACSCanto II Flow Cytometer with BD FACSDiva software (BD Biosciences), and the data were processed with FlowJo software (version 10.5.3).

Mass cytometry

As described previously, live single cells were isolated from tumor tissues for subsequent mass cytometry (CyTOF) analysis (3). Cell viability was evaluated through staining with cisplatin (25 µM) for 1 minute, followed by labeling with a metal-tagged monoclonal antibody cocktail targeting cell surface molecule. Subsequent steps involved treating cells with Fixation/Permeabilization Buffer and staining with monoclonal antibody cocktails against intracellular proteins. Analysis was conducted utilizing the CyTOF 2 instrument at the Institute of Liver Diseases (Beijing You-an Hospital Affiliated with Capital University of Medical Sciences). The resultant CyTOF

files underwent normalization and manual gating in Cytobank software (version 9.1). Prior to Phenograph clustering analysis utilizing the R cytofkit package (version 0.99.0) on pooled samples to delineate immune subsets, data transformation was executed utilizing the cytofAsinh function. Heatmaps were generated based on the mean value of each marker in clusters, with cell frequency in each cluster determined by the ratio of assigned cell events to total $CD45^+$ cell events in the corresponding sample. Fluidigm antibodies were used for the mass cytometry analysis. All antibodies were presented in the Supplemental Table 1.

Immunohistochemistry (IHC)and immunofluorescence (IF) staining

Standard IHC and IF staining were performed according to established protocols as previously described (4). For IHC staining, the primary antibodies employed comprised anti-CD3, anti-IGF2, and anti-SDF. In IF staining, anti-α-SMA (ab7817) and anti-IGF2 antibodies were utilized. Secondary antibodies conjugated with DyLight 488 or DyLight 594 specific to rabbit or mouse IgG were sourced from Thermo Fisher Scientific.

T cell migration assay

The assays for stimulation of human and mouse T cells were previously described (5, 6). Human peripheral blood mononuclear cells (PBMCs) were stimulated with a suboptimal dose of immobilized anti-CD3/CD28 antibody. Mouse T lymphocytes were isolated from the spleens of 8-week-old C57BL/6J mice and similarly stimulated with anti-CD3/CD28 antibody. In the T cell migration assay, transwells featuring 8 μm pore diameters were employed, whereby activated splenic T cells were seeded within the

upper chamber. In the bottom chamber, mouse or human tumor cells were cultured, along with mouse or human cancer-associated fibroblasts (CAFs) treated with or without linsitinib (5 µM), recombinant mouse IGF2 protein (10 µM) or human IGF2 protein (10 µM), MK2206 (10 µM), or SC79 (10 µM), added 0.5 h before T cell addition to assess CAFs' impact on T cell migration. Following 6 h of migration, cells from the lower chamber were harvested, labeled with anti-CD8 antibodies, and quantified through flow cytometry analysis.

ELISA

IGF2 and CXCL12 levels in the blood plasma were measured using ELISA kits specific for IGF2 and CXCL12, respectively, following the instructions provided by the manufacturer.

Western blotting

Western blotting was carried out following the procedure described previously (7). The primary antibodies utilized in this study were IGF2, IGF1R, AKT, p-AKT, GAPDH and β-Actin.

Gene silencing by lentiviral transduction

Human *IGF2*- and *IGF1R*-targeting shRNAs for lentiviral production were supplied by Genecopoeia Company. Utilizing the Polyjet DNA in vitro Transfection Reagent, the lentiviral expression vector was co-transfected alongside the lentivirus packaging vectors into 293T cells. Subsequently, CAFs were stably transfected with viral particles for 48-72 h.

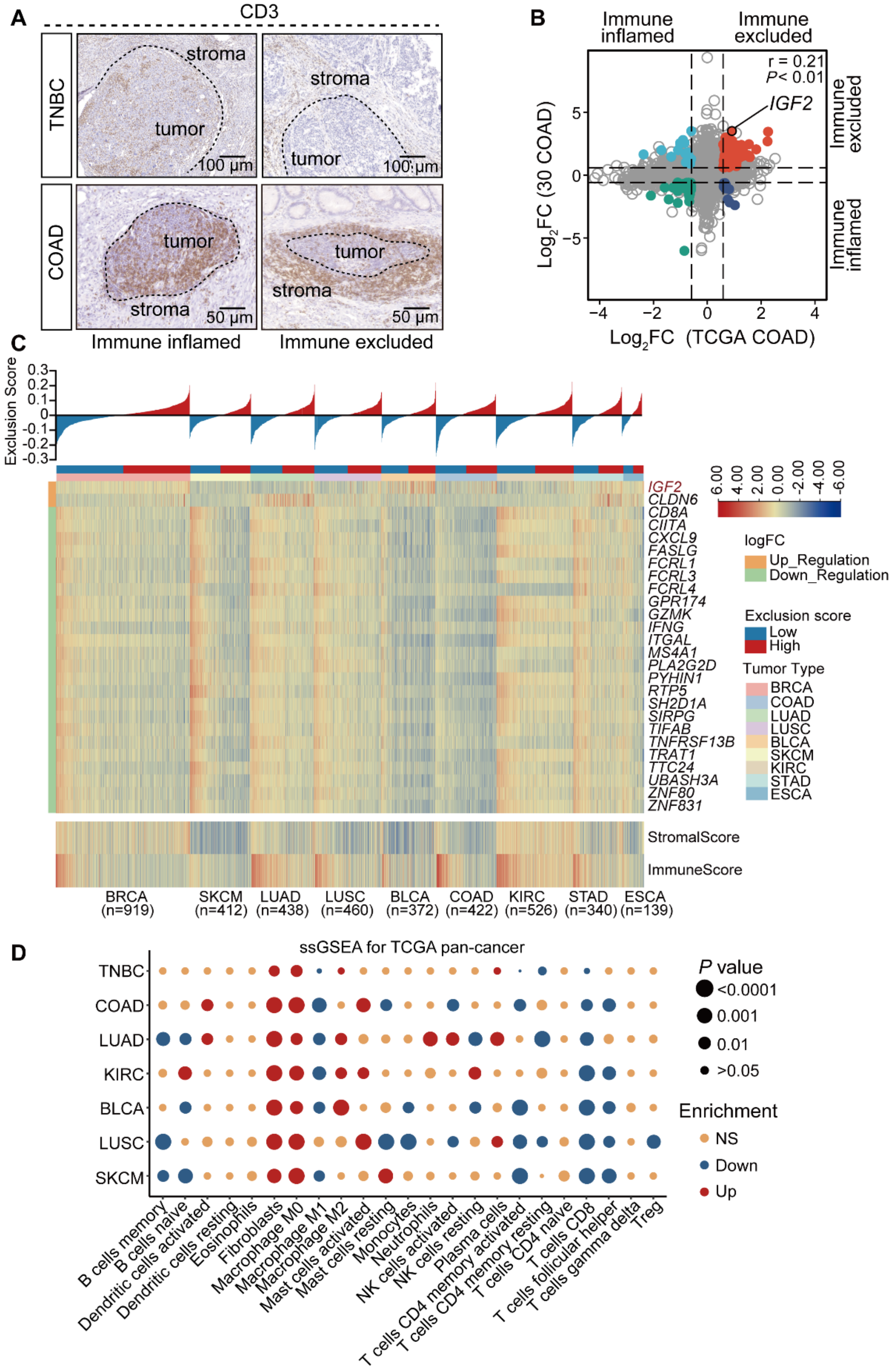
A
CD3
TNBC
COAD
stroma
tumor
100 μm
50 μm
Immune inflamed
Immune excluded
B
Immune inflamed
Immune excluded
r = 0.21
P< 0.01
IGF2
Log2FC (30 COAD)
Log2FC (TCGA COAD)
C
Exclusion Score
IGF2
CLDN6
CD8A
CIITA
CXCL9
FASLG
FCRL1
FCRL3
FCRL4
GPR174
GZMK
IFNG
ITGAL
MS4A1
PLA2G2D
PYHIN1
RTP5
SH2D1A
SIRPG
TIFAB
TNFRSF13B
TRAT1
TTC24
UBASH3A
ZNF80
ZNF831
logFC
Up_Regulation
Down_Regulation
Exclusion score
Low
High
Tumor Type
BRCA
COAD
LUAD
LUSC
BLCA
SKCM
KIRC
STAD
ESCA
StromalScore
ImmuneScore
BRCA (n=919)
SKCM (n=412)
LUAD (n=438)
LUSC (n=460)
BLCA (n=372)
COAD (n=422)
KIRC (n=526)
STAD (n=340)
ESCA (n=139)
D
ssGSEA for TCGA pan-cancer
TNBC
COAD
LUAD
KIRC
BLCA
LUSC
SKCM
P value
<0.0001
0.001
0.01
>0.05
Enrichment
NS
Down
Up
B cells memory
B cells naive
Dendritic cells activated
Dendritic cells resting
Eosinophils
Fibroblasts
Macrophage M0
Macrophage M1
Macrophage M2
Mast cells activated
Mast cells resting
Monocytes
Neutrophils
NK cells activated
NK cells resting
Plasma cells
T cells CD4 memory activated
T cells CD4 memory resting
T cells CD4 naive
T cells CD8
T cells follicular helper
T cells gamma delta
Treg

Figure S1

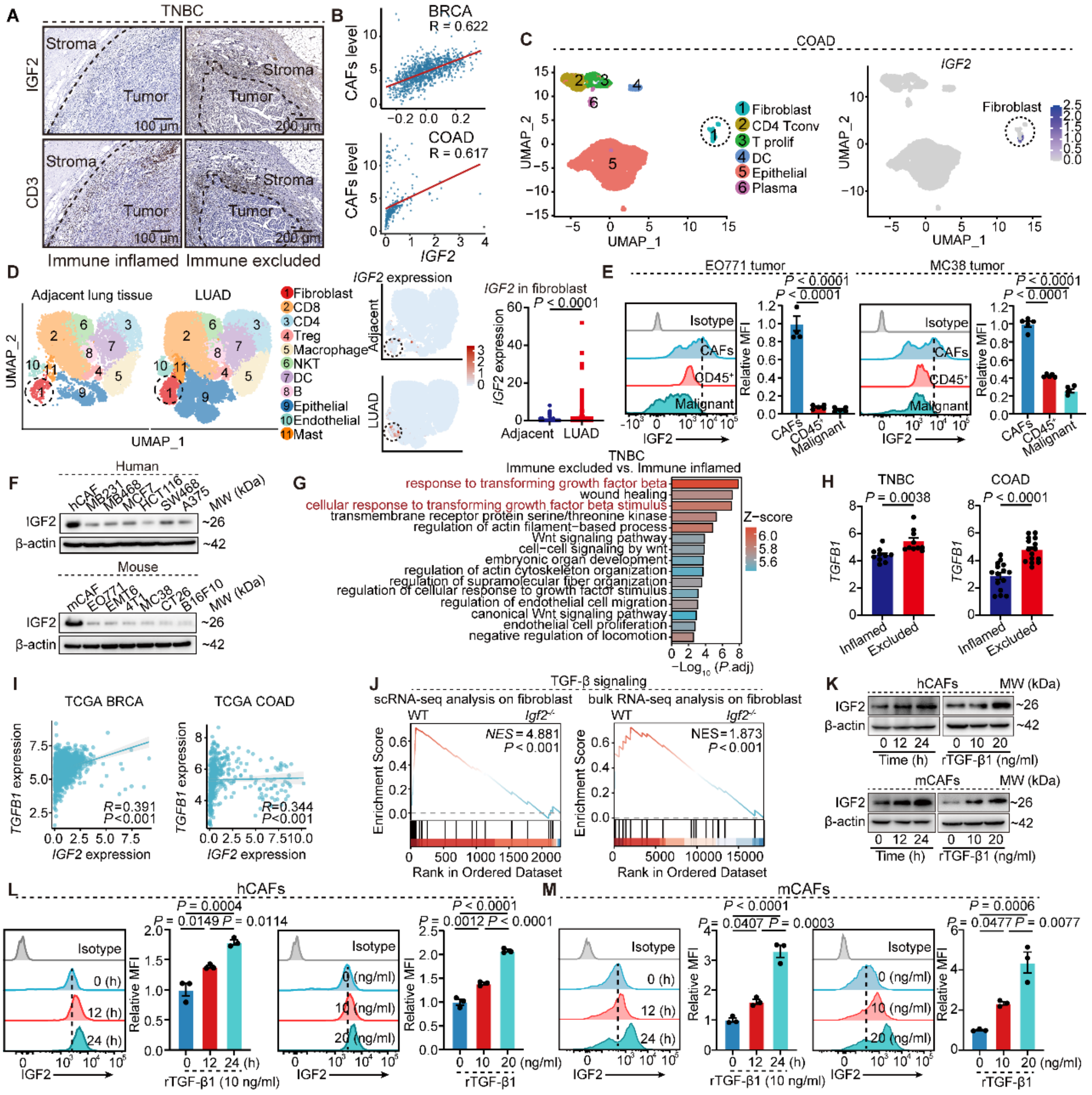

A
TNBC
Stroma
Tumor
IGF2
CD3
100 μm
200 μm
Immune inflamed
Immune excluded
B
BRCA
R = 0.622
COAD
R = 0.617
CAFs level
IGF2
C
COAD
Fibroblast
CD4 Tconv
T prolif
DC
Epithelial
Plasma
UMAP_1
UMAP_2
IGF2
D
Adjacent lung tissue
LUAD
Fibroblast
CD8
CD4
Treg
Macrophage
NKT
DC
B
Epithelial
Endothelial
Mast
IGF2 expression
Adjacent
IGF2 in fibroblast
P < 0.0001
E
EO771 tumor
MC38 tumor
Isotype
CAFs
CD45+
Malignant
Relative MFI
F
Human
hCAF
MB231
MB468
MCF7
HCT116
SW468
A375
MW (kDa)
β-actin
~26
~42
Mouse
mCAF
EO771
EMT6
4T1
MC38
CT26
B16F10
G
TNBC
Immune excluded vs. Immune inflamed
response to transforming growth factor beta
wound healing
cellular response to transforming growth factor beta stimulus
transmembrane receptor protein serine/threonine kinase
regulation of actin filament−based process
Wnt signaling pathway
cell−cell signaling by wnt
embryonic organ development
regulation of actin cytoskeleton organization
regulation of supramolecular fiber organization
regulation of cellular response to growth factor stimulus
regulation of endothelial cell migration
canonical Wnt signaling pathway
endothelial cell proliferation
negative regulation of locomotion
Z-score
−Log10 (P.adj)
H
TNBC
COAD
P = 0.0038
P < 0.0001
TGFB1
Inflamed
Excluded
I
TCGA BRCA
TCGA COAD
TGFB1 expression
IGF2 expression
R=0.391
P<0.001
R=0.344
P<0.001
J
TGF-β signaling
scRNA-seq analysis on fibroblast
bulk RNA-seq analysis on fibroblast
WT
Igf2−/−
NES = 4.881
NES=1.873
Enrichment Score
Rank in Ordered Dataset
K
hCAFs
mCAFs
Time (h)
rTGF-β1 (ng/ml)
L
hCAFs
M
mCAFs
rTGF-β1 (10 ng/ml)
rTGF-β1

Figure S2

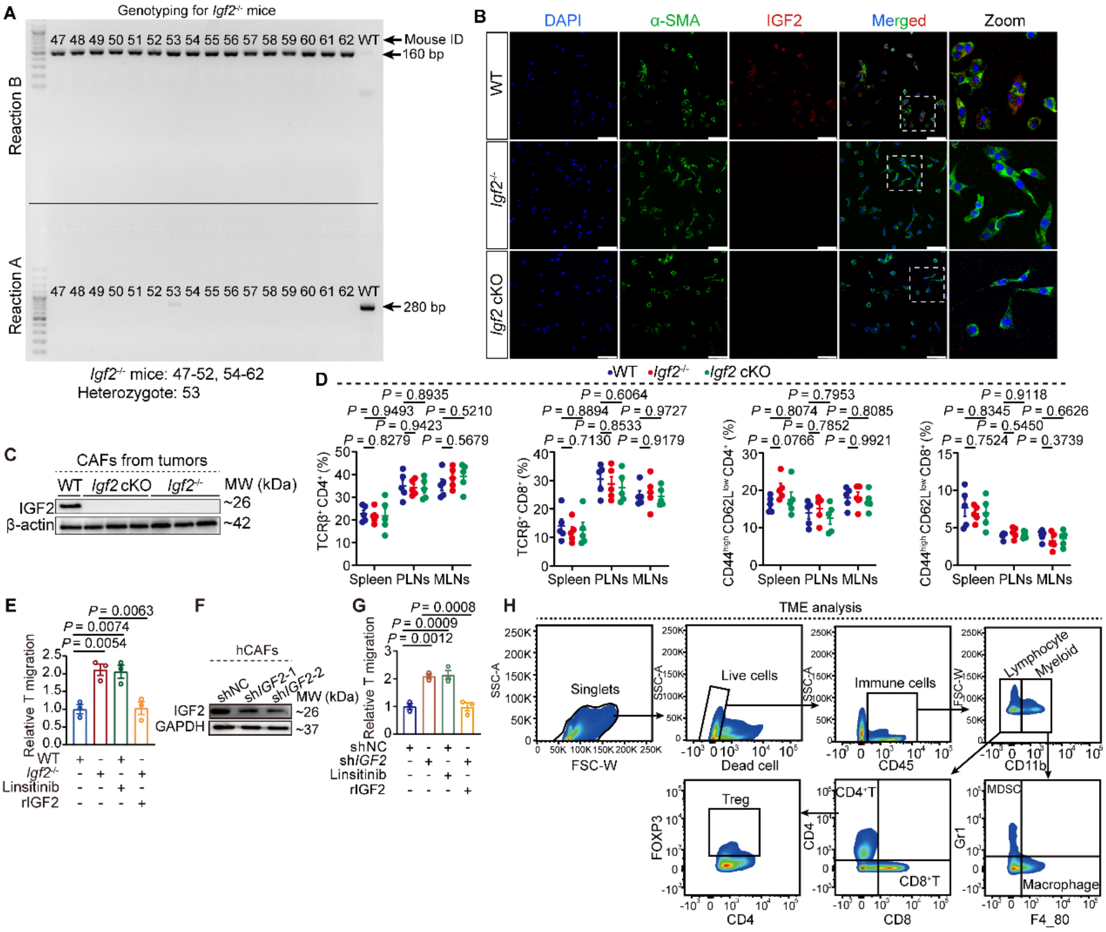
A
Genotyping for Igf2-/- mice
Reaction B
Reaction A
47 48 49 50 51 52 53 54 55 56 57 58 59 60 61 62 WT
Mouse ID
160 bp
280 bp
Igf2-/- mice: 47-52, 54-62
Heterozygote: 53
B
DAPI
α-SMA
IGF2
Merged
Zoom
WT
Igf2-/-
Igf2 cKO
C
CAFs from tumors
WT
Igf2 cKO
Igf2-/-
MW (kDa)
IGF2
β-actin
~26
~42
D
WT
Igf2-/-
Igf2 cKO
Spleen PLNs MLNs
E
Relative T migration
WT
Igf2-/-
Linsitinib
rIGF2
F
hCAFs
shNC
shIGF2-1
shIGF2-2
IGF2
GAPDH
~26
~37
G
shNC
shIGF2
H
TME analysis
Singlets
Live cells
Immune cells
Lymphocyte
Myeloid
Treg
CD4+T
CD8+T
MDSC
Macrophage
FSC-W
Dead cell
CD45
CD11b
CD4
CD8
F4_80
FOXP3
Gr1

Figure S3

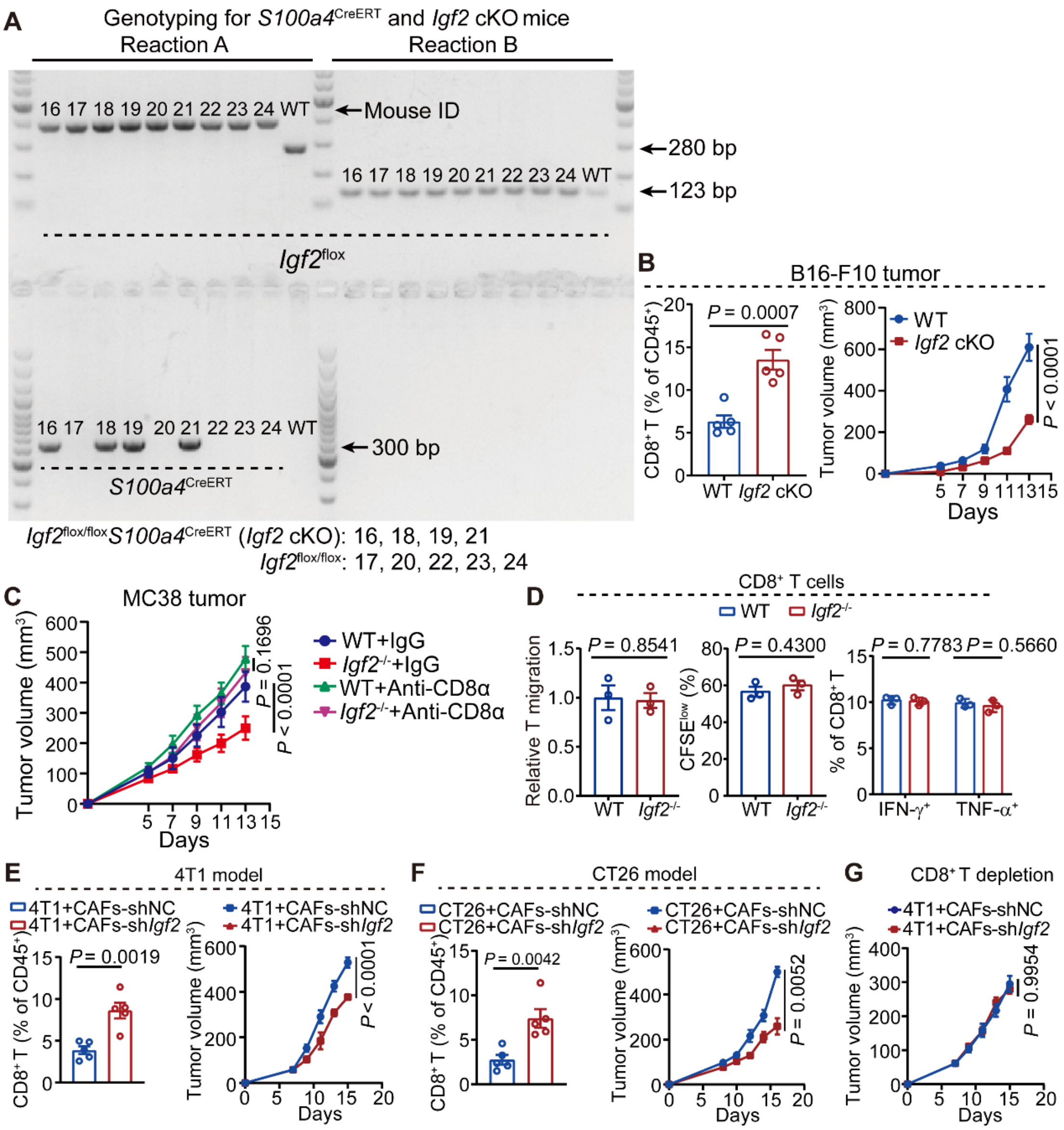
A
Genotyping for S100a4CreERT and Igf2 cKO mice
Reaction A
Reaction B
16 17 18 19 20 21 22 23 24 WT
Mouse ID
280 bp
123 bp
Igf2flox
300 bp
S100a4CreERT
Igf2flox/floxS100a4CreERT (Igf2 cKO): 16, 18, 19, 21
Igf2flox/flox: 17, 20, 22, 23, 24
B
B16-F10 tumor
P = 0.0007
CD8+ T (% of CD45+)
WT Igf2 cKO
Tumor volume (mm3)
WT
Igf2 cKO
P < 0.0001
Days
C
MC38 tumor
Tumor volume (mm3)
P = 0.1696
P < 0.0001
WT+IgG
Igf2-/-+IgG
WT+Anti-CD8α
Igf2-/-+Anti-CD8α
Days
D
CD8+ T cells
WT
Igf2-/-
P = 0.8541
P = 0.4300
P = 0.7783
P = 0.5660
Relative T migration
CFSElow (%)
% of CD8+ T
IFN-γ+
TNF-α+
E
4T1 model
4T1+CAFs-shNC
4T1+CAFs-shIgf2
P = 0.0019
CD8+ T (% of CD45+)
Tumor volume (mm3)
P < 0.0001
Days
F
CT26 model
CT26+CAFs-shNC
CT26+CAFs-shIgf2
P = 0.0042
CD8+ T (% of CD45+)
Tumor volume (mm3)
P = 0.0052
Days
G
CD8+ T depletion
4T1+CAFs-shNC
4T1+CAFs-shIgf2
Tumor volume (mm3)
P = 0.9954
Days

Figure S4

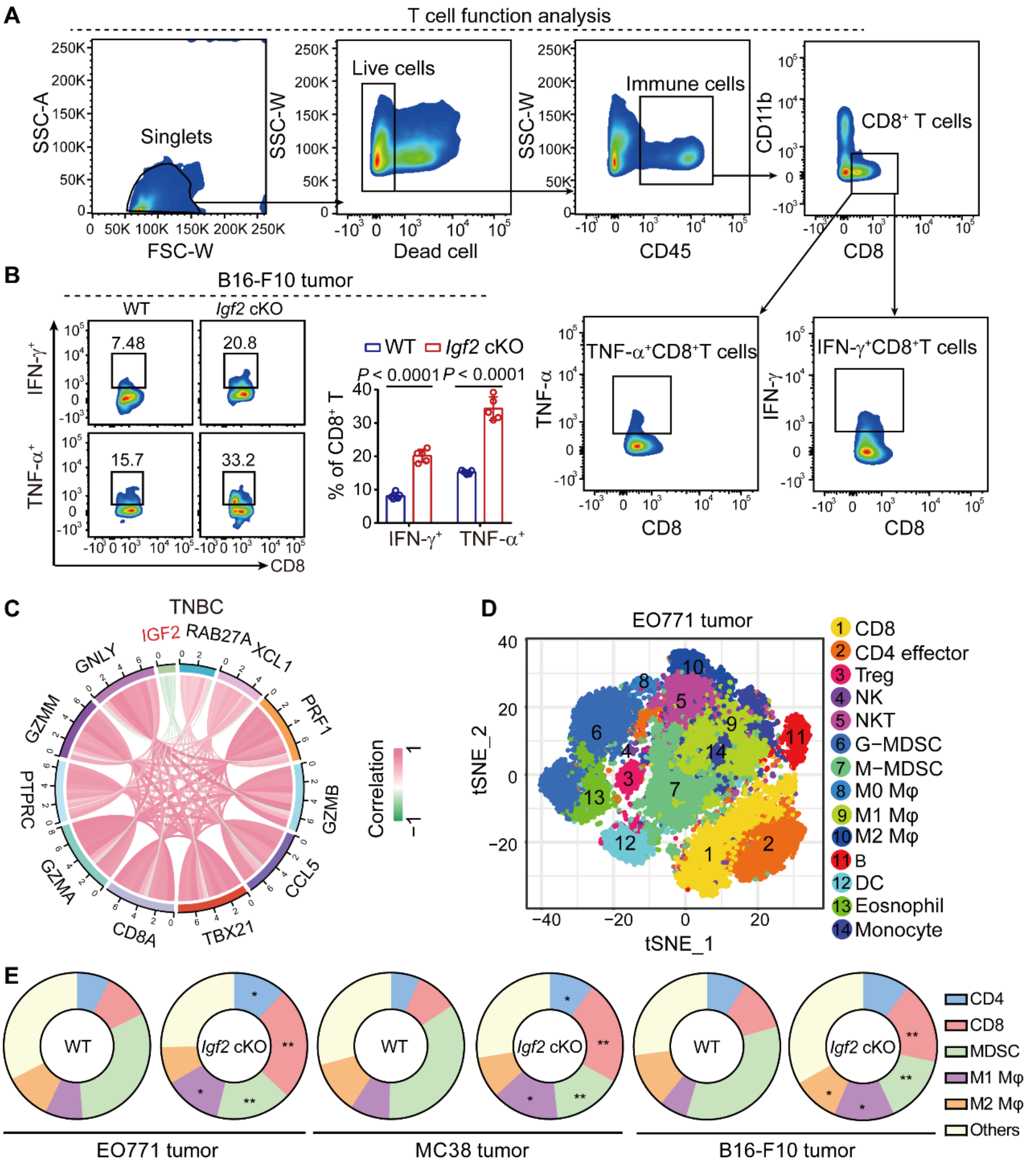
A
T cell function analysis
Singlets
SSC-A
FSC-W
Live cells
SSC-W
Dead cell
Immune cells
CD45
CD8⁺ T cells
CD11b
CD8
TNF-α⁺CD8⁺T cells
TNF-α
IFN-γ⁺CD8⁺T cells
IFN-γ
B
B16-F10 tumor
WT
Igf2 cKO
IFN-γ⁺
TNF-α⁺
7.48
20.8
15.7
33.2
% of CD8⁺ T
P < 0.0001
C
TNBC
IGF2
RAB27A
XCL1
PRF1
GZMB
CCL5
TBX21
CD8A
GZMA
PTPRC
GZMM
GNLY
Correlation
D
EO771 tumor
tSNE_1
tSNE_2
1 CD8
2 CD4 effector
3 Treg
4 NK
5 NKT
6 G−MDSC
7 M−MDSC
8 M0 Mφ
9 M1 Mφ
10 M2 Mφ
11 B
12 DC
13 Eosnophil
14 Monocyte
E
CD4
CD8
MDSC
M1 Mφ
M2 Mφ
Others
EO771 tumor
MC38 tumor
B16-F10 tumor

Figure S5

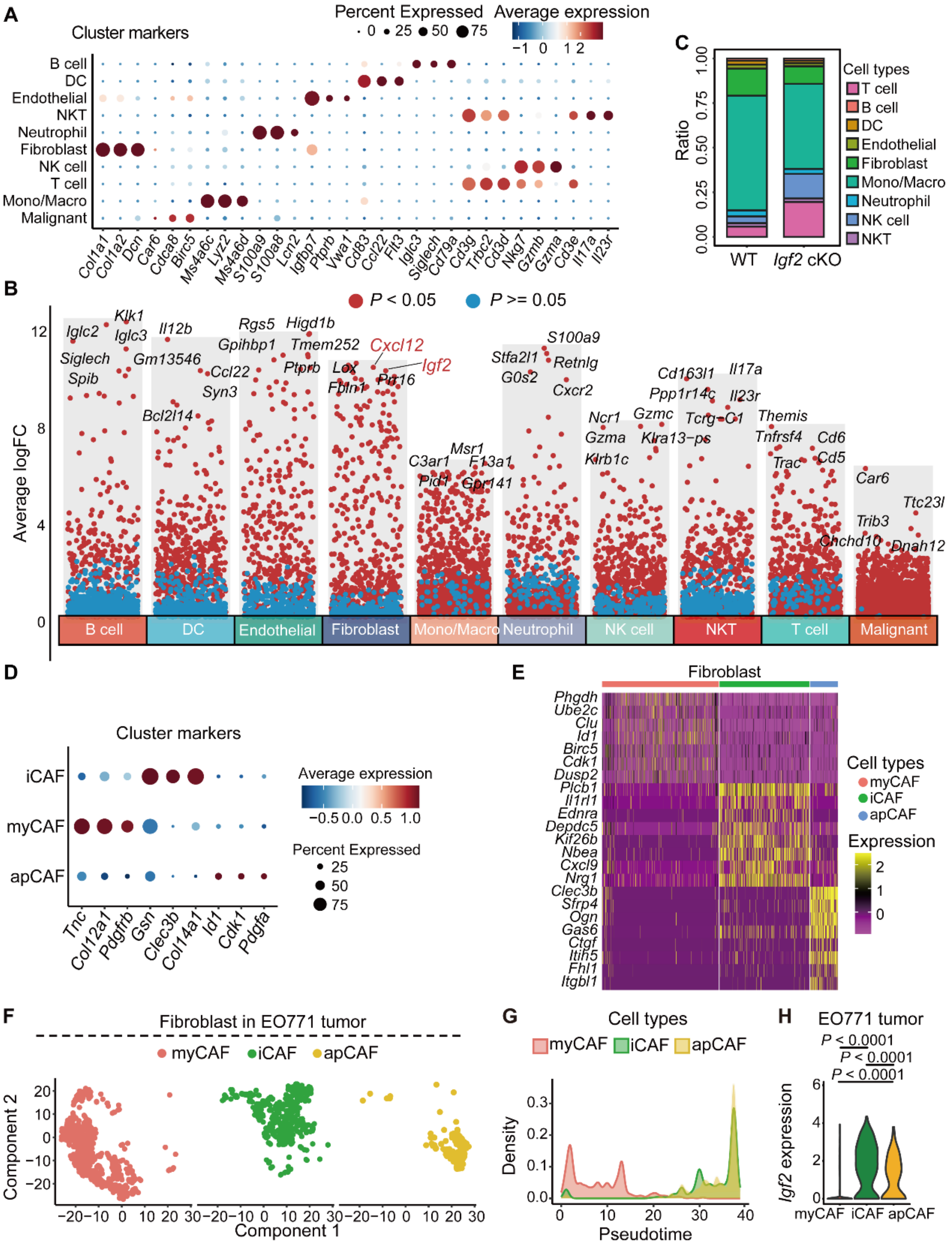

A
Cluster markers
Percent Expressed
Average expression
C
Ratio
WT
Igf2 cKO
Cell types
T cell
B cell
DC
Endothelial
Fibroblast
Mono/Macro
Neutrophil
NK cell
NKT
B
P < 0.05
P >= 0.05
Average logFC
Cxcl12
Igf2
B cell
DC
Endothelial
Fibroblast
Mono/Macro
Neutrophil
NK cell
NKT
T cell
Malignant
D
Cluster markers
iCAF
myCAF
apCAF
Average expression
Percent Expressed
E
Fibroblast
Cell types
myCAF
iCAF
apCAF
Expression
F
Fibroblast in EO771 tumor
Component 2
Component 1
G
Cell types
Density
Pseudotime
H
EO771 tumor
P < 0.0001
Igf2 expression
myCAF
iCAF
apCAF

Figure S6

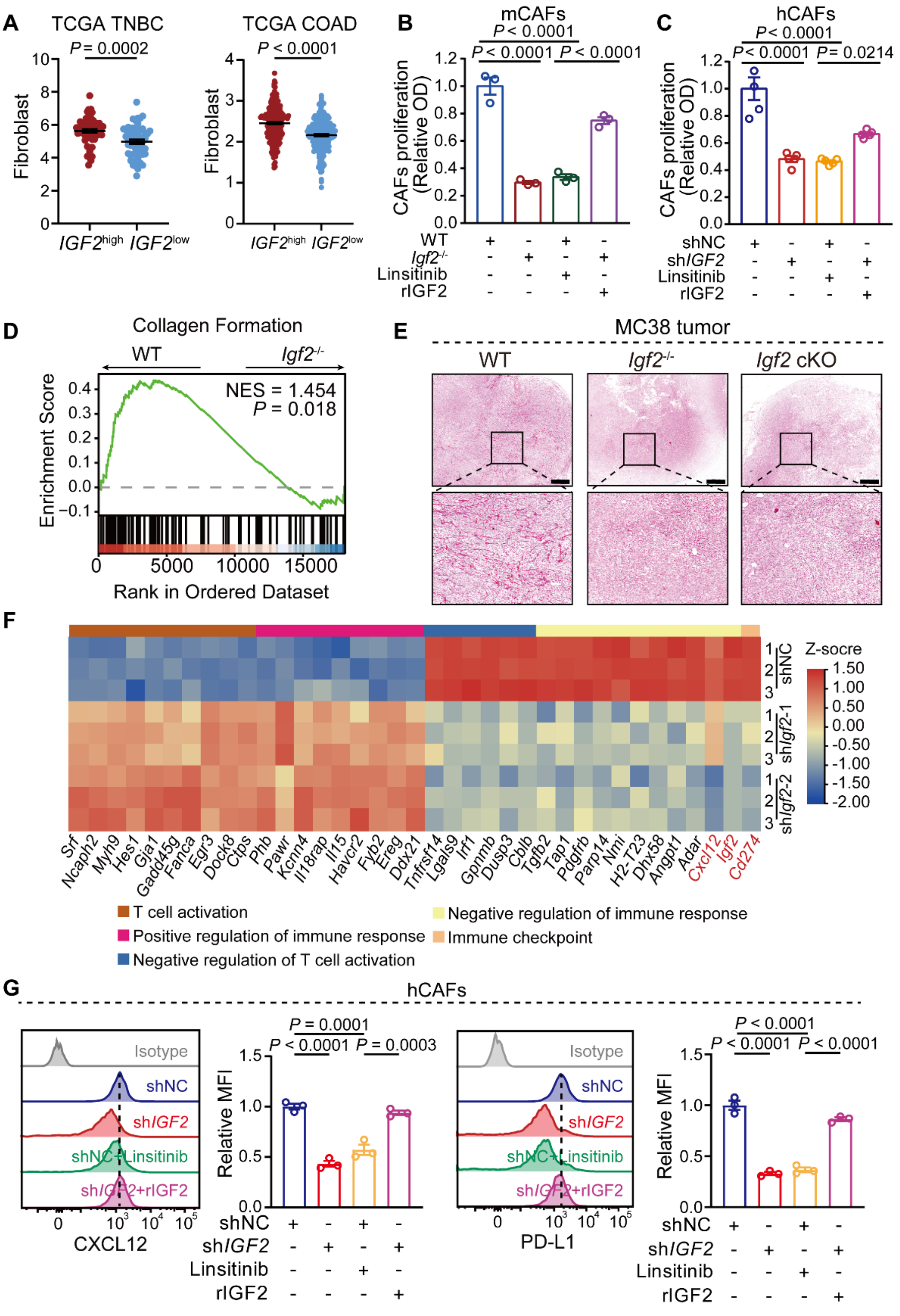
A
TCGA TNBC
P = 0.0002
Fibroblast
IGF2high IGF2low
TCGA COAD
P < 0.0001
B
mCAFs
P < 0.0001
P < 0.0001 P < 0.0001
CAFs proliferation (Relative OD)
WT
Igf2-/-
Linsitinib
rIGF2
C
hCAFs
P < 0.0001
P < 0.0001 P = 0.0214
shNC
shIGF2
D
Collagen Formation
WT
Igf2-/-
NES = 1.454
P = 0.018
Enrichment Score
Rank in Ordered Dataset
E
MC38 tumor
Igf2 cKO
F
Z-socre
T cell activation
Positive regulation of immune response
Negative regulation of T cell activation
Negative regulation of immune response
Immune checkpoint
shIgf2-1
shIgf2-2
G
hCAFs
Isotype
shNC+Linsitinib
shIGF2+rIGF2
CXCL12
PD-L1
Relative MFI
P = 0.0001
P < 0.0001 P = 0.0003
P < 0.0001
P < 0.0001 P < 0.0001

Figure S7

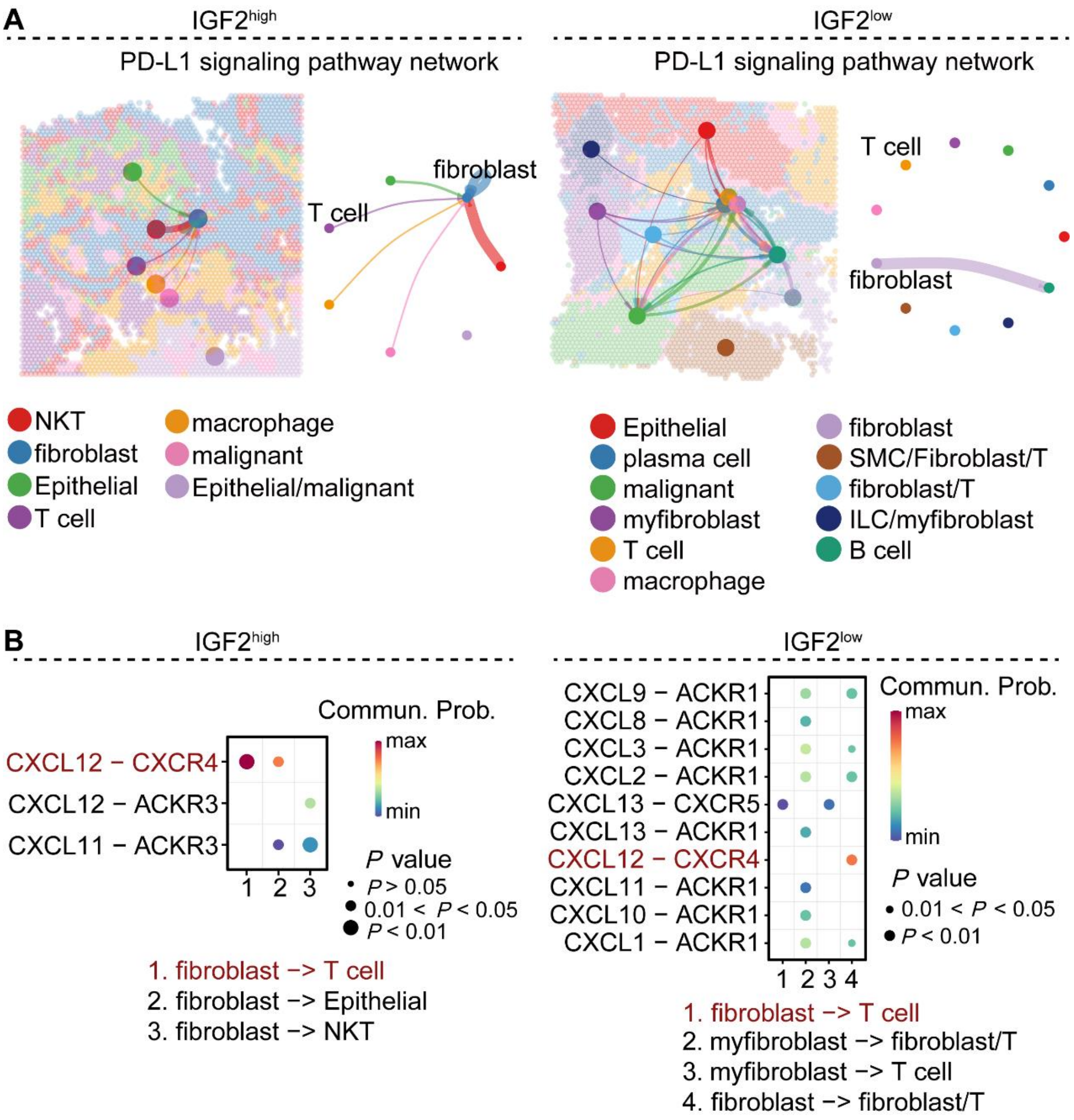
A
IGF2^high
PD-L1 signaling pathway network
fibroblast
T cell
NKT
fibroblast
Epithelial
T cell
macrophage
malignant
Epithelial/malignant
IGF2^low
PD-L1 signaling pathway network
T cell
fibroblast
Epithelial
plasma cell
malignant
myfibroblast
T cell
macrophage
fibroblast
SMC/Fibroblast/T
fibroblast/T
ILC/myfibroblast
B cell
B
IGF2^high
Commun. Prob.
max
min
CXCL12 − CXCR4
CXCL12 − ACKR3
CXCL11 − ACKR3
1 2 3
P value
P > 0.05
0.01 < P < 0.05
P < 0.01
1. fibroblast −> T cell
2. fibroblast −> Epithelial
3. fibroblast −> NKT
IGF2^low
CXCL9 − ACKR1
CXCL8 − ACKR1
CXCL3 − ACKR1
CXCL2 − ACKR1
CXCL13 − CXCR5
CXCL13 − ACKR1
CXCL12 − CXCR4
CXCL11 − ACKR1
CXCL10 − ACKR1
CXCL1 − ACKR1
1 2 3 4
Commun. Prob.
max
min
P value
0.01 < P < 0.05
P < 0.01
1. fibroblast −> T cell
2. myfibroblast −> fibroblast/T
3. myfibroblast −> T cell
4. fibroblast −> fibroblast/T

Figure S8

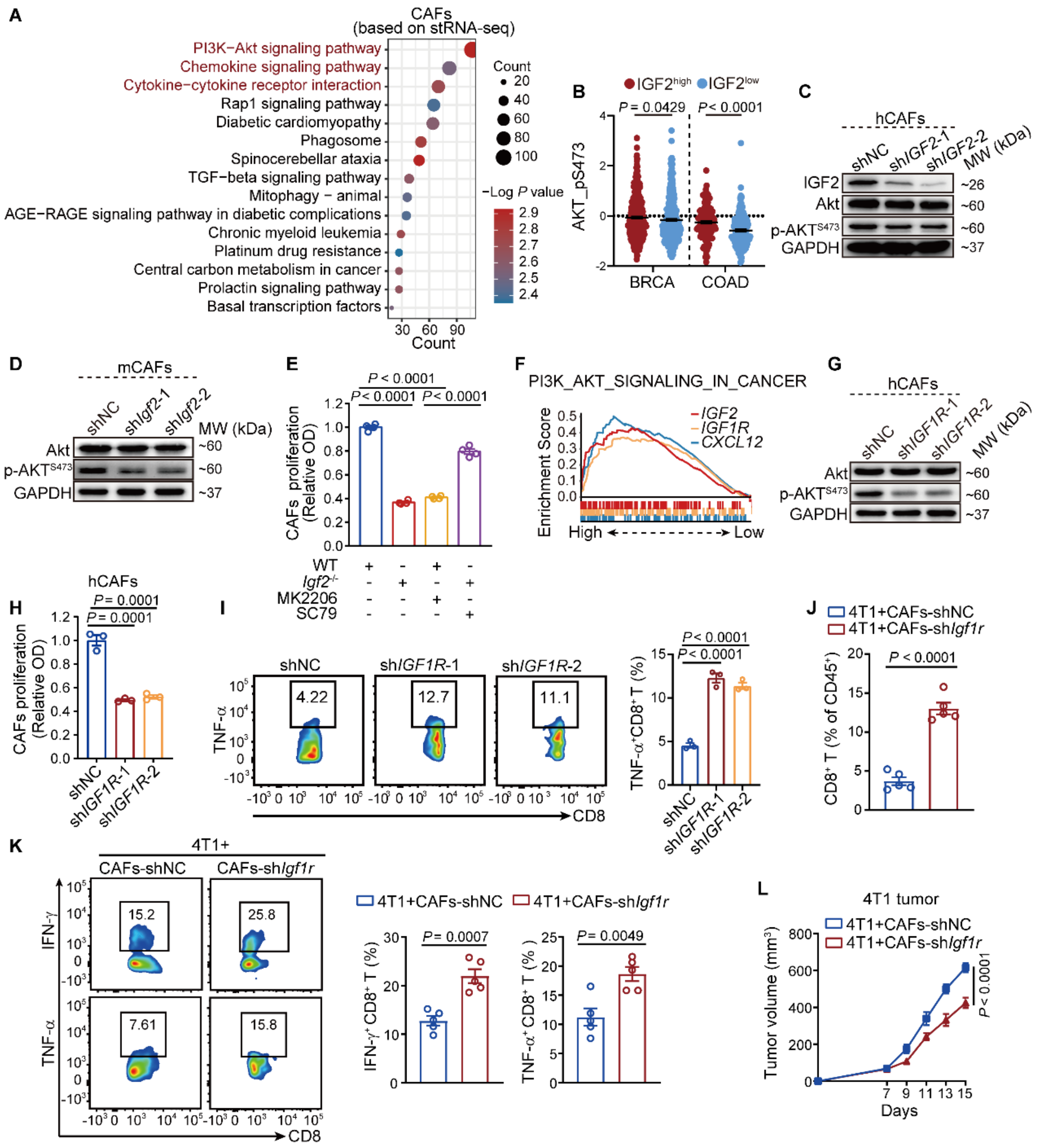
A
CAFs (based on stRNA-seq)
PI3K−Akt signaling pathway
Chemokine signaling pathway
Cytokine−cytokine receptor interaction
Rap1 signaling pathway
Diabetic cardiomyopathy
Phagosome
Spinocerebellar ataxia
TGF−beta signaling pathway
Mitophagy − animal
AGE−RAGE signaling pathway in diabetic complications
Chronic myeloid leukemia
Platinum drug resistance
Central carbon metabolism in cancer
Prolactin signaling pathway
Basal transcription factors
Count
−Log P value
B
IGF2high IGF2low
P = 0.0429
P < 0.0001
AKT_pS473
BRCA
COAD
C
hCAFs
shNC
shIGF2-1
shIGF2-2
MW (kDa)
IGF2
Akt
p-AKTS473
GAPDH
D
mCAFs
shNC
shIgf2-1
shIgf2-2
E
CAFs proliferation (Relative OD)
P < 0.0001
WT
Igf2-/-
MK2206
SC79
F
PI3K_AKT_SIGNALING_IN_CANCER
Enrichment Score
IGF2
IGF1R
CXCL12
High
Low
G
hCAFs
shIGF1R-1
shIGF1R-2
H
P = 0.0001
I
TNF-α
CD8
4.22
12.7
11.1
TNF-α+CD8+ T (%)
J
4T1+CAFs-shNC
4T1+CAFs-shIgf1r
CD8+ T (% of CD45+)
K
4T1+
CAFs-shNC
CAFs-shIgf1r
IFN-γ
15.2
25.8
7.61
15.8
IFN-γ+ CD8+ T (%)
P = 0.0007
P = 0.0049
L
4T1 tumor
Tumor volume (mm3)
Days

Figure S9

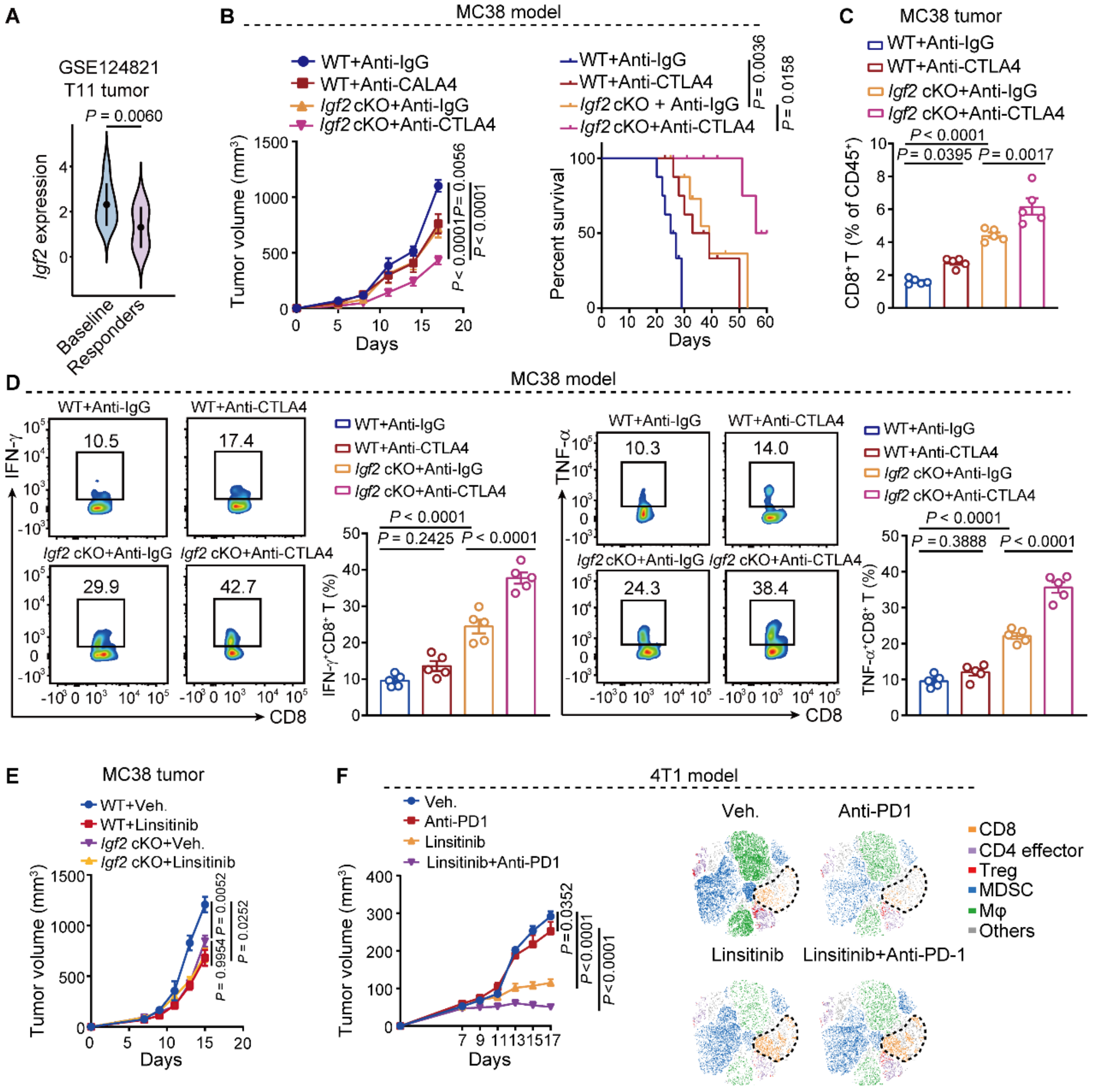
A
GSE124821
T11 tumor
P = 0.0060
Igf2 expression
Baseline
Responders
B
MC38 model
WT+Anti-IgG
WT+Anti-CALA4
Igf2 cKO+Anti-IgG
Igf2 cKO+Anti-CTLA4
Tumor volume (mm³)
Days
P = 0.0056
P < 0.0001
P < 0.0001
WT+Anti-IgG
WT+Anti-CTLA4
Igf2 cKO + Anti-IgG
Igf2 cKO+Anti-CTLA4
P = 0.0036
P = 0.0158
Percent survival
Days
C
MC38 tumor
WT+Anti-IgG
WT+Anti-CTLA4
Igf2 cKO+Anti-IgG
Igf2 cKO+Anti-CTLA4
P < 0.0001
P = 0.0395
P = 0.0017
CD8⁺ T (% of CD45⁺)
D
MC38 model
WT+Anti-IgG
WT+Anti-CTLA4
Igf2 cKO+Anti-IgG
Igf2 cKO+Anti-CTLA4
IFN-γ
CD8
10.5
17.4
29.9
42.7
P < 0.0001
P = 0.2425
P < 0.0001
IFN-γ⁺CD8⁺ T (%)
TNF-α
10.3
14.0
24.3
38.4
P < 0.0001
P = 0.3888
P < 0.0001
TNF-α⁺CD8⁺ T (%)
E
MC38 tumor
WT+Veh.
WT+Linsitinib
Igf2 cKO+Veh.
Igf2 cKO+Linsitinib
Tumor volume (mm³)
Days
P = 0.0052
P = 0.9954
P = 0.0252
F
4T1 model
Veh.
Anti-PD1
Linsitinib
Linsitinib+Anti-PD1
Tumor volume (mm³)
Days
P = 0.0352
P < 0.0001
P < 0.0001
Veh.
Anti-PD1
Linsitinib
Linsitinib+Anti-PD-1
CD8
CD4 effector
Treg
MDSC
Mφ
Others

Figure S10

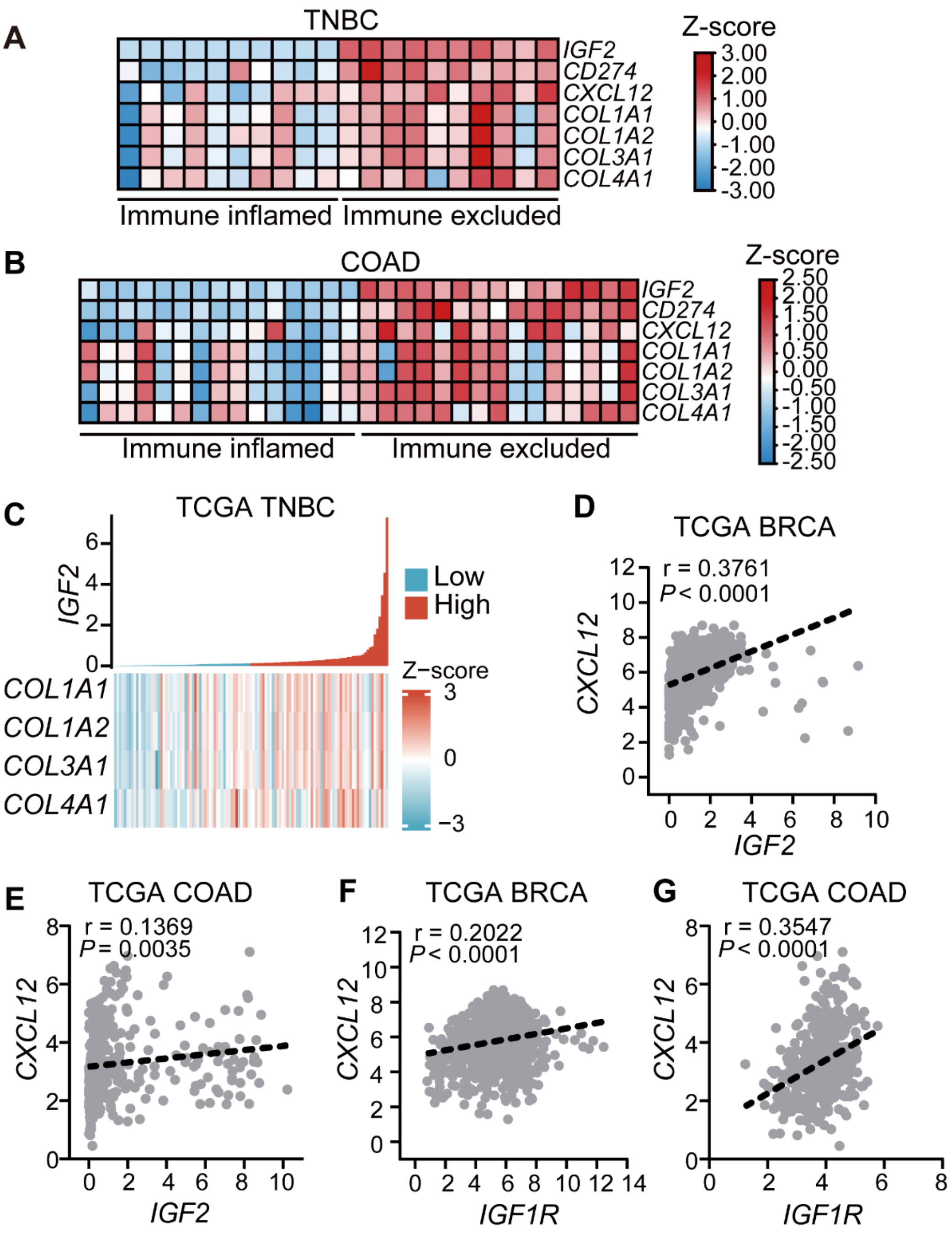
A
TNBC
IGF2
CD274
CXCL12
COL1A1
COL1A2
COL3A1
COL4A1
Z-score
3.00
2.00
1.00
0.00
-1.00
-2.00
-3.00
Immune inflamed
Immune excluded
B
COAD
IGF2
CD274
CXCL12
COL1A1
COL1A2
COL3A1
COL4A1
Z-score
2.50
2.00
1.50
1.00
0.50
0.00
-0.50
-1.00
-1.50
-2.00
-2.50
Immune inflamed
Immune excluded
C
TCGA TNBC
IGF2
Low
High
COL1A1
COL1A2
COL3A1
COL4A1
Z-score
3
0
−3
D
TCGA BRCA
r = 0.3761
P < 0.0001
CXCL12
IGF2
E
TCGA COAD
r = 0.1369
P = 0.0035
CXCL12
IGF2
F
TCGA BRCA
r = 0.2022
P < 0.0001
CXCL12
IGF1R
G
TCGA COAD
r = 0.3547
P < 0.0001
CXCL12
IGF1R

Figure S11

Table 1. Chemicals and Antibodies used in this study

**Chemical**	**Vendors**	**Catalog no.**
Tamoxifen	Sigma	T5648
Linsitinib	Selleck	S1091
Diphtheria toxin	Sigma	D0564
Viability Dye eFluor 450	Invitrogen	65-0863-14
Foxp3/Transcription Factor Staining Buffer Set	eBioscience	2507021
Cell Stimulation Cocktail	Invitrogen	00-4975-93
cisplatin	MedChemExpress	HY-17394
Fixation/Permeabilization Buffer	eBioscience	88-8824-00
MK2206	MedChemExpress	HY-10358
SC79	MedChemExpress	HY-18749
Transfection Reagent	SignaGen	SL100688
Human IGF2 ELISA kit	Sabbiotech	EK1133-2
Mouse CXCL12 ELISA kit	Boster	EK0500
**Antibody**	**Vendors**	**Catalog no.**
anti-PD-1 antibody	Bioxcell	RMP1-14
anti-CTLA-4 antibody	Bioxcell	9D9
anti-CD8 antibody	Bioxcell	YTS 169.4
anti-CD16/32 antibody	BioLegend	101302
anti-CD45	BioLegend	30F11
anti-CD4	BioLegend	GK1.5
anti-CD8a	BioLegend	53-6.7
anti-CD11b	BioLegend	M1/70
anti-Gr-1	BioLegend	RB6-8C5
anti-F4/80	BioLegend	BM8
anti-CD206	BioLegend	C06.8C2
anti-MHCII	BioLegend	M5/114.15.2
anti-CD140a	BioLegend	APA5
anti-PD-L1	BioLegend	10F.9G2
anti-FOXP3	BioLegend	MF-14
anti-IFN-γ	BioLegend	XMG1.2
anti-CD140a	BioLegend	16A1
anti-CD45	BioLegend	2D1
anti-CD326	BioLegend	9C4
anti-TNF-α	BioLegend	MP6-XT22
anti-CD44	BioLegend	IM7
anti-CD62L	BioLegend	MEL-14
anti-TCRβ	BioLegend	H57-597

anti-CD45	Fluidigm	89Y
anti-CD4	Fluidigm	175Lu
anti-PD-1	Fluidigm	173Yb
anti-CD11b	Fluidigm	143Nd
anti-Siglec F	Fluidigm	144Nd
anti-CD69	Fluidigm	153Eu
anti-CD68	Fluidigm	164Dy
anti-CD206	Fluidigm	169Tm
anti-Tbet	Fluidigm	148Sm
anti-CD103	Fluidigm	151Eu
anti-CD3	Fluidigm	174Yb
anti-CD14	Fluidigm	156Gd
anti-CD40	Fluidigm	160Dy
anti-TIGIT	Fluidigm	155Gd
anti-CD127	Fluidigm	141Pr
anti-B220	Fluidigm	176Lu
anti-TIM3	Fluidigm	162Dy
anti-CD223	Fluidigm	158Gd
anti-CD25	Fluidigm	147Sm
anti-F4/80	Fluidigm	159Tb
anti-Ki67	Fluidigm	161Dy
anti-Foxp3	Fluidigm	165Ho
anti-CD19	Fluidigm	149Sm
anti-GATA3	Fluidigm	145Nd
anti-NK1.1	Fluidigm	142Ce
anti-CD8	Fluidigm	168Er
anti-CD86	Fluidigm	172Yb
anti-ly-6G	Fluidigm	152Gd
anti-ly-6C	Fluidigm	163Dy
anti-MHCII	Fluidigm	209Bi
anti-CD80	Fluidigm	171Yb
anti-CTLA-4	Fluidigm	154Gg
anti-CD11c	Fluidigm	150Sm
anti-CD3	Abcam	ab16669
anti-IGF2	Thermo Fisher	MA5-17096
anti-SDF1	Huabio	ER1902-35
anti-α-SMA	Abcam	ab7817
recombinant mouse IGF2 protein	Abcam	ab233634
human IGF2 protein	Abcam	ab155617
human TGFB1 Protein	MedChemExpress	HY-P7118
mouse TGFB1 Protein	MedChemExpress	HY-P70648

anti-IGF1R	Cell Signaling Technology	17174
anti-AKT	Cell Signaling Technology	4691
anti-p-AKT	Cell Signaling Technology	4060
anti-GAPDH	Proteintech	60004-1-Ig
anti-β-Actin	Proteintech	66009-1-Ig

References

1. Kim HJ, et al. Reprogramming of cancer-associated fibroblasts by apoptotic cancer cells inhibits lung metastasis via Notch1-WISP-1 signaling. *Cell Mol Immunol*. 2022;19(12):1373-1391.
2. Jiang P, et al. Signatures of T cell dysfunction and exclusion predict cancer immunotherapy response. *Nat Med*. 2018;24(10):1550-1558.
3. Li H, et al. The allergy mediator histamine confers resistance to immunotherapy in cancer patients via activation of the macrophage histamine receptor H1. *Cancer Cell*. 2022;40(1):36-52.e39.
4. Cerezo-Wallis D, et al. Midkine rewires the melanoma microenvironment toward a tolerogenic and immune-resistant state. *Nat Med*. 2020;26(12):1865-1877.
5. Chapoval AI, et al. B7-H3: a costimulatory molecule for T cell activation and IFN-gamma production. *Nat Immunol*. 2001;2(3):269-274.
6. Sica GL, et al. B7-H4, a molecule of the B7 family, negatively regulates T cell immunity. *Immunity*. 2003;18(6):849-861.
7. Wu Y, et al. FGFR blockade boosts T cell infiltration into triple-negative breast cancer by regulating cancer-associated fibroblasts. *Theranostics*. 2022;12(10):4564-4580.
