# Supplementary material for: Insulin-like growth factor 2 drives fibroblast-mediated tumor immunoevasion and confers resistance to immunotherapy
Source: J Clin Invest. 2024 Nov 15;134(22):e183366. doi: 10.1172/JCI183366 (PMC11563680; doi:10.1172/JCI183366)
Supplement: Unedited blot and gel images [file jci-134-183366-s271.pdf]

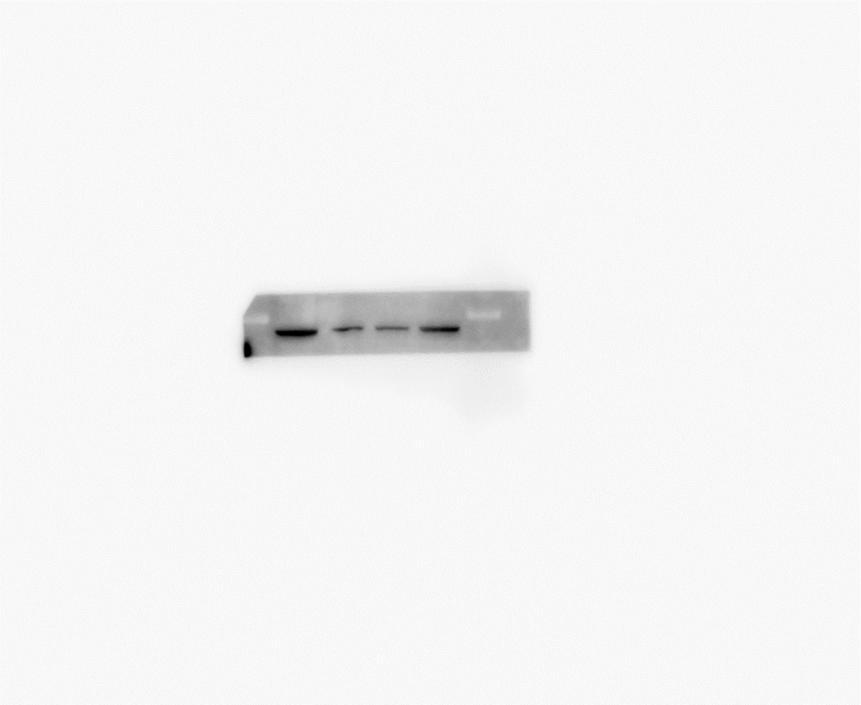

Full unedited blot for Figure 5C p-AKT

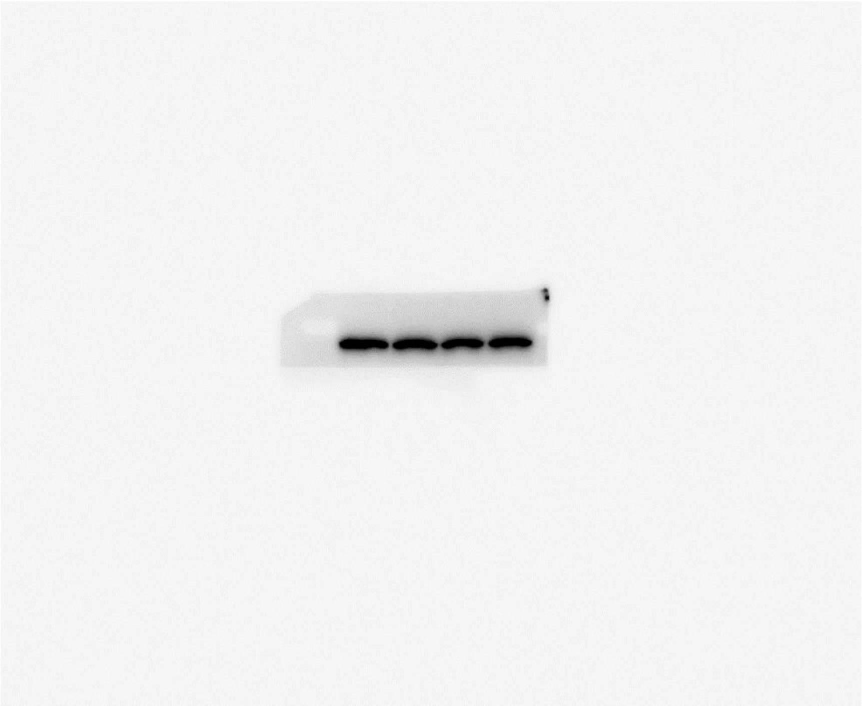

Full unedited blot for Figure 5C AKT

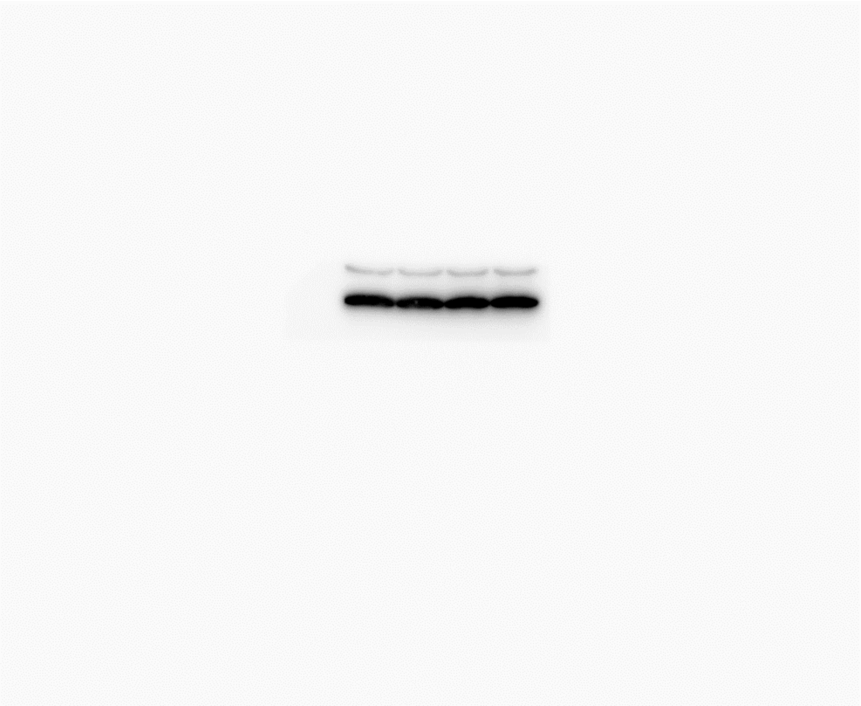

Full unedited blot for Figure 5C GAPDH

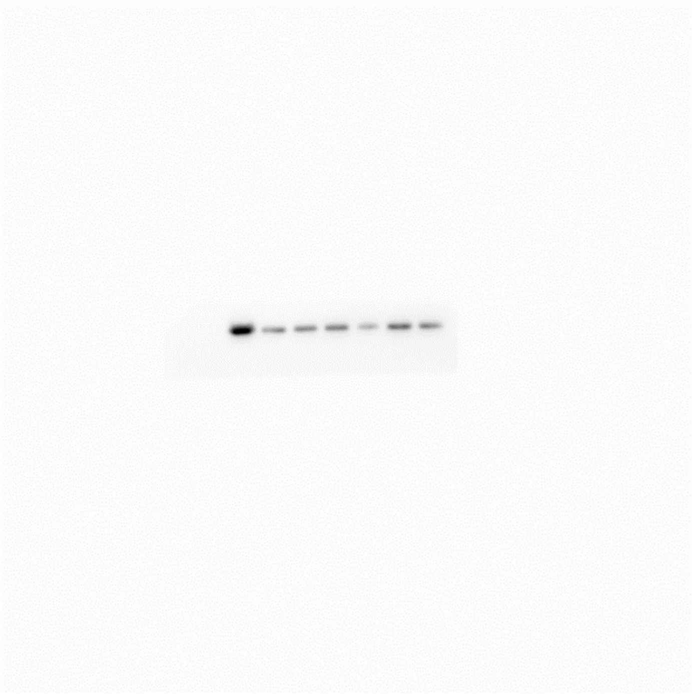

Full unedited blot for Supplemental Figure 2F human IGF2

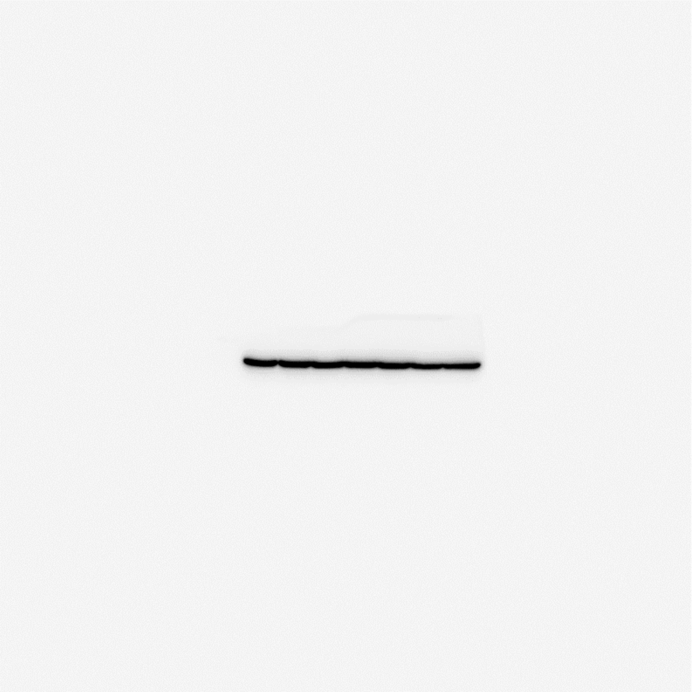

Full unedited blot for Supplemental Figure 2F human β-actin

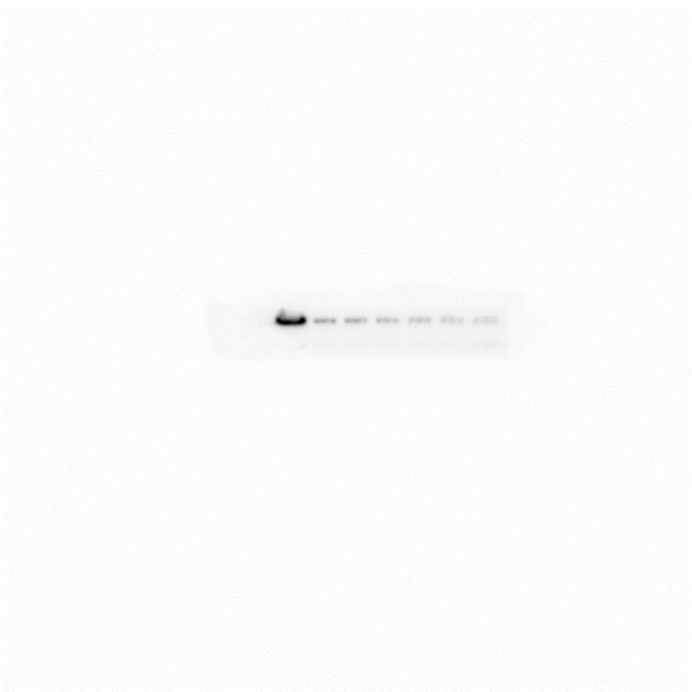

Full unedited blot for Supplemental Figure 2F mouse IGF2

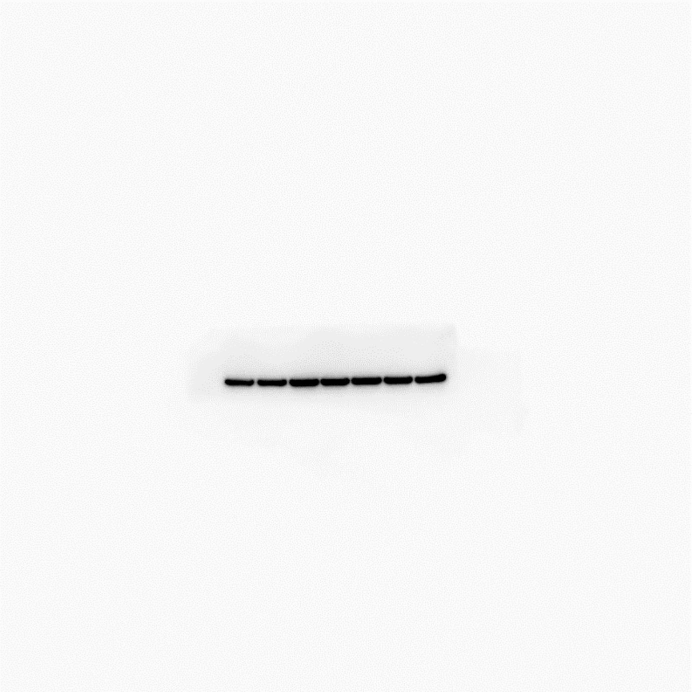

Full unedited blot for Supplemental Figure 2F mouse β-actin

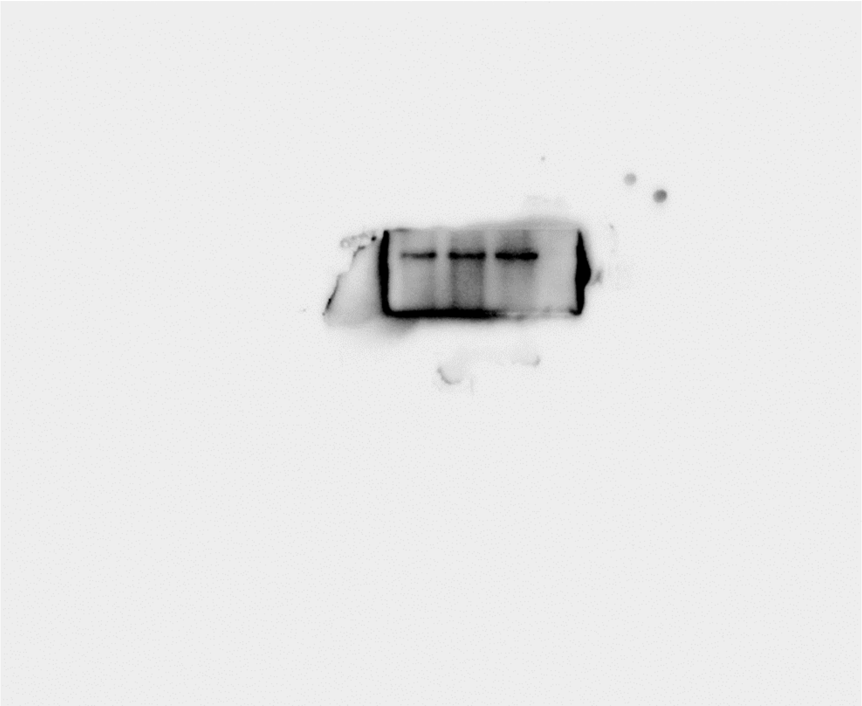

Full unedited blot for Supplemental Figure 2K IGF2 hCAF, distinct time of rTGFβ1

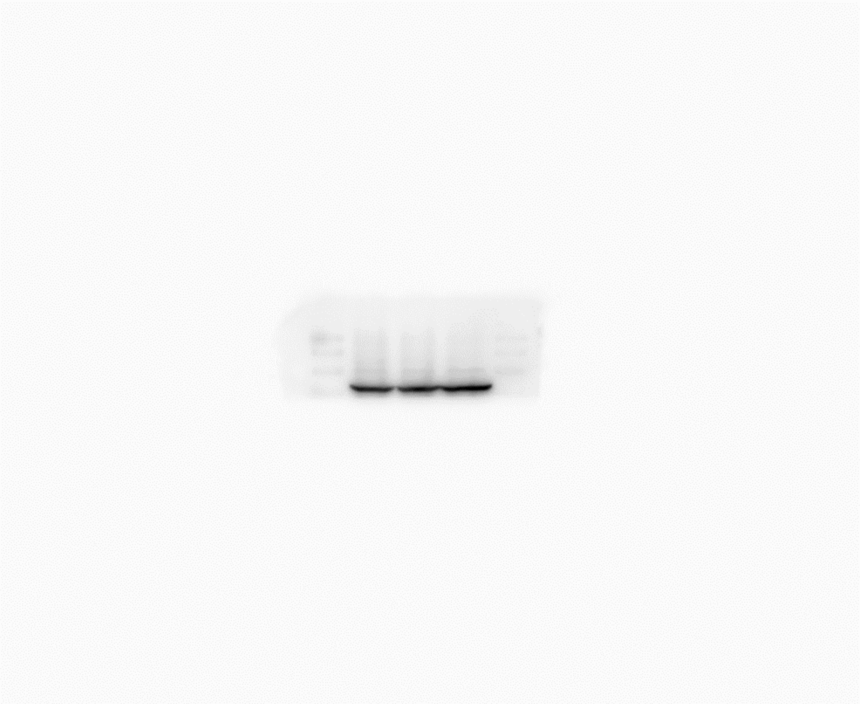

Full unedited blot for Supplemental Figure 2K β-actin hCAF, distinct time of rTGFβ1

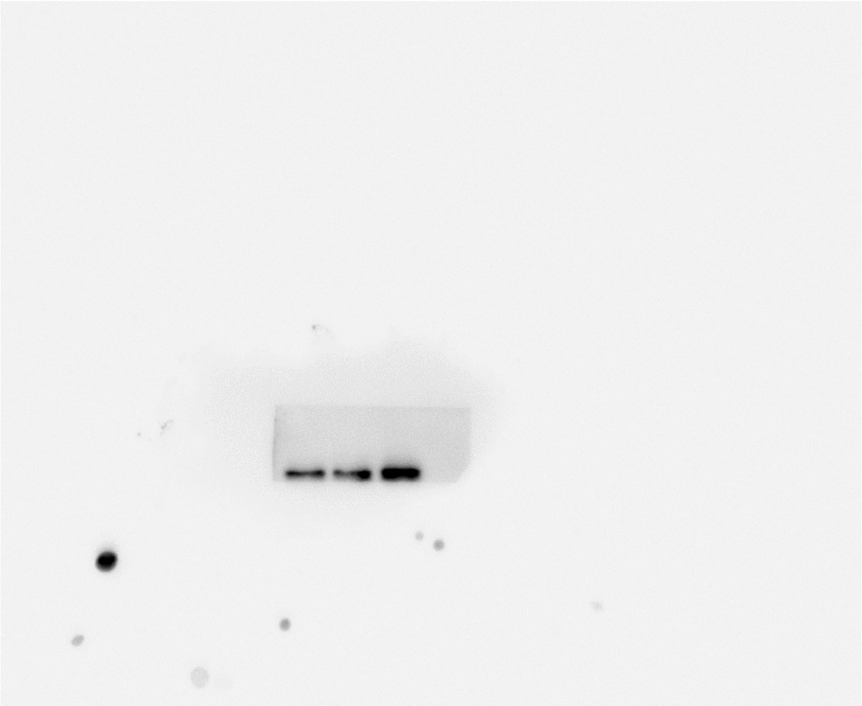

Full unedited blot for Supplemental Figure 2K IGF2 hCAFs, distinct concentration of rTGFβ1

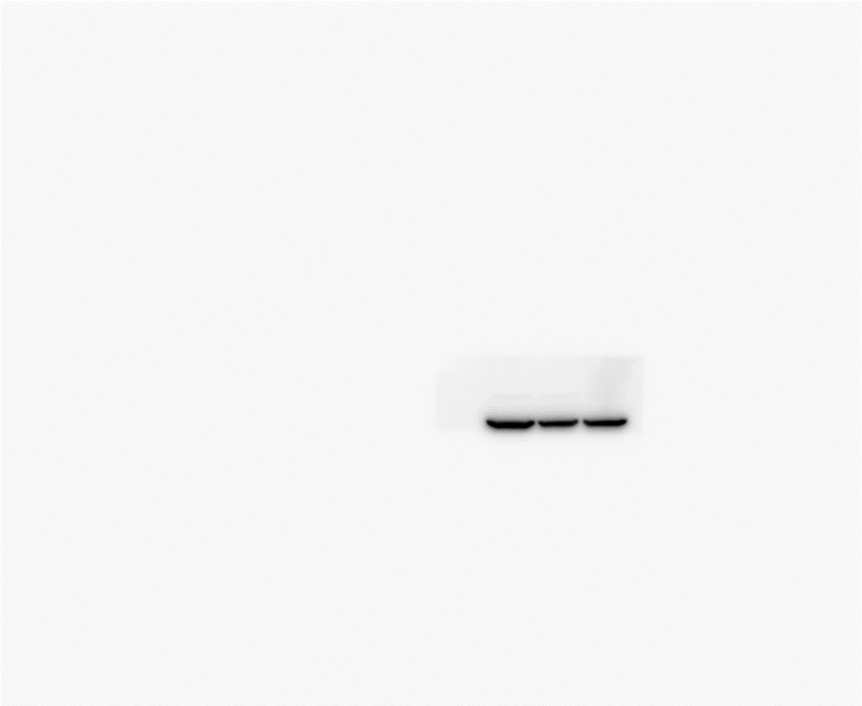

Full unedited blot for Supplemental Figure 2K β-actin hCAF, distinct concentration of rTGFβ1

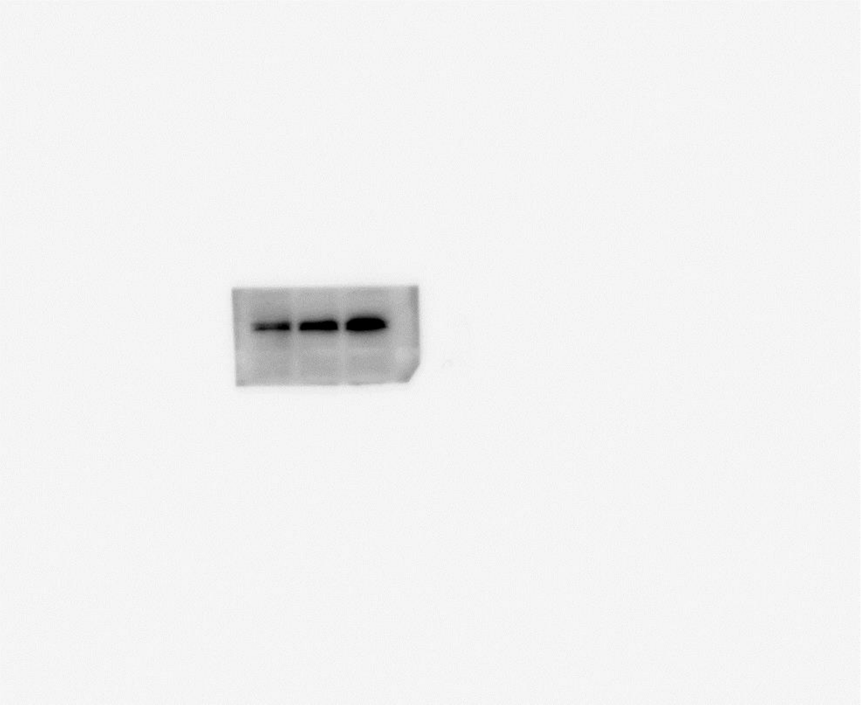

Full unedited blot for Supplemental Figure 2K IGF2 mCAF, distinct time of rTGFβ1

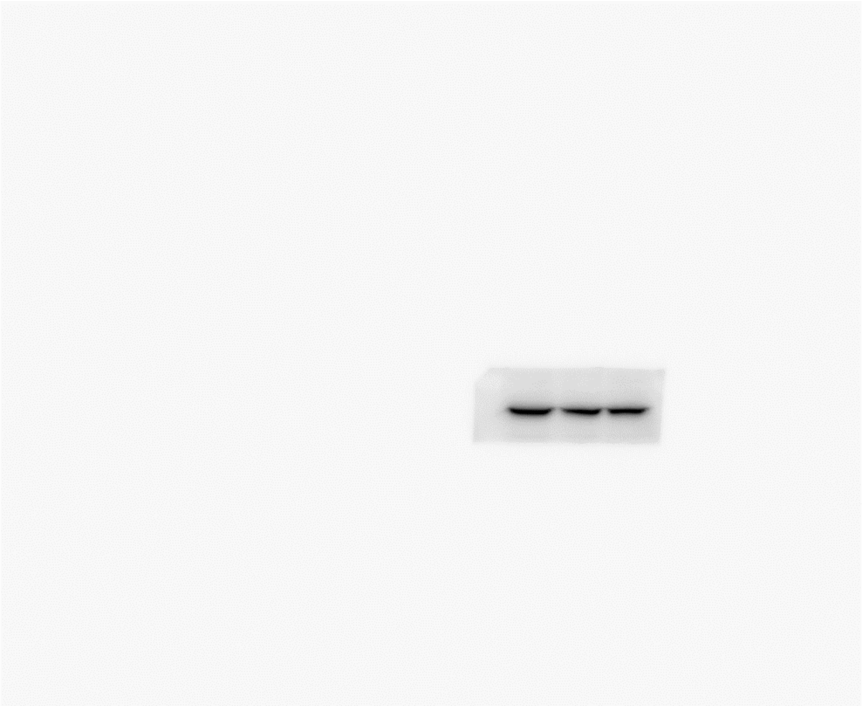

Full unedited blot for Supplemental Figure 2K β-actin mCAF, distinct time of rTGFβ1

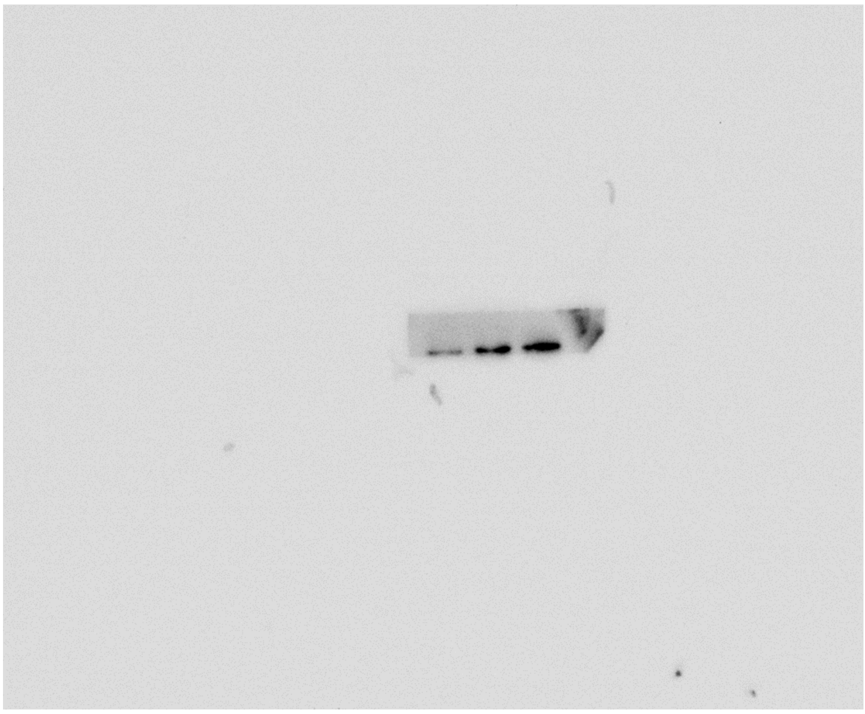

Full unedited blot for Supplemental Figure 2K IGF2 mCAF, distinct concentration of rTGFβ1

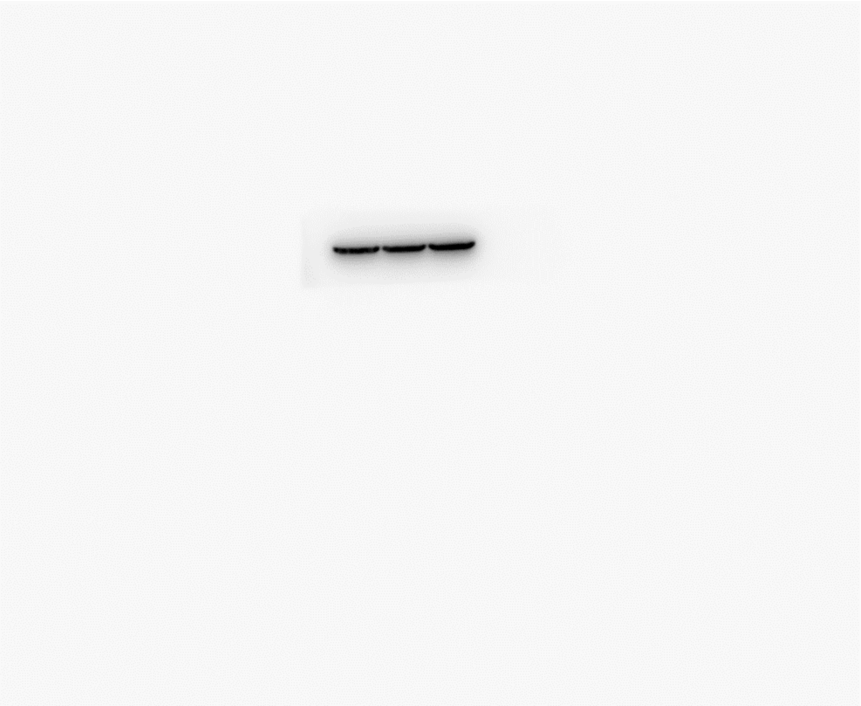

Full unedited blot for Supplemental Figure 2K β-actin mCAF, distinct concentration of rTGFβ1

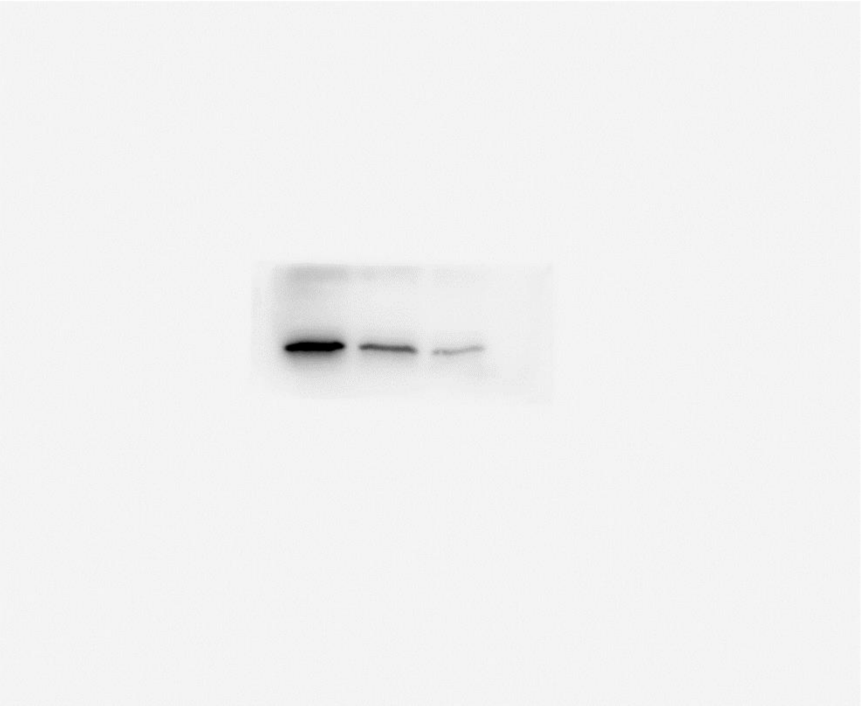

Full unedited blot for Supplemental Figure 9C IGF2 hCAF

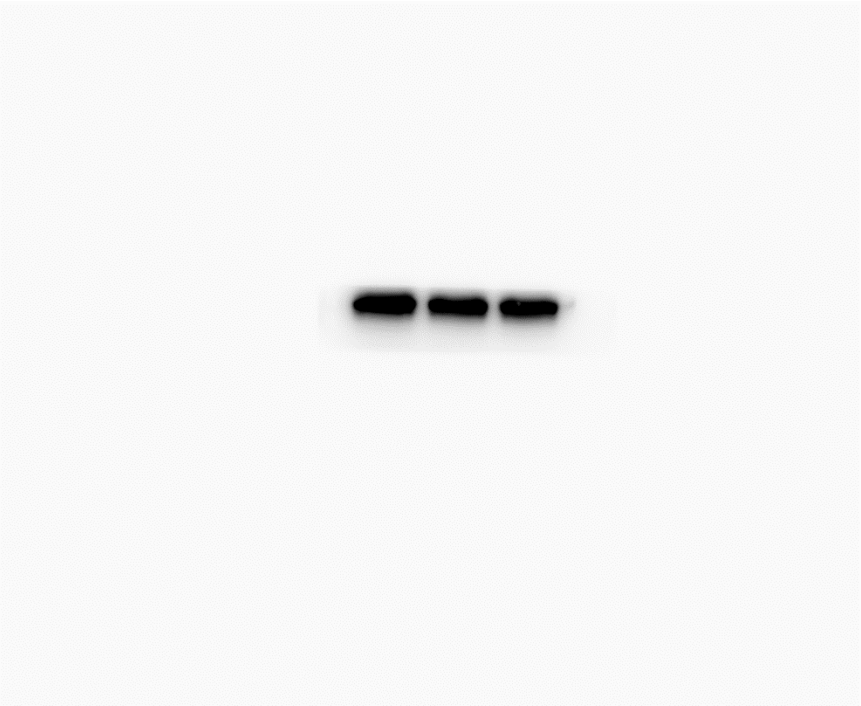
Full unedited blot for Supplemental Figure 9C AKT hCAF

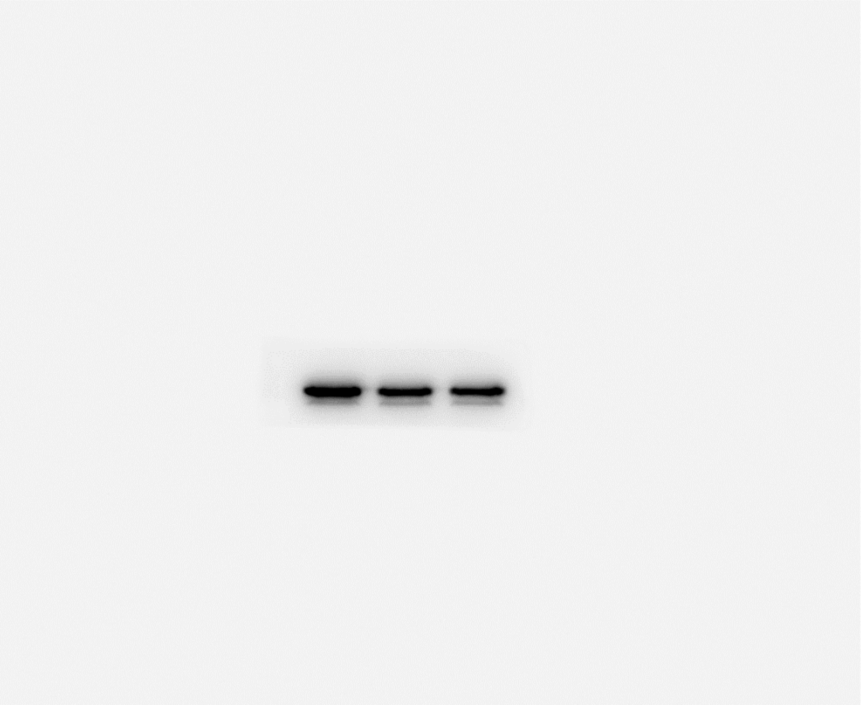

Full unedited blot for Supplemental Figure 9C p-AKT hCAF

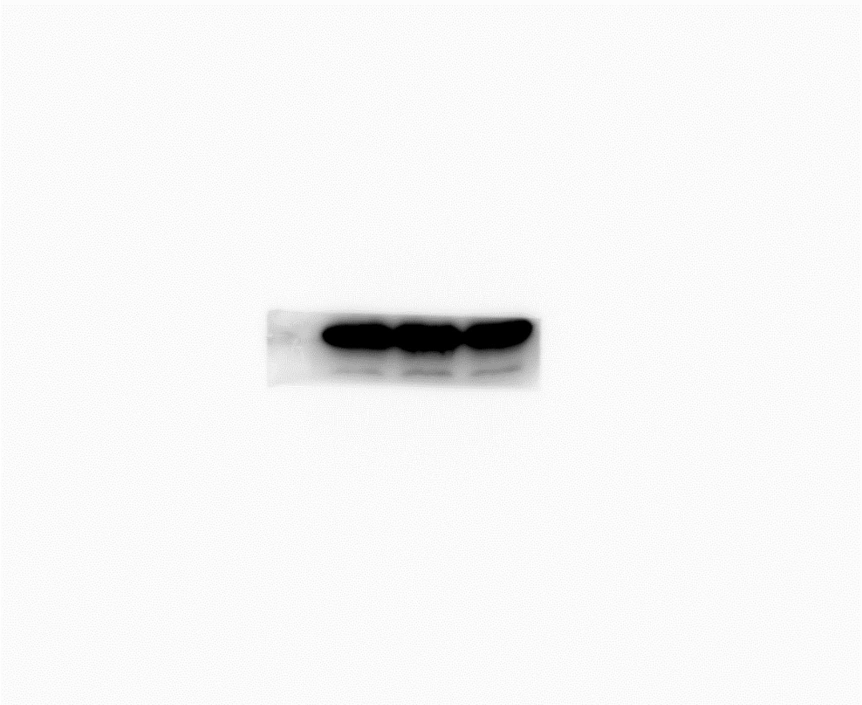

Full unedited blot for Supplemental Figure 9C GAPDH hCAF

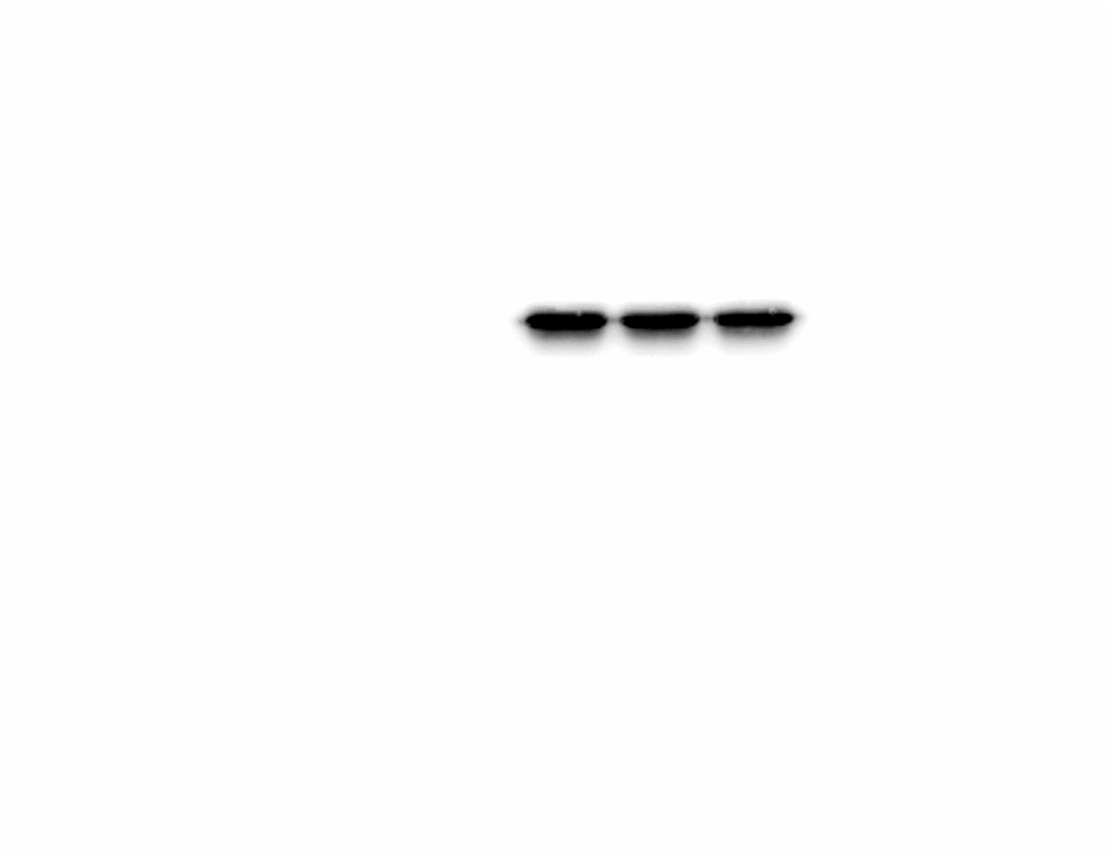

Full unedited blot for Supplemental Figure 9D AKT mCAF

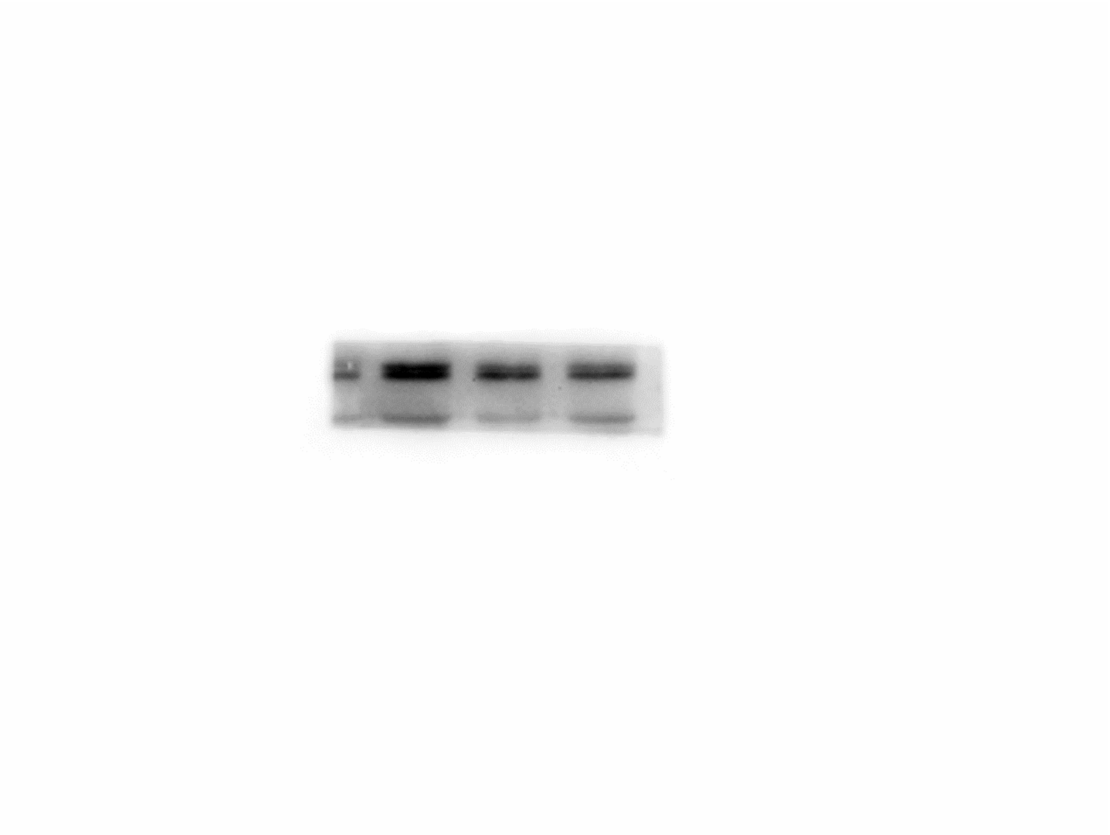

Full unedited blot for Supplemental Figure 9D p-AKT mCAF

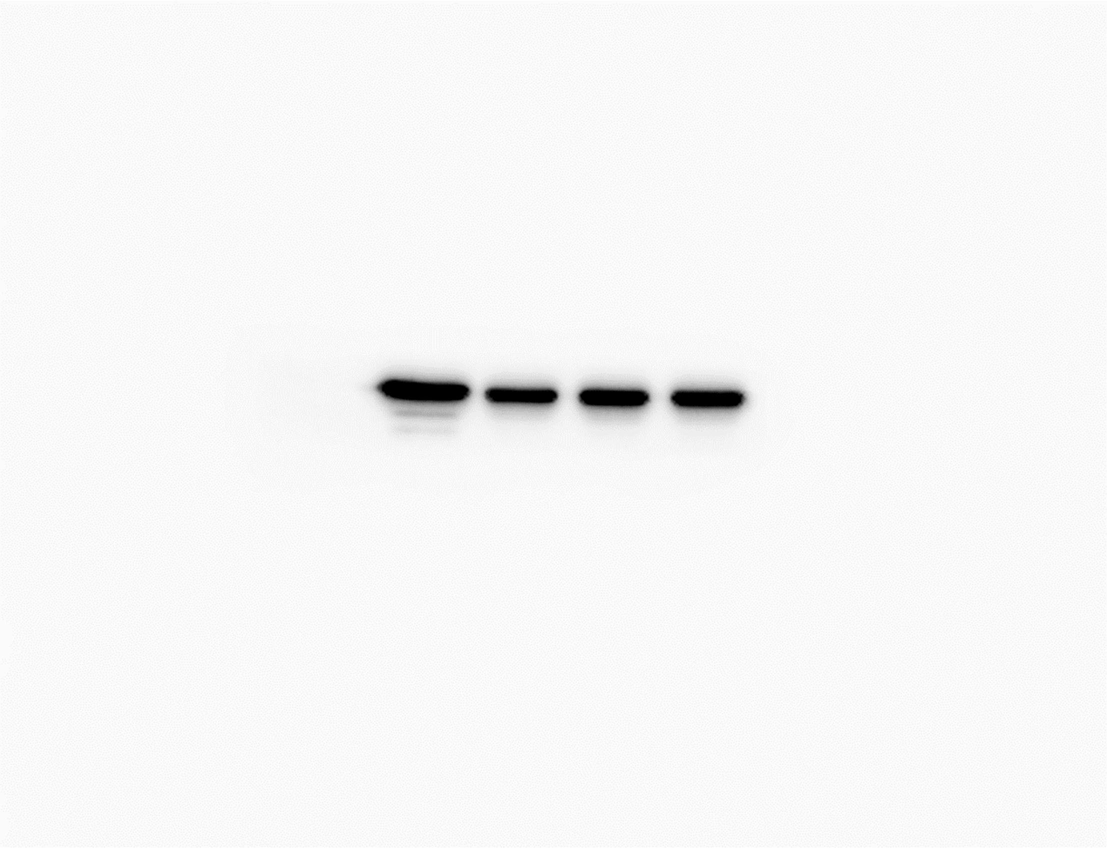

Full unedited blot for Supplemental Figure 9D GAPDH mCAF

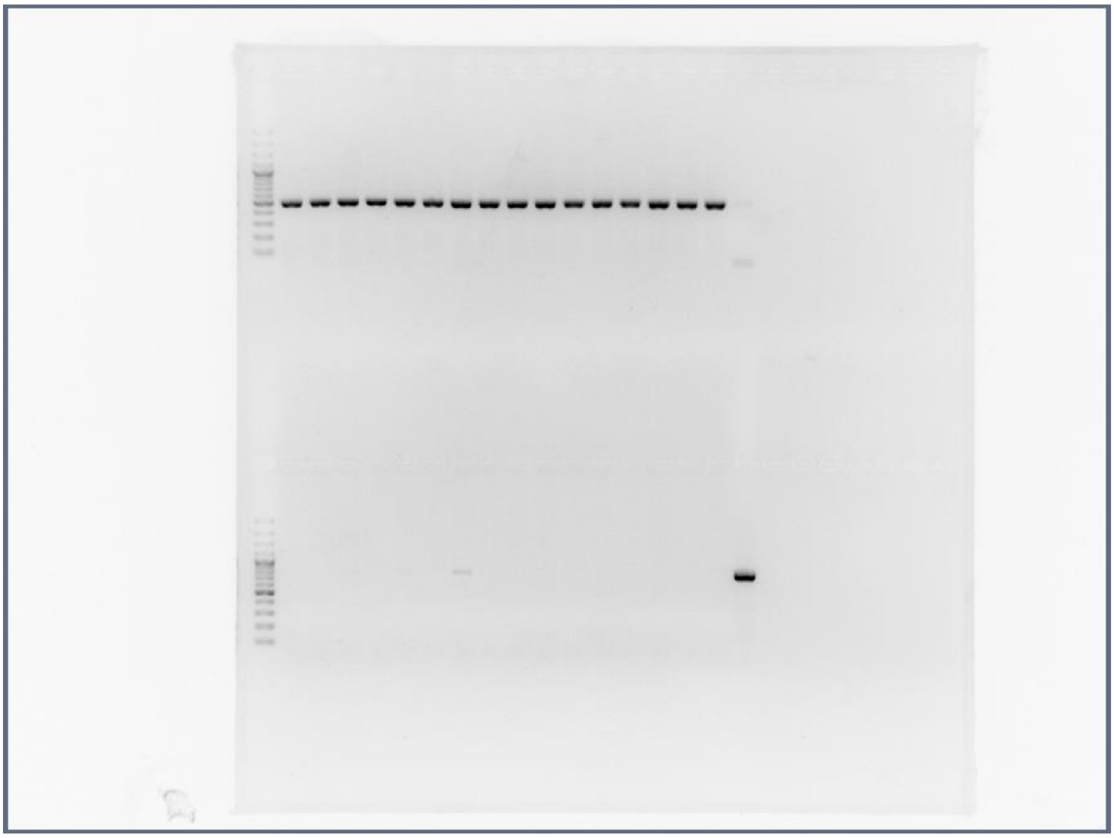

Full unedited gel for Supplemental Figure 3A

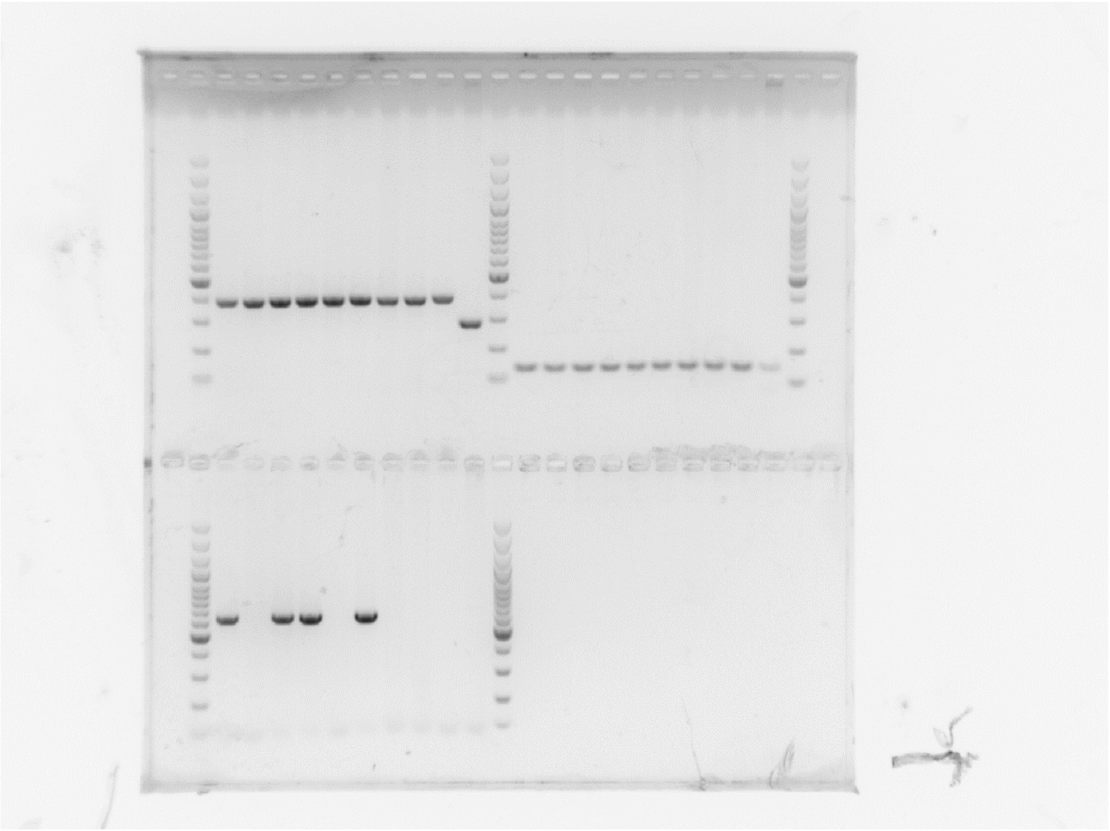

Full unedited gel for Supplemental Figure 4A
